# Tertiary Patents on Drugs Approved by the FDA

**DOI:** 10.1001/jamahealthforum.2025.5909

**Published:** 2026-01-02

**Authors:** Theodore W. Teng, S. Sean Tu, Helen Mooney, Liam Bendicksen, Sarah M. E. Gabriele, Olivier J. Wouters, William B. Feldman

**Affiliations:** 1 Harvard College, Cambridge, Massachusetts; 2 University of Alabama School of Law, Tuscaloosa; 3 Program On Regulation, Therapeutics, And Law, Division of Pharmacoepidemiology and Pharmacoeconomics, Department of Medicine, Brigham and Women’s Hospital, Boston, Massachusetts; 4 Yale Law School, New Haven, Connecticut; 5 Department of Health Services, Policy, and Practice, Brown University School of Public Health, Providence, Rhode Island; 6 Harvard Medical School, Boston, Massachusetts; 7 Division of Pulmonary and Critical Care Medicine, Department of Medicine, Brigham and Women’s Hospital, Boston, Massachusetts

## Abstract

**Importance:**

Manufacturers of drug-device combinations, such as inhalers and injectable medications, often obtain patents not just on the active pharmaceutical ingredients of these products (primary patents) but also on other features, such as their formulations and methods of use (secondary patents) and delivery devices (tertiary patents). Courts, policymakers, and regulators have recently begun scrutinizing whether manufacturers may be improperly listing tertiary patents with the Food and Drug Administration (FDA) that lack claims on active pharmaceutical ingredients and whether such patents may be delaying generic competition. However, the full scope of patenting practices on drug-device combinations remains unknown.

**Objective:**

To analyze patent protection on small-molecule drugs approved by the FDA from 1986 to 2023 with 1 or more tertiary patents.

**Design, Setting, and Participants:**

In this retrospective cohort study of patenting practices on drug-device combinations, all patents listed in the FDA’s *Approved Drug Products With Therapeutic Equivalence Evaluations* (Orange Book) were categorized (primary, secondary, or tertiary), and products with at least 1 tertiary patent were included. Analyses were performed between May 2024 and October 2025.

**Main Outcomes and Measures:**

The primary outcome of the study was the duration of expected patent protection on each product, measured from the time of approval until expiration of the last-to-expire patent. Added protection from tertiary patents that went beyond protection afforded by primary or secondary patents was also analyzed.

**Results:**

The FDA approved 331 products from 1986 to 2023 with 1 or more tertiary patents; 137 of 3241 patents (4.2%) listed on these products were primary patents, 1353 of 3241 were secondary patents (41.7%), and 1751 of 3241 were tertiary patents (54.0%). Among tertiary patents, 1047 of 1751 (59.8%) lacked claims making any mention of active pharmaceutical ingredients. The median (IQR) duration of expected patent protection among products in the cohort was 17.6 (14.4-21.2) years. There were 180 products (54.4%) that had tertiary patents extending periods of expected protection beyond other patents, and the median (IQR) duration of added protection was 7.5 (2.8-13.9) years.

**Conclusions and Relevance:**

The findings of this cohort study suggest that policymakers and regulators should take steps to ensure that tertiary patents are not improperly listed in the Orange Book and that generic competition occurs in a timely fashion.

## Key Points

**Question:**

How have medical device patents contributed to periods of market exclusivity on drug-device combinations?

**Findings:**

In this cohort study of 331 drug-device combinations approved by the Food and Drug Administration (FDA) from 1986 to 2023, 1751 of 3241 individual patent listings were on the delivery devices of these products. Medical device patents extended periods of protection for 180 products (54.4%) by a median (IQR) of 7.5 (2.8-13.9) years, with most device patents failing to mention the active pharmaceutical ingredients in their claims.

**Meaning:**

Patenting strategies on drug-device combinations may impede generic entry and keep prices high for patients and payers.

## Teaser

This cohort study analyzes patent protection on small-molecule drugs approved by the US Food and Drug Administration from 1986 to 2023 with 1 or more tertiary patents.

## Introduction

Patents may cover a broad range of features on medications, including their active ingredients (primary patents), formulations and methods of use (secondary patents), and delivery devices (tertiary patents).^1,2,3,4^ When manufacturers list key patents with the Food and Drug Administration (FDA) in what is known as the *Approved Drug Products With Therapeutic Equivalence Evaluations* (Orange Book), the agency cannot approve generic versions for marketing until these patents expire or are successfully challenged in court.^5^ Pharmaceutical patents promote innovation by protecting inventions from competition for time-limited periods and have been vital for spurring therapeutic advances in medicine.^6,7^

In some cases, however, companies may obtain patents on peripheral features of their products that can serve to delay generic competition and undermine access to low-cost therapies for patients.^2,8,9,10^ Drug-device combinations, such as inhalers, injectables, and other products that contain active pharmaceutical ingredients sold together with their delivery devices, may be particularly vulnerable to anticompetitive patenting practices, because they often include a wide range of potentially patentable features, from mechanical dose counters and spray valves to pen needles and polymer mesh. Prior studies have found that manufacturers list a large number of tertiary patents on inhalers for asthma and chronic obstructive pulmonary disease^11,12,13,14,15^ and glucagon-like peptide-1 injector pens for diabetes and weight loss.^16,17^ These tertiary patents have contributed to long periods of market exclusivity and high prices for patients and payers.^14,18^

Several recent court cases have raised questions about the permissibility of listing certain tertiary patents in the Orange Book. Although the FDA does not review what companies choose to list and instead serves in a ministerial role, the statute governing the Orange Book only permits submission of patents that claim the drug or a method of using the drug (the claims of a patent define the specific boundaries of the invention’s legal protections).^5^ A 2020 court ruling found that a patent on the mechanical drive mechanism of the Lantus SoloStar injector pen had been improperly listed, because it did not claim insulin.^19^ A 2024 ruling similarly found that several tertiary patents on the delivery device of ProAir HFA were improperly listed, because they did not claim albuterol.^20^ The Federal Trade Commission (FTC) has issued a series of letters calling on manufacturers to delist tertiary patents that make no mention of active ingredients in their patent claims (device-only patents) on the grounds that these patents do not satisfy the statutory criteria for permissible listings.^21,22,23,24^

Despite increasing legal scrutiny, the scale of tertiary patenting remains unknown, because academic researchers and regulators have largely focused on a handful of high-revenue products in asthma, chronic obstructive pulmonary disease, diabetes, and weight loss.^11,12,13,14,15,16,17,25,26,27^ Other types of drug-device combinations are marketed in the US, such as transdermal patches, ophthalmic implants, intravaginal rings, and nasal sprays, and manufacturers have listed tertiary patents covering a range of product features, from nanotechnology in orally administered drugs to dissolvable films in sublingual agents. Because neither the FDA nor the FTC maintains an exhaustive list of drug-device combinations (or their tertiary patents), efforts to further define and delineate patenting practices on these products are needed.

We reviewed and classified every patent in the Orange Book on drugs approved by the FDA from 1986 to 2023 and constructed a cohort of therapies with 1 or more tertiary patents. We analyzed the prevalence of tertiary patents on these products, the extent to which these tertiary patents have claimed active pharmaceutical ingredients, and the role that they have played in extending periods of market exclusivity.

## Methods

This cohort study followed the Strengthening the Reporting of Observational Studies in Epidemiology (STROBE) reporting guidelines. Institutional review board approval was not required because the study did not involve human participants.

### Cohort Identification

We identified all therapies approved by the FDA from 1986 to 2023 with 1 or more tertiary patents listed in the Orange Book (eMethods in Supplement 1). Drugs were defined at the level of the new drug application (NDA), meaning that different strengths or formulations included under the same NDA were considered as a single drug. A tertiary patent on any strength or formulation under the NDA qualified the drug for inclusion in our cohort.

To ensure that we properly categorized patents as tertiary (and excluded patents focused on other aspects of a product that happened to peripherally mention a delivery device), we required that patents meet 2 criteria, both focusing on patent claims. First, the subject of 1 or more patent claims had to be on the delivery device itself (eg, the inhaler or injector pen). Second, most claims (>50%) had to either cover the device or be related to the device. Claims were considered related to a device if they described a method of administering, manufacturing, producing, or tracking the device, or if they described a formulation that specifically referenced the device (see the eMethods in Supplement 1 for a complete description and eTable 1 in Supplement 1 for examples of how borderline cases were categorized). Prior studies have applied similar criteria when analyzing device patents.^11,13,16,25^

Although most tertiary patents claim inhaled, injectable, and other nonoral modes of delivery, some claim oral modes, including patents on nanotechnology, electronic tracking technology, microparticles, liposomes, osmotic delivery devices, and sustained-release polymers. We refer to all products with 1 or more tertiary patents, regardless of the delivery route, as drug-device combinations.

### Data Extraction

For each drug-device combination, we identified the date of FDA approval and route of administration using labels from the Drugs@FDA database.^28^ We determined therapeutic areas using the Anatomical Therapeutic Chemical Classification System database.^29^

Annual editions of the Orange Book through 2024 were used to identify all patents listed on each product. We classified patents as primary (on the active pharmaceutical ingredient), secondary (on formulations, methods of use, or any other aspect of the product besides the active pharmaceutical ingredient or delivery device), or tertiary (according to the criteria outlined herein). Because drugs were defined at the level of the NDA, any patent listed multiple times on different strengths or formulations under the same NDA were counted only once.

We used the US Patent and Trademark Office (USPTO) PatentsView Database to determine the filing date for each patent. The filing date indicates when a given patent application was submitted to the USPTO for review.

### Statistical Analysis

Our primary outcome was the duration of expected patent protection, measured as the time from FDA approval of a product until expiration of its last-to-expire patent. We also analyzed added protection from tertiary patents that went beyond protection afforded by other types of patents (primary and secondary), and we separately analyzed 2 phases of tertiary patent protection: protection from tertiary patents that referenced active pharmaceutical ingredients in their claims and protection from tertiary patents that made no such mentions (eMethods in Supplement 1). This distinction has become an important test applied by courts and the FTC when evaluating the permissibility of listing patents in the Orange Book.

Because generic drugs can enter the market even when FDA-listed patents are still active (in the case of successful challenges), we conducted sensitivity analysis analyzing the duration of actual protection, which was defined as the time from FDA approval until first generic entry or expiration of the last-to-expire patent (whichever occurs first). The timing of generic entry was determined using publicly available Medicaid data (eMethods in Supplement 1).^30^

As secondary outcomes, we measured the total number of patents per product, the mix of patent types by product, the proportion of tertiary patents mentioning active pharmaceutical ingredients in their claims, and the timing of patenting filings. We also performed a series of subgroup analyses stratifying outcomes by therapeutic class and route of administration. All analyses were completed in Python (version 3.11; Python Software Foundation) and Excel (version 16.93; Microsoft) between May 2024 and October 2025.

## Results

The FDA approved 331 products from 1986 to 2023 with 1 or more tertiary patents listed in the Orange Book. Of these, 82 (24.8%) were injectables, 75 (22.7%) were oral tablets or capsules, 60 (18.1%) were topicals, 56 (16.9%) were inhaled agents, 14 (4.2%) were intranasal sprays, 14 (4.2%) were ophthalmic preparations, 11 (3.3%) were intravaginal products, and 19 (5.7%) were agents delivered via other routes (including intrauterine, rectal, and dental routes). Seventy products (21.1%) were approved for the treatment of neurological conditions, 52 (15.7%) for respiratory conditions, 43 (13.0%) for genitourinary conditions, 41 (12.4%) for gastrointestinal and metabolic conditions, 37 (11.2%) for cancer or autoimmune disease, 23 (6.9%) for cardiovascular conditions, 18 (5.4%) for dermatologic conditions, and 47 (14.2%) for other conditions (including sensory and hormonal disorders) (eTable 2 in Supplement 1).

### Patent Portfolios

Manufacturers listed 3241 patents on the 331 products in the cohort. Primary patents accounted for 137 (4.2%), secondary patents for 1353 (41.7%), and tertiary patents for 1751 (54.0%) of all patent listings. Although patents on multiple strengths of the same drug were only counted once, some patents were listed on multiple drug-device combinations; for example, 22 different injector-pen patents each appeared on at least 5 separate products from the same company, and 6 different inhaler patents each appeared on at least 5 separate products from another company (eTable 3 in Supplement 1). The 3241 total patent listings corresponded to 2163 distinct patents, of which 90 (4.2%) were primary, 1108 (51.2%) were secondary, and 965 (44.6%) were tertiary patents.

The median (interquartile range [IQR]) number of patents per product in the cohort was 7 (3-15), with a median (IQR) of 0 (0-0) primary patents, 3 (1-6) secondary patents, and 2 (1-6) tertiary patents (eTable 4 in Supplement 1). The products with the highest median (IQR) number of tertiary patents were inhaled agents (7 [2-12]) and injectables (6 [1-15]) (Table 1). When stratified by therapeutic class, respiratory products had the highest median (IQR) number of tertiary patents (6 [2-12]), followed by gastrointestinal/metabolic agents (5 [1-18]), and neurologic drugs (3 [1-5]) (Table 2). Manufacturers exclusively listed tertiary patents (and no other patent types) on 43 products in the cohort (eTable 5 in Supplement 1).

**Table 1.** Characteristics of Patents on Drug-Device Combinations by Route of Administration, 1986 to 2023

Route of administration	Drugs, No.	Total patents, No.	Patents, No. (%)			
			Primary	Secondary	Tertiary patents referencing active ingredients	Tertiary patents without reference to active ingredient
Injection	82	1108	58 (5.2)	336 (30.3)	236 (21.3)	478 (43.1)
Inhalation	56	823	44 (5.3)	287 (34.9)	154 (18.7)	338 (41.1)
Oral	75	576	25 (4.3)	359 (62.3)	99 (17.2)	93 (16.1)
Topical	60	348	0	169 (48.6)	119 (34.2)	60 (17.2)
Intranasal	14	118	5 (4.2)	59 (50.0)	18 (15.3)	36 (30.5)
Ophthalmic	14	107	2 (1.9)	63 (58.9)	31 (29.0)	11 (10.3)
Other	8	53	2 (3.8)	23 (43.4)	20 (37.7)	8 (15.1)
Intravaginal	11	48	0	28 (58.3)	15 (31.3)	5 (10.4)
Intrauterine	4	21	0	4 (19.0)	3 (14.3)	14 (66.6)
Periarticular	1	16	0	12 (75.0)	3 (18.8)	1 (6.3)
Otic	1	8	0	7 (87.5)	1 (12.5)	0
Rectal	2	5	0	3 (60.0)	2 (40.0)	0
Urethral	1	5	0	3 (60.0)	1 (20.0)	1 (20.0)
Intracranial	1	3	1 (33.3)	0	1 (33.3)	1 (33.3)
Dental	1	2	0	0	1 (50.0)	1 (50.0)

**Table 2.** Characteristics of Patents on Drug-Device Combinations by Therapeutic Class, 1986 to 2023

Therapeutic class^a^	Drugs, No.	Total patents, No.	Patents, No. (%)			
			Primary	Secondary	Tertiary patents referencing active ingredient	Tertiary patents without reference to active ingredients
Respiratory system	52	704	43 (6.1)	222 (31.5)	121 (17.2)	318 (45.2)
Nervous system	70	651	15 (2.3)	332 (51.0)	165 (25.3)	139 (21.4)
Alimentary tract and metabolism	41	600	42 (7.0)	192 (32.0)	36 (6.0)	330 (55.0)
Antineoplastic and immunomodulating agents	37	384	9 (2.3)	208 (54.2)	113 (29.4)	54 (14.1)
Genitourinary system and sex hormones	43	232	1 (0.4)	96 (41.4)	83 (35.8)	52 (22.4)
Cardiovascular system	23	151	6 (4.0)	55 (36.4)	49 (32.5)	41 (27.2)
Dermatologicals	18	128	0	75 (58.6)	20 (15.6)	33 (25.8)
Sensory organs	16	117	2 (1.7)	72 (61.5)	32 (27.4)	11 (9.4)
Various	7	101	0	14 (13.9)	63 (62.4)	24 (23.8)
Systemic hormonal preparations^b^	10	76	5 (6.6)	29 (38.2)	8 (10.5)	34 (44.7)
Anti-infectives for systemic use	7	58	10 (17.2)	30 (51.7)	9 (15.5)	9 (15.5)
Blood and blood forming organs	4	32	3 (9.4)	25 (78.1)	4 (12.5)	0
Musculoskeletal system	1	2	0	1 (50.0)	0	1 (50.0)

^a^ Two products lacked data on therapeutic class.

^b^ Systemic hormonal preparations exclude sex hormones and insulins.

### Timing of Patent Filings

Among the 3241 patents listed on the 331 products in our analysis, 2381 (73.5%) were filed before FDA approval of the product, and 860 (26.5%) were filed after FDA approval. There were 194 products (58.6%) with at least 1 patent filed after FDA approval (median [IQR], 3 [1-6]) (eTable 6 in Supplement 1). The timing of patent filings relative to FDA approval differed by patent type. Overall, 133 of 137 primary patents (97.1%) were filed before FDA approval, compared with 987 of 1353 secondary patents (72.9%) and 1261 of 1751 tertiary patents (72.0%) (Table 3).

**Table 3.** Patent Filings Relative to US Food and Drug Administration (FDA) Approval of Drug-Device Combinations, 1986 to 2023

Patent type	Total patent listings, No.	Patents, No. (%)	
		Filed before FDA approval	Filed after FDA approval
Primary	137	133 (97.1)	4 (2.9)
Secondary	1353	987 (72.9)	366 (27.1)
Tertiary with claims referencing active pharmaceutical ingredients	704	520 (73.9)	184 (26.1)
Tertiary with no claims referencing active pharmaceutical ingredients	1047	741 (70.8)	306 (29.2)
All patents, No.	3241	2381	860

### Characteristics of Tertiary Patents

Among 1751 tertiary patents, 1047 (59.8%) lacked claims making any mention of the active pharmaceutical ingredients in the products on which they were listed. If such patents were removed from the Orange Book, the median (IQR) number of patents per drug-device combination would have fallen from 7 (3-15) to 5 (2-9). Of 1047 tertiary patent listings with no mention of active ingredients, 466 (44.5%) were set to expire in 2026 or later.

### Expected Patent Protection

The median (IQR) duration of expected patent protection among products in the cohort—from the time of FDA approval to expiration of the last-to-expire patent—was 17.6 (14.4-21.2) years. The routes of administration with the longest median (IQR) durations of expected protection were injectables (19.7 [15.6-25.2] years), intranasal products (18.6 [16.6-19.6] years), and inhaled agents (18.0 [15.4-21.7] years) (eTable 7 in Supplement 1). The therapeutic classes with the longest median (IQR) durations of protection were anticancer and immunomodulating agents (19.8 [15.9-27.9] years), gastrointestinal/metabolic drugs (19.1 [15.6-26.2] years), and cardiovascular agents (18.3 [13.0-20.6] years) (eTable 8 in Supplement 1).

### Role of Tertiary Patents in Extending Periods of Patent Protection

**Figure 1.**
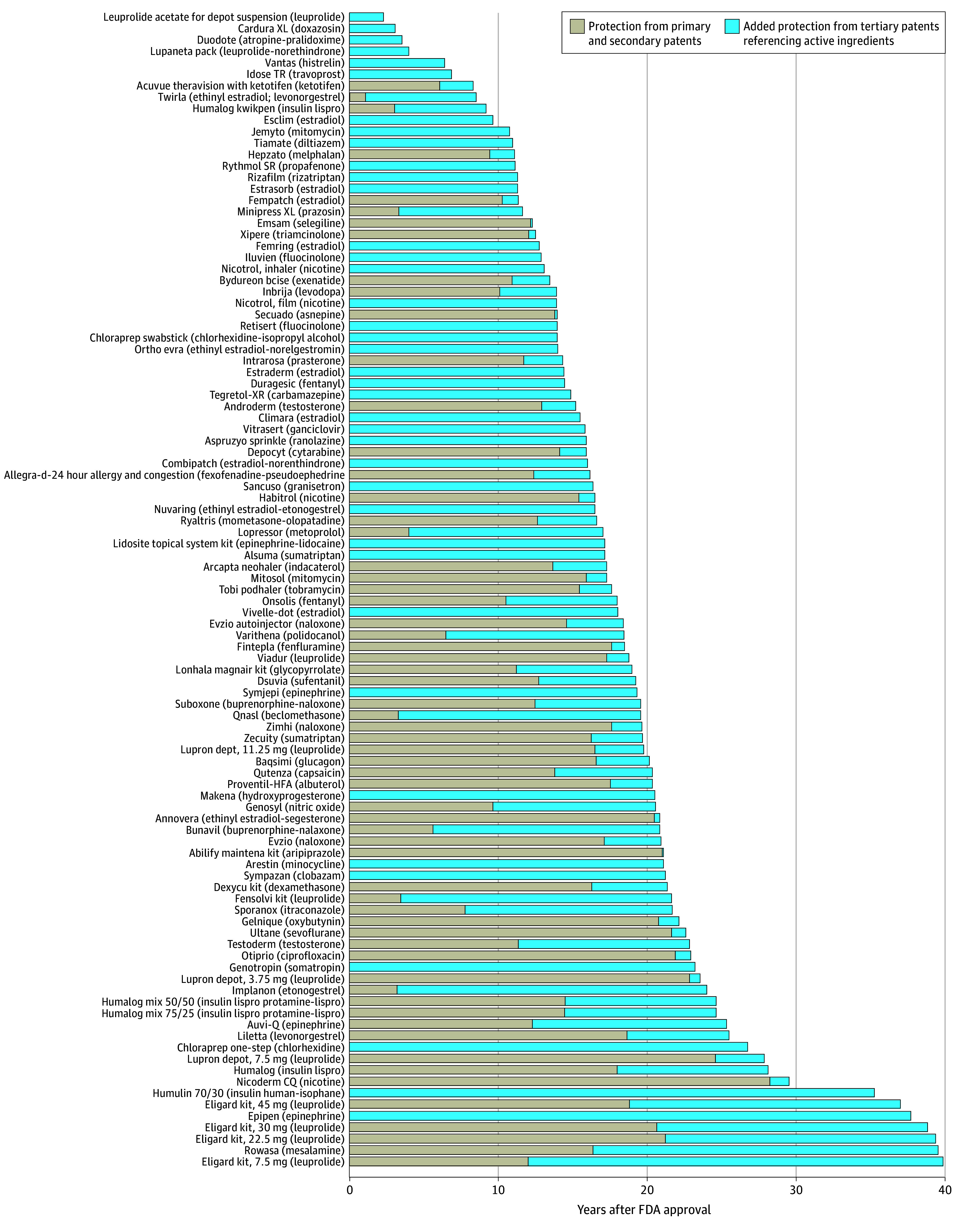
Patent Protection on Products With Last-to-Expire Tertiary Patents Referencing Active Ingredients FDA indicates US Food and Drug Administration.

**Figure 2.**
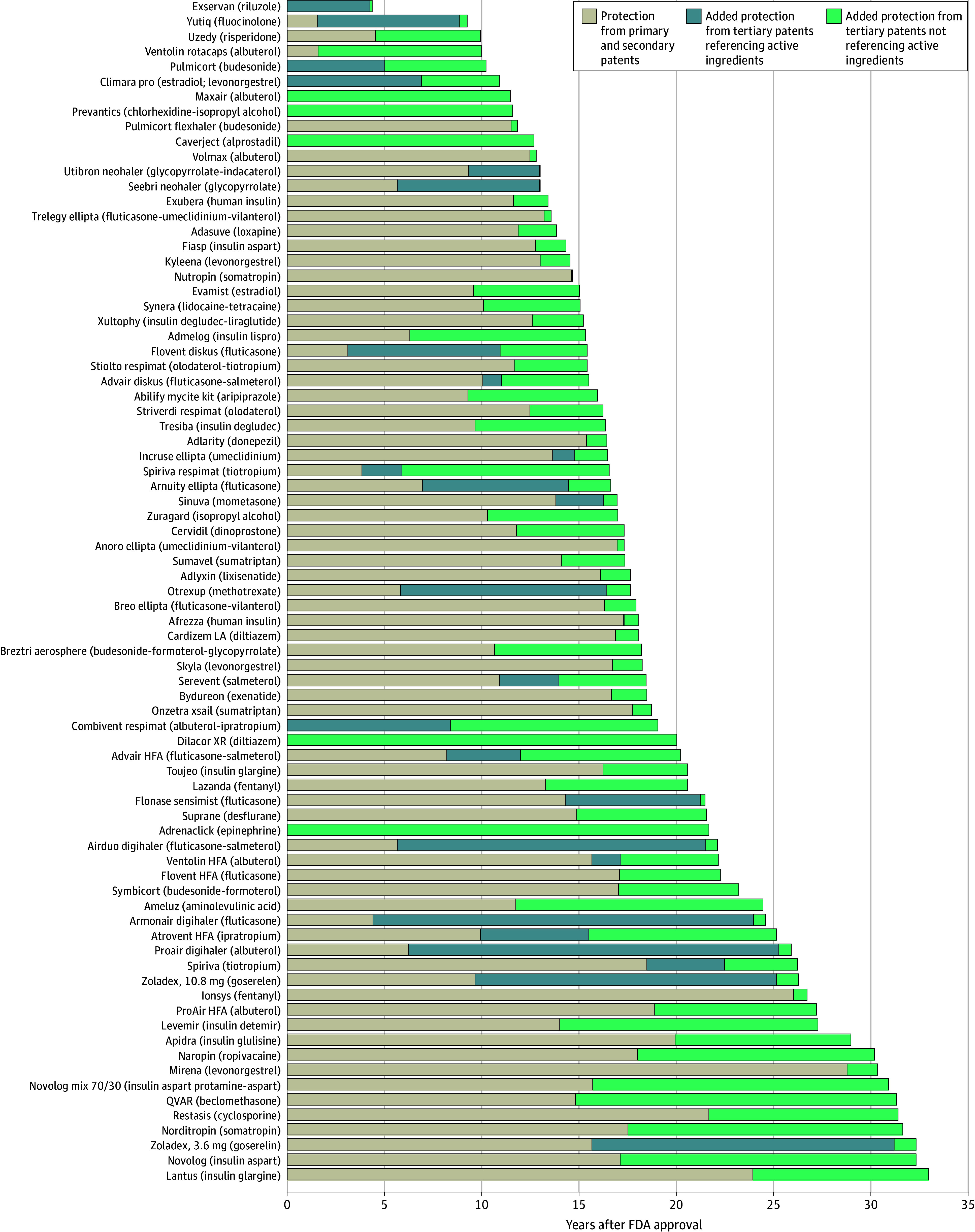
Patent Protection on Products With Last-to-Expire Tertiary Patents Making No Mention of Active Ingredients FDA indicates US Food and Drug Administration.

The median (IQR) duration of expected protection from primary and secondary patents was 14.6 (9.5-18.8) years. There were 180 products (54.4%) in the cohort that had tertiary patents extending periods of expected protection beyond primary and secondary patents, and the median (IQR) duration of added protection was 7.5 (2.8-13.9) years. Of these products, 101 (56.1%) had last-to-expire patents referencing active pharmaceutical ingredients, extending protection by a median (IQR) of 9.6 (3.1-15.3) years from the last-to-expire primary or secondary patent (Figure 1). Seventy-nine products (43.9%), by contrast, had last-to-expire patents that made no mention of active pharmaceutical ingredients in their claims, extending protection by a median (IQR) of 4.3 (1.2-8.3) years beyond all other patents (Figure 2).

### Sensitivity Analysis

When accounting for early generic entry, the median (IQR) duration of market exclusivity for products in the cohort was 16.5 (13.0-20.4) years. The number of products with tertiary patents that extended market exclusivity beyond primary and secondary patents dropped from 180 products (54.4%) to 171 products (51.7%). Of these, the last-to-expire tertiary patents on 97 products (56.7%) referenced active pharmaceutical ingredients (median [IQR] duration of extension beyond primary or secondary patents, 7.8 [3.1-14.9] years), whereas the last-to-expire tertiary patents on 74 (43.3%) did not (median [IQR] duration of extension beyond all other patents, 3.9 [1.1-7.5] years).

## Discussion

Over the past 4 decades, brand-name pharmaceutical firms have listed numerous tertiary patents with the FDA covering a wide range of drug-device combinations. Twenty-eight percent of these patents were filed after FDA approval, and 59.8% failed to mention active pharmaceutical ingredients in their claims. Such patents not only increased the density of patent portfolios on drug-device combinations, which can raise entry costs for generic firms, but they also extended the duration of protection, which can force generic competitors to file challenges long after brand-name approval.

The findings of this cohort study underscore how manufacturers have obtained and listed tertiary patents not only on top-selling drug-device combinations, like inhalers,^11,12,13,14,15^ glucagon-like peptide-1,^16,17^ and insulin,^25,26^ but on a wide spectrum of medications used for birth control, migraines, allergic rhinitis, chronic pain, schizophrenia, prostate cancer, psoriasis, and many other conditions. Contrary to claims that tertiary patents generally do not contribute to longer periods of market exclusivity,^31^ we found that more than half of all products in our cohort had tertiary patents that extended expected protection, with a median (IQR) extension of 7.5 (2.8-13.9) years. The overall median (IQR) duration of market exclusivity among drugs in our cohort (16.5 [13.0-20.4] years, even after accounting for early generic entry) is substantially greater than the median for small-molecule drugs in other studies, which was 14 years.^32^

Our findings provide regulators and policymakers with both a conceptual framework for analyzing drug-device combinations and a set of specific patent listings that may warrant further scrutiny. Recent action by the FTC has focused on a narrow set of products and targeted currently listed device-only patents. Future efforts could examine a broader range of drug-device combinations; indeed, 44.5% of device-only patents in our cohort will expire after the start of 2026 (though some of these have subsequently been delisted in response to FTC demands).^33^ Lawmakers and regulators should also review past listings, which may represent antitrust violations according to FTC policy statements.^21^

Another important set of measures would seek to prevent improper patent listings before they appear in the Orange Book. The FDA, for example, could publish more specific guidance regarding the types of patents that manufacturers can and cannot submit. Congress could also grant the FDA explicit authority and resources to evaluate and potentially reject Orange Book submissions, converting the ministerial role of the agency into one of more substantive review, and could even curtail the quantity of permitted listings or the number of patents that may be enforced in litigation. One bipartisan bill now before Congress would allow firms to enforce just 1 patent per family among closely related patents when suing generic firms for infringement.^34^

Pharmaceutical manufacturers have raised concerns that regulatory efforts to prevent improper patent listings could undermine innovation and disrupt a delicate regulatory balance designed to promote generic competition while protecting intellectual property.^20^ However, decreased financial rewards for more minor product modifications (eg, adding a dose counter to an inhaler) could help catalyze resource allocation toward more meaningful discovery (eg, identifying a novel drug based on a new molecular pathway). Given that the 965 distinct tertiary patents in our cohort represent less than 10% of the more than 10 000 patents listed in the Orange Book during the study period, more vigorous enforcement is unlikely to significantly disturb the current regulatory system.^3^ Instead of stifling innovation or compromising the incentive structures of current regulatory schemes, efforts to limit impermissible tertiary patent listings could facilitate more timely generic competition while protecting market exclusivity periods tied to foundational discoveries.

Addressing impermissibly listed patents is 1 component of an array of actions needed to facilitate generic competition on drug-device combinations in the US. Such actions could include further streamlining approval processes at the FDA and offering new incentives to generic firms for developing these complex products. Drug-device combinations represent a growing share of top-selling products, including 40% of the 50 top-selling drugs in Medicare by gross spending.^2^ Efforts to facilitate generic entry could reduce out-of-pocket costs and premiums for patients, strengthen adherence, and promote better clinical outcomes.^35,36,37^ Although other recent pharmaceutical reforms, such as Medicare drug price negotiation and out-of-pocket caps under the Inflation Reduction Act, represent valuable steps toward improved affordability, these measures apply to a limited set of drugs and only to a single payer. Generic competition exerts price-lowering effects across the US market; reforms to enhance such competition, which enjoy bipartisan support, are vital for ensuring that patients can afford their medications.

### Limitations

This cohort study had some limitations. First, we focused on small-molecule drugs because patents on biologic therapies are not comprehensively listed with the FDA. Second, we only considered Orange Book–listed patents, as these confer special regulatory protections for brand-name firms. The FDA must delay approval of generic drugs for marketing until such patents expire or are successfully challenged, and brand-name manufacturers can also trigger automatic 30-month stays in generic approval by suing for infringement.^38^ However, manufacturers can still seek to enforce patents that are not listed in the Orange Book. Third, we examined whether tertiary patents met a key test applied by courts and the FTC of at least mentioning an active pharmaceutical ingredient in their claims. However, merely mentioning active pharmaceutical ingredients may prove insufficient for courts as litigation continues to unfold, in which case our analysis could underestimate the scale of potentially impermissible patent listings. By contrast, courts could conclude that some tertiary patents that include other types of claims (eg, method of treatment claims) may be permissible even if the patent fails to mention the active ingredients of the product, in which case our analysis could overstate the scale of potentially impermissible patent listings. Fourth, our analysis focused on drug-device combinations with at least 1 tertiary patent; we did not examine patent portfolios on drug-device combinations with only primary and secondary patents listed in the Orange Book. Fifth, we did not examine the extent to which manufacturers listed patents from the same family (continuation patents) or how such patents contributed to the density of patent portfolios. Finally, because many patents on products in our cohort expire in the future (such that we cannot observe early generic entry), we may overestimate realized market exclusivity periods. However, because we could not observe the addition of future patents, we could also be underestimating realized market exclusivity periods.

## Conclusions

Brand-name manufacturers frequently obtain tertiary patents covering a range of technologies on drug-device combinations. Many such patents may be improperly listed with the FDA. Regulators and policymakers should continue pursuing efforts to rein in problematic patenting strategies and ensure that patients have timely access to low-cost generic drugs.

## Notes

Supplement 1. eMethods eTable 1. Examples of how patents on the borderline between secondary and tertiary were categorized eTable 2. Products approved by the FDA with one or more tertiary patents, 1986-2023 eTable 3. Patents listed on multiple drug-device combinations, 1986-2023 eTable 4. Patent portfolios of drug-device combinations approved by the FDA, 1986-2023 eTable 5. Products with portfolios consisting solely of tertiary patents eTable 6. Patents filed after FDA approval eTable 7. Patent protection on drug-device combinations by route of administration eTable 8. Patent protection on drug-device combinations by therapeutic class

Supplement 2. Data Sharing Statement

## References

[1] Beall RF, Kesselheim AS. Tertiary patenting on drug-device combination products in the United States. Nat Biotechnol. 2018;36(2):142-145. doi:10.1038/nbt.4078 29406508

[2] Feldman WB. Patent thickets and product hops: challenges and opportunities for legislative reform. J Law Med Ethics. 2025;1-6. doi:10.1017/jme.2025.54 40197405

[3] Tu SS, Kesselheim AS, Wetherbee K, Feldman WB. Changes in the number of continuation patents on drugs approved by the FDA. JAMA. 2023;330(5):469-470. doi:10.1001/jama.2023.11525 37526728 PMC10394575

[4] Horrow C, Gabriele SME, Tu SS, Sarpatwari A, Kesselheim AS. Patent portfolios protecting 10 top-selling prescription drugs. JAMA Intern Med. 2024;184(7):810-817. doi:10.1001/jamainternmed.2024.0836 38739386 PMC11091822

[5] US Food and Drug Administration. Report to Congress: The Listing of Patent Information in the Orange Book. Accessed October 14, 2025. https://www.fda.gov/media/155200/download

[6] Garthwaite C. Economic markets and pharmaceutical innovation. J Econ Perspect. 2025;39(2):3-26. doi:10.1257/jep.20251438

[7] Hemphill CSSB. Patents, innovation, and competition in pharmaceuticals: the Hatch-Waxman Act after 40 years. J Econ Perspect. 2025;39(2):27-52. doi:10.1257/jep.20241423

[8] Feldman R. May your drug price be evergreen. J Law Biosci. 2018;5(3):590-647. doi:10.1093/jlb/lsy022 31143456 PMC6534750

[9] Feldman R. Drugs, Money, and Secret Handshakes: The Unstoppable Growth of Prescription Drug Prices. Cambridge University Press. 2019. doi:10.1017/9781108687676

[10] Sinha M. Costly gadgets: barriers to market entry and price competition for generic drug-device combinations in the United States. Minn J Law Sci Technol. 2022;23(1):293-361.

[11] Feldman WB, Bloomfield D, Beall RF, Kesselheim AS. Patents and regulatory exclusivities on inhalers for asthma and COPD, 1986-2020. Health Aff (Millwood). 2022;41(6):787-796. doi:10.1377/hlthaff.2021.01874 35579925 PMC10328096

[12] Reddy S, Beall RF, Tu SS, Kesselheim AS, Feldman WB. Patent challenges and litigation on inhalers for asthma and COPD. Health Aff (Millwood). 2023;42(3):398-406. doi:10.1377/hlthaff.2022.00873 36877911

[13] Demkowicz BJ, Tu SS, Kesselheim AS, Carrier MA, Feldman WB. Patenting strategies on inhaler delivery devices. Chest. 2023;164(2):450-460. doi:10.1016/j.chest.2023.02.031 36842533 PMC10475818

[14] Feldman WB, Tu SS, Alhiary R, Kesselheim AS, Wouters OJ. Manufacturer revenue on inhalers after expiration of primary patents, 2000-2021. JAMA. 2023;329(1):87-89. doi:10.1001/jama.2022.19691 36594955 PMC9857605

[15] Feldman WB, Bloomfield D, Beall RF, Kesselheim AS. Brand-name market exclusivity for nebulizer therapy to treat asthma and COPD. Nat Biotechnol. 2022;40(9):1319-1325. doi:10.1038/s41587-022-01451-7 36085503 PMC10591455

[16] Alhiary R, Kesselheim AS, Gabriele S, Beall RF, Tu SS, Feldman WB. Patents and regulatory exclusivities on GLP-1 receptor agonists. JAMA. 2023;330(7):650-657. doi:10.1001/jama.2023.13872 37505513 PMC11457043

[17] Alhiary R, Gabriele S, Kesselheim AS, Tu SS, Feldman WB. Delivery device patents on GLP-1 receptor agonists. JAMA. 2024;331(9):794-796. doi:10.1001/jama.2024.0919 38315473 PMC10845039

[18] Wouters OJ, Feldman WB, Tu SS. Product hopping in the drug industry–lessons from albuterol. N Engl J Med. 2022;387(13):1153-1156. doi:10.1056/NEJMp2208613 36155425

[19] In re Lantus Direct Purchaser Antitrust Litigation, No 18-206. Accessed October 14, 2025. https://www.govinfo.gov/app/details/USCOURTS-mad-1_16-cv-12652

[20] Teva Branded Pharmaceutical Products R&D, Inc. et al v. Amneal Pharmaceuticals of New York, LLC et al. Accessed October 14, 2025. https://litigationtracker.law.georgetown.edu/litigation/teva-branded-pharmaceutical-products-rd-inc-et-al-v-amneal-pharmaceuticals-of-new-york-llc-et-al/

[21] Federal Trade Commission. FTC Issues Policy Statement on Brand Pharmaceutical Manufacturers’ Improper Listing of Patents in the Food and Drug Administration’s ‘Orange Book’. Accessed October 14, 2025. https://www.ftc.gov/news-events/news/press-releases/2023/09/ftc-issues-policy-statement-brand-pharmaceutical-manufacturers-improper-listing-patents-food-drug

[22] Federal Trade Commission. FTC Expands Patent Listing Challenges, Targeting More Than 300 Junk Listings for Diabetes, Weight Loss, Asthma and COPD Drugs. Accessed October 14, 2025. https://www.ftc.gov/news-events/news/press-releases/2024/04/ftc-expands-patent-listing-challenges-targeting-more-300-junk-listings-diabetes-weight-loss-asthma

[23] Federal Trade Commission. FTC Challenges More Than 100 Patents as Improperly Listed in the FDA’s Orange Book. Accessed October 14, 2025. https://www.ftc.gov/news-events/news/press-releases/2023/11/ftc-challenges-more-100-patents-improperly-listed-fdas-orange-book

[24] Federal Trade Commission. FTC Renews Challenge of More Than 200 Improper Patent Listings. Accessed October 14, 2025. https://www.ftc.gov/news-events/news/press-releases/2025/05/ftc-renews-challenge-more-200-improper-patent-listings

[25] Olsen A, Beall RF, Knox RP, Tu SS, Kesselheim AS, Feldman WB. Patents and regulatory exclusivities on FDA-approved insulin products: a longitudinal database study, 1986-2019. PLoS Med. 2023;20(11):e1004309. doi:10.1371/journal.pmed.1004309 37971985 PMC10653475

[26] Van de Wiele VL, Kesselheim AS, Beran D, Darrow JJ. Insulin products and patents in the USA in 2004, 2014, and 2020: a cross-sectional study. Lancet Diabetes Endocrinol. 2023;11(2):73-75. doi:10.1016/S2213-8587(22)00354-0 36702563

[27] Luo J, Kesselheim AS. Evolution of insulin patents and market exclusivities in the USA. Lancet Diabetes Endocrinol. 2015;3(11):835-837. doi:10.1016/S2213-8587(15)00364-2 26453281

[28] US Food and Drug Administration. Drugs@FDA: FDA-Approved Drugs. Accessed October 14, 2025. https://www.accessdata.fda.gov/scripts/cder/daf/index.cfm

[29] Norwegian Institute for Public Health. Application for ATC codes. Accessed October 14, 2025. https://atcddd.fhi.no/atc/application_for_atc_codes/

[30] Centers for Medicare & Medicaid Services. Medicaid Drug Rebate Program (MDRP). Accessed October 14, 2025. https://www.medicaid.gov/medicaid/prescription-drugs/medicaid-drug-rebate-program

[31] US Government Accountability Office. Generic Drugs: Stakeholder Views on Improving FDA’s Information on Patents. GAO-23-105477. Accessed October 14, 2025. https://www.gao.gov/products/gao-23-105477

[32] Rome BN, Lee CC, Kesselheim AS. Market exclusivity length for drugs with new generic or biosimilar competition, 2012-2018. Clin Pharmacol Ther. 2021;109(2):367-371. doi:10.1002/cpt.1983 32654122

[33] Capitol Forum. Teva: Drugmaker Requests Delisting of Inhaler Patents from Orange Book in Response to Court Order; Boehringer Ingelheim, Novo Nordisk Have Also Asked FDA to Pull Disputed Drug-Device Patents. Accessed October 14, 2025. https://thecapitolforum.com/teva-inhaler-patents-orange-book/

[34] United States Senate. Welch, Hawley, Klobuchar Introduce Bipartisan Legislation to Streamline Drug Patent Litigation, Lower Cost of Prescription Drugs. Accessed October 14, 2025. https://www.welch.senate.gov/welch-hawley-klobuchar-introduce-bipartisan-legislation-to-streamline-drug-patent-litigation-lower-cost-of-prescription-drugs/

[35] Assistant Secretary for Planning and Evaluation. Trends in Prescription Drug Spending, 2016-2021. Accessed October 14, 2025. https://aspe.hhs.gov/sites/default/files/documents/88c547c976e915fc31fe2c6903ac0bc9/sdp-trends-prescription-drug-spending.pdf

[36] Dave CV, Kesselheim AS, Fox ER, Qiu P, Hartzema A. High generic drug prices and market competition: a retrospective cohort study. Ann Intern Med. 2017;167(3):145-151. doi:10.7326/M16-1432 28672324

[37] Fusco N, Sils B, Graff JS, Kistler K, Ruiz K. Cost-sharing and adherence, clinical outcomes, health care utilization, and costs: a systematic literature review. J Manag Care Spec Pharm. 2023;29(1):4-16. doi:10.18553/jmcp.2022.21270 35389285 PMC10394195

[38] US Food and Drug Administration. Patent Certifications and Suitability Petitions. Accessed October 14, 2025. https://www.fda.gov/drugs/abbreviated-new-drug-application-anda/patent-certifications-and-suitability-petitions

