# Supplementary material for: Tertiary Patents on Drugs Approved by the FDA
Source: JAMA Health Forum. 2026 Jan 2;7(1):e255909. doi: 10.1001/jamahealthforum.2025.5909 (PMC12761334; doi:10.1001/jamahealthforum.2025.5909)
Supplement: Supplement 1. — eMethods eTable 1. Examples of how patents on the borderline between secondary and tertiary were categorized eTable 2. Products approved by the FDA with one or more tertiary patents, 1986-2023 eTable 3. Patents listed on multiple drug-device combinations, 1986-2023 eTable 4. Patent portfolios of drug-device combinations approved by the FDA, 1986-2023 eTable 5. Products with portfolios consisting solely of tertiary patents eTable 6. Patents filed after FDA approval eTable 7. Patent protection on drug-device combinations by route of administration eTable 8. Patent protection on drug-device combinations by therapeutic class [file jamahealthforum-e255909-s001.pdf]

Supplemental Online Content

Teng TW, Tu SS, Mooney H, et al. Tertiary patents on drugs approved by the FDA. *JAMA Health Forum.* 2025;7(1):e255909. doi:10.1001/jamahealthforum.2025.5909

eMethods
eTable 1. Examples of how patents on the borderline between secondary and tertiary were categorized
eTable 2. Products approved by the FDA with one or more tertiary patents, 1986-2023
eTable 3. Patents listed on multiple drug-device combinations, 1986-2023
eTable 4. Patent portfolios of drug-device combinations approved by the FDA, 1986-2023
eTable 5. Products with portfolios consisting solely of tertiary patents
eTable 6. Patents filed after FDA approval
eTable 7. Patent protection on drug-device combinations by route of administration
eTable 8. Patent protection on drug-device combinations by therapeutic class

© 2025 Teng TW, et al. *JAMA Health Forum.*

eMethods

<u>Cohort Identification</u>

Patents listed on drugs approved in a given year first appear in the Food and Drug Administration's (FDA) *Approved Drug Products with Therapeutic Equivalence Evaluations* (Orange Book) in the annual edition published the following year. For example, patents on drugs approved in 1986 first appear in the 1987 annual edition of the Orange Book. Our study period began in 1986, because 1987 marks the beginning of consecutive annual editions of the Orange Book stretching through the present (no Orange Book was released in 1986). The study period ended with drug approvals in 2023 to enable observation of patents listed in the 2024 edition of the Orange Book, the last year of data when the study was begun.

We classified patents as tertiary only when two criteria were met. First, the patent had to contain at least one claim on the delivery device. Such claims could include those on the composition of the device (e.g., a polymer comprising the transdermal patch in question). Second, a majority of claims had to be related to the device—these included claims covering methods for administering the device, methods for manufacturing or producing the device, methods describing how the device functions or tracking how it works, and formulations specifically referencing the device. Claims relating to methods of administration (e.g., delivering medications to the lungs) were considered distinct from claims relating to methods of treatment, prevention, or diagnosis (e.g., treating asthma); only the former were considered device-related.

All patents were manually reviewed by the authors. One author (TT) characterized the claims of all patents listed in the Orange Book with the support of another author (SST). A third author (WBF) then reviewed all patents that made any mention of a delivery device in their claims (i.e., all potential tertiary patents). Difficult cases were collectively adjudicated by

© 2025 Teng TW, et al. *JAMA Health Forum.*

consensus of three authors (TT, SST, and WBF). See **eTable 1** for examples of how borderline cases were categorized.

Diagnostic (as opposed to therapeutic) agents were excluded. The 9 diagnostic agents excluded from the cohort were Xenoview (xenon XE-129 hyperpolarized) for ventilation-perfusion scans, Netspot (gallium dotatate GA-68) for positron emission topography (PET) imaging, Cardiogen (rubidium chloride RB-82) for PET imaging, Minitec (technetium TC-99 sodium pertechinate) for PET imaging, Optison (albumin human) for echocardiograms, Gastromark (ferumoxsil) for use in magnetic resonance imaging (MRI), Spy Agent Green Kit (indocyanine green) for fluorescence imaging, Imagent (dimyristoyl lecithin-perflexane) for echocardiograms, and Breathtek/Meretek UBT Kit (urea, C-13) for urea breath tests. Two drugs, both copper intrauterine contraceptives, were excluded due to missing information regarding the timing of regulatory approval.

Drugs were defined at the level of the New Drug Application (NDA). Thus, different strengths or formulations included under the same NDA were considered as a single drug. Closely related NDAs with the same active ingredients were counted separately.

A small number of products in the cohort, including insulin-containing drug-device combinations, were reclassified by the FDA in 2020 as biologics rather than small-molecule drugs and thus were removed from the Orange Book.[1] These products were included in our analysis; however, patents potentially added on these products in the final 4 years of the study period (2021-2024) could not be observed (though we were able to analyze all patents on these products during the prior 34 years from 1987-2020). Undercounting late-filed patents could lead to an underestimate of patent protection on these products.

© 2025 Teng TW, et al. *JAMA Health Forum.*

Claims mentioning active ingredients

Drug manufacturers must only list patents in the Orange Book that: (1) claim the drug and are patents on the drug substance (active ingredient) or drug product (formulation or composition); or (2) claim a method of using such a drug.[2] The FTC has called on manufacturers to delist "device-only" patents that fail to mention active ingredients in their claims, as these may constitute violations of the legal criteria for listing.[3] We assessed all tertiary patents to determine whether active ingredients (or molecular structures of active ingredients) were mentioned in the patent claims. Some tertiary patents could have contained method of use claims (e.g., a method of treating diabetes); we considered these tertiary patents "device-only" if the claims failed to mention the active ingredients of the drug.

Duration of patent protection

When analyzing added patent protection from tertiary patents, we first determined the patent protection on primary and secondary patents by subtracting the FDA approval date from the last-to-expire primary or secondary patent. Patent expiration as determined based on the most recent available Orange Book included in the study (through the 2024 annual edition). We then determined the added protection from tertiary patents refencing active pharmaceutical ingredients by subtracting the expiration date of the last-to-expire primary or secondary patent from the expiration date of the last-to-expire tertiary patent referencing the active pharmaceutical ingredient. When a product had no primary or secondary patents, the duration of added protection from tertiary patents referencing the active pharmaceutical ingredient was counted as the time from the FDA approval date to the expiration date of the last-to-expire tertiary patent referencing the active pharmaceutical ingredient. Finally, we measured the added protection from

© 2025 Teng TW, et al. *JAMA Health Forum.*

tertiary patents that did not reference an active ingredient. To do this, we subtracted the expiration date of the last-to-expire tertiary patent referencing the active pharmaceutical compound—or, when no such patent was listed, the last-to-expire primary or secondary patent (or, when no such patents were listed, the FDA approval date)—from the expiration date of the last-to-expire tertiary patent making no reference to the active pharmaceutical compound. If a patent was delisted from the Orange Book before its expiration date (through the 2024 edition of the Orange Book), we removed that patent listing from our analysis of patent protection.

Generic entry

To determine the first generic entry for each NDA, we used publicly available Medicaid data.[4] The first marketing date in Medicaid could slightly lag commercial launch in some cases but is a good proxy for launch dates in the US.

© 2025 Teng TW, et al. *JAMA Health Forum.*

eTable 1: Examples of how patents on the borderline between secondary and tertiary were categorized

Patent number	Patent title	Claims	Categorization and rationale
4,588,580	Transdermal administration of fentanyl and device therefof	“We claim:<br>1. A process for inducing and maintaining analgesia which comprises administering through an area of intact skin, a skin permeable form of a material selected from the group consisting of fentanyl and its analgetically effective derivatives at an analgetically effective rate and continuing the administration of said material at said rate for an extended period of time at least sufficient to induce analgesia.<br>2. The process for claim 1 further comprising the coadministration with said material of a skin permeation enhancer for said material.<br>3. The process of claim 1 wherein said extended period of time is in the range of from 12 hours to 7 days.<br>4. The process of claim 1 wherein the steady state administration rate of said material is maintained within the range of about 10 to 300 μg/hr for a substantial portion of said extended period of time.<br>5. The process of claim 1 wherein said area of intact skin is within the range of about 5 to 100 cm^2 and said material is delivered through the skin at a rate within the range of about 0.5 to 10 $\mu g/cm^2$ /hr.<br>6. The process of claim 5 in which said area is in the range of from about 10 to 50 cm^2 and said rate is in the range of about 1 to 5 $\mu g/cm^2$ /hr.<br>7. The process of claim 4 wherein the steady state<br>8. The process of claim 2 wherein said permeation enhancer is ethanol.<br>9. The process of claim 1 wherein said material is the base form of a material selected from the group consisting of fentanyl, sufentanyl, carfentanyl, lofentanyl, and alfentanyl.<br>10. The process of claim 2 wherein the material is fentanyl base.<br>11. The process of claim 3 wherein the material is fentanyl base.<br>12. The process of claim 4 wherein the material is fentanyl base.<br>13. The process of claim 5 wherein the material is fentanyl base.<br>14. The process of claim 6 wherein the material is fentanyl base.<br>15. The process of claim 7 wherein the material is fentanyl base.<br>16. A process for the transdermal administration of a material selected from the group consisting of fentanyl and its analgetically effective derivatives which comprises:<br>(a) contacting a predetermined area of intact skin with a source of skin permeable form of said materia1;<br>(b) maintaining said source in material transmitting relationship to said area of intact skin for an administration period of at least 12 hours; and<br>(c) delivering said material into the skin at a rate within the range of about 0.1-10 $\mu g/cm^2$ /hour for at least about 12 hours.<br>17. The process of claim 16 further comprising maintaining said source at unit activity throughout said administration period.<br>18. The process of claim 17 wherein said predetermined area of intact skin is in the range of 5 to 100 cm^2.<br>19. The process of claim 16 wherein said material is the base form of a material selected from the group consisting of fentanyl, sufentanyl, carfentanyl, lofentanyl and alfentanyl.	**Tertiary**<br><br>At least one claim on the device itself (“a medical device for transdermal administration of a material”) and a majority of claims that were device-related.

© 2025 Teng TW, et al. JAMA Health Forum.

		20. The process of claim 18 further comprising coadministering a permeation enhancer for said material during said administration period.<br>21. The process of claim 16 wherein the material is fentanyl base.<br>22. A medical device for the transdermal administration of a material selected from the group consisting of fentanyl and its analgetically effective derivatives at an analgetically effective rate for an extended period of time of at least four hours which comprises:<br>(a) reservoir means containing a skin permeable form of said material in an amount sufficient to deliver said material at said analgetically effective rate for said extended period of time and,<br>(b) means for maintaining a said reservoir means in material transmitting relationship to intact skin.<br>23. The medical device of claim 22 wherein said system delivers the base form of the material through intact skin at a rate in the range of from about 10 to 300 µg/hr for a substantial portion of said period of time.<br>24. The medical device of claim 22 wherein said reservoir means contains a skin permeation enhancer for said material.<br>25. The medical device of claim 22 wherein said predetermined area is in the range of about 5-100 cm^2 and the rate of delivery of said material is in the range of about 0.5-10 $\mu g/cm^2$ /hr.<br>26. The medical device of claim 25 wherein said area is in the range of about 10-50 cm^2 and said delivery rate is in the range of about 1-5 $\mu g/cm^2$ /hr.<br>27. The medical device of claim 25 wherein said material is fentanyl base.<br>28. A medical device for continuous transdermal administration of a material selected from the group consisting of fentanyl and its analgetically effective, derivates comprising, in combination:<br>(a) a reservoir for said material having a skin proximal, material releasing surface area in the range of about 5-100 cm^2, said reservoir containing between 0.1 and 50% by weight of a skin permeable form of said material in amounts and at a concentration adequate to permit delivery of said material through intact human skin at a rate within the range of from 0.5 to 10 $\mu g/cm^2$ /hr for at least about 12 hours and<br>(b) means for maintaining said reservoir in material transmitting relationship to the skin.<br>29. The medical device of claim 28 wherein said means for maintaining said reservoir in material transmitting relationship to the skin is an amine resistant adhesive disposed in the flow path of the material from the reservoir to the skin.<br>30. The medical device of claim 28 further comprising release rate controlling means disposed in the flow path of said material to the skin which means limit the flux of material from said system to a level less than the flux of material through the skin to which it is applied.<br>31. The medical device of claim 17 wherein said material is the base form of a material selected from the group consisting of fentanyl, sufentanyl, carfentanyl, lofentanyl and alfentanyl.<br>32. The medical device of claim 17 wherein said reservoir contains an aqueous gel comprising up to about 47% of 95% ethanol, 1-10% gelling agent, 0.1-10% of said material, and release rate controlling means disposed between said reservoir and the skin, said release rate controlling means being more permeable to said material than ethanol.<br>33. The medical device of claim 31 wherein said material is fentanyl base.	

© 2025 Teng TW, et al. JAMA Health Forum.

		34. The medical device of claim 30 further comprising permeation enhancer means for increasing the permeability to said material of the skin to which said device is applied.<br>35. The medical device of claim 34 wherein said permeation enhancer means is admixed in said reservoir means.<br>36. The medical device system of claim 35 wherein said release rate controlling means restricts the flux of said material from said system substantially more than the flux of said permeation enhancer from said device.<br>37. The medical device of claim 30 wherein said reservoir is an aqueous gel comprising approximately from 0-47% of 95% ethanol., 1-10% gelling agent, 0.1-10% of said material.<br>38. The medical device of claim 37 wherein said aqueous gel comprises from approximately 20-35% of said ethanol, 1-5% gelling agent and 0.1-2% of said material.<br>39. The medical device of claim 38 wherein said release rate controlling means is substantially more permeable to ethanol than to said material.<br>40. The medical device of claim 39 wherein said material is initially contained in said reservoir at equilibrated levels no greater than 0.5 $\mu g/cm^2$.<br>41. The medical device of claim 39 wherein said means for maintaining said system on the skin is an amine resistant adhesive disposed on said release rate controlling means and said material is fentanyl.<br>42. The medical device of claim 41 wherein said surface area is in the range of from about 10-50 cm^2.<br>43. The medical device of claim 28 wherein said reservoir is an polymeric matrix having said material contained therein in an amount from about 5-50% by weight.<br>44. The medical device of claim 43 wherein said means for maintaining said device in material transmitting relationship to the skin comprises an amine resistant adhesive disposed in the flow path of material from the reservoir to the skin.<br>45. The medical device of claim 43 wherein said matrix is selected from the group consisting of polyisobutylene and silicone polymers.<br>46. The medical device of claim 45 wherein said device further comprises release rate controlling means disposed in the flow path of said material to the skin which limits the flux of said material from said device to a level less than the flux of material through the skin to which it is applied.<br>47. The medical device of claim 46 in which said means for maintaining said device in material transmitting relationship to the skin is an amine resistant adhesive disposed on said release controlling means.<br>48. The medical device of claim 47 wherein said material is fentanyl base.<br>49. The medical device of claim 28 wherein said release rate is in the range of from about 1-5 g/cm^2 /hr and said material is fentanyl.<br>50. The medical device of claim 30 wherein said release rate is in the range of from about 1-5 $\mu g/cm^2$ /hr and said material is fentanyl.<br>51. The medical device of claim 35 wherein said release rate is in the range of from about 1-5 $\mu g/cm^2$ /hr and said material is fentanyl base.<br>52. The medical device of claim 36 wherein said release rate is in the range of from about 1-5 $\mu g/cm^2$ /hr and said material is fentanyl.	

© 2025 Teng TW, et al. JAMA Health Forum.

		53. The medical device of claim 38 wherein said release rate is in the range of from about 1-5 μg/cm^2 /hr and said material is fentanyl.<br>54. The medical device of claim 40 wherein said release rate is in the range of from about 1-5 μg/cm^2 /hr and said material is fentanyl.<br>55. The medical device of claim 43 wherein said release rate is in the range of from about 1-5 μg/cm^2 /hr and said material is fentanyl.<br>56. The medical device of claim 44 wherein said release rate is in the range of from about 1-5 μg/cm^2 /hr and said material is fentanyl.<br>57. The medical device of claim 45 wherein said release rate is in the range of from about 1-5 μg/cm^2 /hr and said material is fentanyl.<br>58. The medical device of claim 47 wherein said release rate is in the range of from about 1-5 μg/cm^2 /hr and said material is fentanyl."	
5,723,147	TTS containing an antioxidant	"What is claimed is:<br>1. A pharmaceutical composition comprising:<br>(a) a therapeutically effective amount of (S)-N-ethyl-3-{(1-dimethylamino)ethyl}-N-methyl-phenyl-carbamate in free base or acid addition salt form (Compound A);<br>(b) about 0.01 to about 0.5 percent by weight of an antioxidant, based on the weight of the composition, and<br>(c) a diluent or carrier.<br>2. A pharmaceutical composition according to claim 1 containing 1 to 40% by weight of Compound A in free base or acid addition salt form.<br>3. A pharmaceutical composition according to claim 1 wherein the anti-oxidant is tocopherol, esters thereof, ascorbic acid, butylhydroxytoluene, butylhydroxyanisole or propyl gallate.<br>4. A pharmaceutical composition according to claim 1 wherein the anti-oxidant is α-tocopherol or ascorbyl palmitate.<br>5. A pharmaceutical composition according to claim 1 wherein the anti-oxidant is tocopherol and is present in an amount of 0.1% by weight based on the weight of the pharmaceutical composition.<br>6. A pharmaceutical composition according to claim 1 comprising<br>(a) Compound A in free base form in an amount of 20 to 40% by weight,<br>(b) polymethacrylate in an amount of 10 to 30% by weight,<br>(c) acrylate copolymer in an amount of 40 to 60% by weight, and<br>(d) α-tocopherol in an amount of between 0.05 and 0.3% by weight wherein the total weight of the pharmaceutical composition is 100%.<br>7. A transdermal device comprising a pharmaceutical composition as defined in claim 1, wherein the pharmaceutical composition is supported by a substrate.<br>8. A transdermal device according to claim 7, wherein the pharmaceutical composition is located between an adhesive layer and the substrate.<br>9. A transdermal device according to claim 8, wherein a release liner releasably contacts the adhesive layer.<br>10. The pharmaceutical composition of claim 1, further comprising silicone oil.<br>11. A transdermal device comprising a backing layer, a layer comprising a therapeutically effective amount of (S)-N-ethyl-3-{(1-dimethylamino)ethyl}-N-methyl-phenyl-carbamate (Compound A) and an amount of antioxidant effective to stabilize Compound A from degradation in a polymer matrix, a release-liner and, disposed between the layer comprising Compound A in a polymer matrix and the release-liner, a discrete layer of adhesive material for releasably fixing said transdermal device to a patient's skin.	**Secondary**<br><br>Although at least one claim on the device itself ("a transdermal device"), only a minority of claims were device-related.

© 2025 Teng TW, et al. JAMA Health Forum.

		12. The transdermal device of claim 1, wherein the discrete layer of adhesive material also comprises silicone oil.<br>13. The transdermal device of claim 1, wherein the antioxidant is tocopherol, esters thereof, ascorbic acid, butylhydroxytoluene, butylhydroxyanisole, or propyl gallate.<br>14. The transdermal device of claim 1, wherein the antioxidant is α-tocopherol or ascorbyl palmitate.<br>15. A method of stabilizing (S)-N-ethyl-3-{(1-dimethylamino)ethyl}-N-methyl-phenyl-carbamate in free base or acid addition salt form (Compound A), wherein the method comprises forming a composition by combining Compound A with an amount of anti-oxidant effective to stabilize Compound A from degradation.<br>16. A method according to claim 15, wherein the anti-oxidant is tocopherol, esters thereof, ascorbic acid, butylhydroxytoluene, butylhydroxyanisole or propyl gallate.<br>17. The method of claim 15, wherein the anti-oxidant is α-tocopherol or ascorbyl palmitate.<br>18. The method of claim 15, wherein the anti-oxidant is present in an amount of from about 0.01 to about 0.5% by weight based on the weight of the composition.<br>19. The method of claim 15, wherein α-tocopherol is present as the antioxidant in an amount of 0.1% by weight of the composition.<br>20. The method of claim 15, wherein the composition also comprises silicone oil."	
9,254,307	**Polymeric delivery formulations of leuprolide with improved efficacy**	"What is claimed is:<br>1. A flowable composition suitable for use to form an in situ, single body controlled release implant, the composition consisting essentially of a non-aqueous, liquid solution of: (a) a biodegradable thermoplastic copolymer of lactide and glycolide that has a carboxy terminal group, that is at least substantially insoluble in aqueous medium or body fluid, and that has a weight average molecular weight of 15,000 to 45,000; (b) N-methyl-2-pyrrolidone; and (c) leuprolide acetate, wherein the copolymer is present in the composition at 30 wt % to 50 wt %; the N-methyl-2- pyrrolidone is present in the composition at 50 wt % to 70 wt % and the leuprolide acetate is present in the composition at 2 wt % to 8 wt %; and the composition is formulated for administration to a male patient about once per month to produce in situ the single body implant thereby reducing serum testosterone levels in the male patient.<br>2. A biodegradable implant formed in situ, in a male patient, by the steps comprising: (a) injecting 0.2 to 0.5 ml of the flowable composition of claim 1 within the body of the patient; and (b) allowing the N-methyl-2-pyrrolidone to dissipate to produce a solid, monolithic biodegradable implant of a microporous matrix of a core surrounded by a skin.<br>3. A method of reducing serum testosterone levels in a male human comprising injecting subcutaneously into the male human 0.2 ml to 0.5 ml of a flowable composition consisting essentially of a non-aqueous, liquid solution of: (a) a biodegradable thermoplastic copolymer of lactide and glycolide that has a carboxy terminal group, that is at least substantially insoluble in aqueous medium or body fluid, and that has a weight average molecular weight of 15,000 to 45,000; (b) N-methyl-2-pyrrolidone; and (c) leuprolide acetate in an amount sufficient to reduce LHRH levels in a human; wherein the copolymer is present in the composition at 30 wt % to 50 wt %; the N-methyl-2-pyrrolidone is present in the composition at 50 wt % to 70	**Tertiary**<br><br>At least one claim on the device itself ("a biodegradable implant formed in situ") and a majority of claims that were device-related.

© 2025 Teng TW, et al. JAMA Health Forum.

		wt % and the leuprolide acetate is present in the composition at 2 wt % to 8 wt %;<br>whereupon the flowable composition forms in situ a monolithic solid implant of a microporous matrix of a core surrounded by a skin within a body tissue of the male human by diffusion of the N-methyl-2-pyrrolidone into the body fluid of the male human and precipitation of the copolymer of lactide and glycolide."	
9,522,155	**Synthetic bile acid compositions and methods**	"What is claimed is:<br>1. A composition comprising about 0.1% w/v to about 2% w/v deoxycholic acid (DCA):<br>(DCA)<br>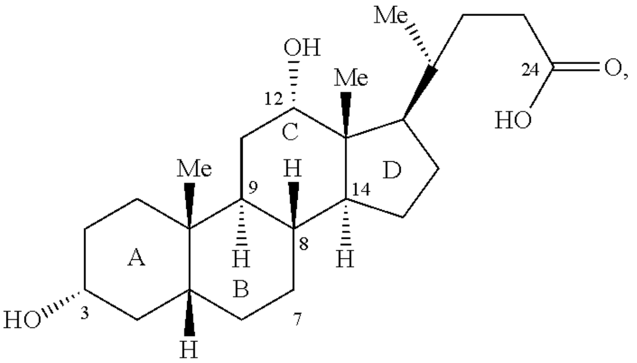<br><br>or a pharmaceutically acceptable salt thereof, wherein the DCA comprises a synthetic side chain of formula:<br>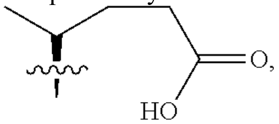<br><br>and at least one pharmaceutically acceptable excipient and/or carrier.<br>2. A method for removal of fat deposits in a mammal comprising administering to the mammal in need thereof a therapeutically effective amount of a composition comprising DCA<br>(DCA)<br>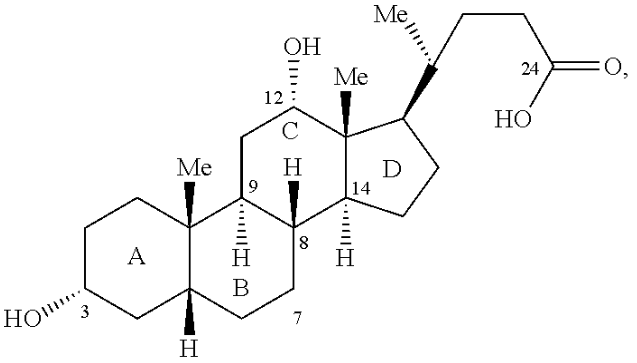<br><br>or a pharmaceutically acceptable salt thereof, wherein the DCA comprises a synthetic side chain of formula:<br>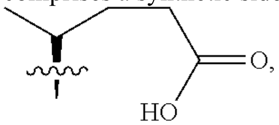<br><br>and at least one pharmaceutically acceptable excipient and/or carrier.<br>3. The method of claim 2, wherein the composition comprises about 0.1% w/v to about 2% w/v DCA or a pharmaceutically acceptable salt thereof.	**Secondary**<br><br>Although at least one claim on the device itself ("a syringe or syringe-loadable container"), only a minority of claims were device-related.

© 2025 Teng TW, et al. JAMA Health Forum.

		4. The method of claim 2, wherein the composition further comprises up to 5% w/v phosphatidylcholine.<br>5. The method of claim 2, wherein the composition does not contain phosphatidylcholine.<br>6. A syringe or syringe-loadable container comprising a composition comprising DCA:<br>(DCA)<br>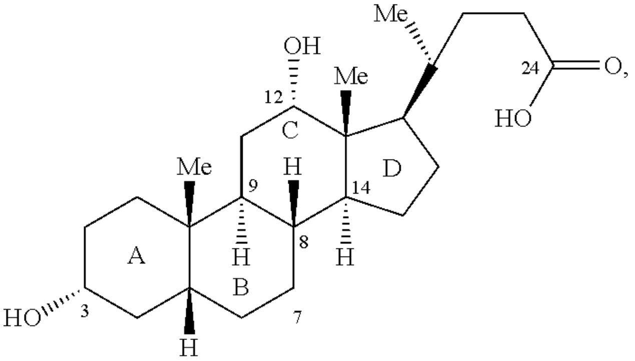<br><br>or a pharmaceutically acceptable salt thereof, wherein the DCA comprises a synthetic side chain of formula:<br>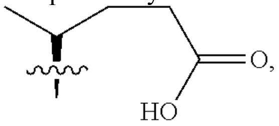<br><br>and at least one pharmaceutically acceptable excipient and/or carrier.<br>7. The syringe or syringe-loadable container of claim 6, wherein the composition comprises about 0.1% w/v to about 2% w/v DCA or a pharmaceutically acceptable salt thereof.<br>8. The syringe or syringe-loadable container of claim 6, wherein the composition further comprises up to 5% w/v phosphatidylcholine.<br>9. The syringe or syringe-loadable container of claim 6, wherein the composition does not contain phosphatidylcholine."	
10537581	**Vaginal inserted estradiol pharmaceutical compositions and methods**	"The invention claimed is:<br>1. A pessary consisting of:<br>a) a liquid pharmaceutical composition comprising:<br>about 1 µg to about 25 µg of estradiol; and<br>a solubilizing agent comprising a medium chain oil;<br>wherein the viscosity of the liquid pharmaceutical composition ranges from about 50 cP to about 1000 cP at 25 ° C., and<br>wherein the liquid pharmaceutical composition does not include a hydrophilic gel-forming bioadhesive agent; and<br>b) a soft gelatin capsule encapsulating the liquid pharmaceutical composition;<br>wherein estradiol is the only active hormone in the pessary; and wherein the pessary is for intravaginal administration.<br>2. The pessary of claim 1, wherein the liquid pharmaceutical composition comprises about 1 µg to about 10 µg of estradiol.<br>3. The pessary of claim 1, wherein the liquid pharmaceutical composition comprises about 10 µg to about 25 µg of estradiol.<br>4. The pessary of claim 1, wherein the estradiol is solubilized.<br>5. The pessary of claim 1, wherein the medium chain oil comprises at least one C6-C12 fatty acid or a glycol, monoglyceride, diglyceride, or triglyceride ester thereof.<br>6. The pessary of claim 5, wherein each glycol, monoglyceride, diglyceride, or triglyceride ester of the C6-C12 fatty acid is	**Tertiary**<br><br>At least one claim on the device itself ("a biodegradable implant formed in situ") and a majority of claims that were device-related.

© 2025 Teng TW, et al. JAMA Health Forum.

		independently an ester of caproic acid, an ester of caprylic acid, or an ester of capric acid.<br>7. The pessary of claim 6, wherein the triglyceride ester of the C6-C12 fatty acid is a caprylic/capric triglyceride.<br>8. A pessary consisting of:<br>a) a liquid pharmaceutical composition comprising:<br>about 1 μg to about 25 μg of estradiol;<br>a caprylic/capric triglyceride; and<br>a non-ionic surfactant comprising PEG-6 stearate and ethylene glycol palmitostearate;<br>wherein the viscosity of the liquid pharmaceutical composition ranges from about 50 cP to about 1000 cP at 25° C., and<br>wherein the liquid pharmaceutical composition does not include a hydrophilic gel-forming bioadhesive agent; and<br>b) a soft gelatin capsule encapsulating the liquid pharmaceutical composition, wherein estradiol is the only active hormone in the pessary; and wherein the pessary is for intravaginal administration.<br>9. A pessary consisting of:<br>(a) a liquid pharmaceutical composition comprising:<br>about 1 μg to about 25 μg of estradiol and<br>a solubilizing agent comprising a medium chain oil,<br>wherein the viscosity of the liquid pharmaceutical composition ranges from about 50 cP to about 1000 cP at 25 ° C., and<br>wherein the liquid pharmaceutical composition does not include a hydrophilic gel-forming bioadhesive agent;<br>and<br>(b) a soft gelatin capsule encapsulating the liquid pharmaceutical composition;<br>wherein estradiol is the only active hormone in the pessary; and wherein administration of the pessary to the proximal region of the vagina of a patient provides a therapeutically effective concentration of estradiol over 24 hours in the proximal region of the vagina.<br>10. A method of treating an estrogen-deficient state, the method comprising administering to a patient in need thereof a pessary according to claim 1.<br>11. A method of treating an estrogen-deficient state, the method comprising administering to a patient in need thereof, a pessary according to claim 8."	
11,154,513	**Semifluorinated compounds**	"The invention claimed is:<br>1. An ophthalmic composition comprising CF_3—$(CF_2)_3$—$CH(CH_3)$—$(CH_2)_2$—CH_3 and CF_3—$(CF_2)_3$—$(CH_2)_4$—CH_3.<br>2. The composition according to claim 1, wherein the composition consists of the CF_3—$(CF_2)_3$—$CH(CH_3)$—$(CH_2)_2$—CH_3 and the CF_3—$(CF_2)_3$—$(CH_2)_4$—CH_3.<br>3. The composition according to claim 1, being in liquid form and comprising at least 1 wt % of said compound CF_3—$(CF_2)_3$—$CH(CH_3)$—$(CH_2)_2$—CH_3 or from 1 wt % to 5 wt % of said compound CF_3—$(CF_2)_3$—$CH(CH_3)$—$(CH_2)_2$—CH_3, based on the total weight of the composition.<br>4. The composition according to claim 1, being formulated as a clear liquid solution.<br>5. The composition according to claim 1 being substantially free of:<br>(a) a polymer,<br>(b) a perfluorinated compound, and/or	**Secondary**<br><br>Although at least one claim on the device itself ("a kit comprising an ophthalmic composition according to claim 1 and a container for holding the composition, wherein said container comprises a dropper which

© 2025 Teng TW, et al. JAMA Health Forum.

		(c) a dissolved pharmacologically active ingredient which is not a semifluorinated alkane.<br>6. The composition of according to claim 1, wherein the composition is in liquid form and consists essentially of the compound CF_3—$(CF_2)_3$—$CH(CH_3)$—$(CH_2)_2$—CH_3 in an amount from 1 wt % to 5 wt % and the compound CF_3—$(CF_2)_3$—$(CH_2)_4$—CH_3 in an amount up to 95 wt %, based on the total weight of the composition.<br>7. The composition according to claim 1, being in liquid form and comprising from 1 wt % to 5 wt % of CF_3—$(CF_2)_3$—$CH(CH_3)$—$(CH_2)_2$—CH_3 and up to 95 wt % of CF_3—$(CF_2)_3$—$(CH_2)_4$—CH_3, based on the total weight of the composition.<br>8. The composition according to claim 7, being formulated as a clear liquid solution.<br>9. The composition according to claim 8 being substantially free of:<br>(a) a polymer,<br>(b) a perfluorinated compound, and/or<br>(c) a dissolved pharmacologically active ingredient which is not a semifluorinated alkane.<br>10. The composition of according to claim 9, wherein the composition consists essentially of the CF_3—$(CF_2)_3$—$CH(CH_3)$—$(CH_2)_2$—CH_3 and the CF_3—$(CF_2)_3$—$(CH_2)_4$—CH_3, and optionally one or more lipophilic liquid constituents.<br>11. A kit comprising an ophthalmic composition according to claim 1 and a container for holding the composition, wherein said container comprises a dropper which dispenses droplets having a volume of 8 to 15 μL topically to a lacrimal sac, lower eyelid, eye surface, or other ophthalmic tissue.<br>12. A method of treating a disease or condition of a patient in need of such treatment, comprising administering the composition according to claim 1 to the patient.<br>13. The method according to claim 12, wherein the disease or condition is dry eye disease (keratoconjunctivitis sicca) or a symptom or condition associated therewith; and/or Meibomian Gland Dysfunction (MGD), or a symptom or condition associated therewith.<br>14. The method according to claim 13, wherein the composition is topically administered into the lacrimal sac, into the lower eyelid, to an eye surface or to an ophthalmic tissue.<br>15. The method according to claim 12, wherein the disease or condition is corneal damage."<br>16. A method of treating a disease or condition of a patient in need of such treatment, comprising administering the composition according to claim 10 to the patient.<br>17. The method according to claim 16, wherein the disease or condition is dry eye disease (keratoconjunctivitis sicca) or a symptom or condition associated therewith; and/or Meibomian Gland Dysfunction (MGD), or a symptom or condition associated therewith.<br>18. The method according to claim 16, wherein the disease or condition is corneal damage."	dispenses droplets"), only a minority of claims were device-related.

© 2025 Teng TW, et al. JAMA Health Forum.

eTable 2: Products approved by the FDA with one or more tertiary patents, 1986-2023

Brand name	Generic name	NDA	FDA approval date	Route of administration	Therapeutic class
ABILIFY MAINTENA KIT	aripiprazole	202971	2/28/13	Injection	N – Nervous system
ABILIFY MYCITE KIT	aripiprazole	207202	11/13/17	Oral	N – Nervous system
ABRAXANE	paclitaxel	21660	1/7/05	Injection	L – Antineoplastic and immunomodulating agents
ACUVUE THERAVISION WITH KETOTIFEN	ketotifen fumarate	22388	2/25/22	Ophthalmic	S – Sensory organs
ADASUVE	loxapine	22549	12/21/12	Inhalation	N – Nervous system
ADHANSIA XR	methylphenidate hydrochloride	212038	2/27/19	Oral	N – Nervous system
ADLARITY	donepezil hydrochloride	212304	3/11/22	Topical	N – Nervous system
ADLYXIN	lixisenatide	208471	7/27/16	Injection	A – Alimentary tract and metabolism
ADMELOG SOLOSTAR	insulin lispro	209196	12/11/17	Injection	A – Alimentary tract and metabolism
ADVAIR DISKUS 125/50	fluticasone-salmeterol	21077	8/24/00	Inhalation	R – Respiratory system
ADVAIR HFA 45	fluticasone-salmeterol	21254	6/8/06	Inhalation	R – Respiratory system
AFREZZA	insulin recombinant human	22472	6/27/14	Inhalation	A – Alimentary tract and metabolism
AIRDUO DIGIHALER 55	fluticasone-salmeterol	208799	7/12/19	Inhalation	R – Respiratory system
ALLEGRA D 24 HOUR	fexofenadine hydrochloride; pseudoephedrine hydrochloride	21704	10/19/04	Oral	R – Respiratory system
ALORA	estradiol	20655	12/20/96	Topical	G – Genito urinary system and sex hormones
ALSUMA	sumatriptan succinate	22377	6/29/10	Injection	N – Nervous system
ALVESCO	ciclesonide	21658	1/10/08	Inhalation	R – Respiratory system
AMELUZ	aminolevulinic acid hydrochloride	208081	5/10/16	Topical	L – Antineoplastic and immunomodulating agents
ANDRODERM	testosterone	20489	9/29/95	Topical	G – Genito urinary system and sex hormones
ANNOVERA	ethinyl estradiol; segesterone acetate	209627	8/10/18	Intravaginal	G – Genito urinary system and sex hormones
ANORO ELLIPTA	umeclidinium bromide-vilanterol trifenetate	203975	12/18/13	Inhalation	R – Respiratory system
APIDRA SOLOSTAR	insulin glulisine recombinant	21629	4/16/04	Injection	A – Alimentary tract and metabolism
ARCAPTA NEOHALER	indacaterol	22383	7/1/11	Inhalation	R – Respiratory system
ARESTIN	minocycline hydrochloride	50781	2/16/01	Dental	A – Alimentary tract and metabolism
ARIKAYCE KIT	amikacin sulfate	207356	9/28/18	Inhalation	J – Antiinfectives for systemic use
ARMONAIR DIGIHALER	fluticasone	208798	1/27/17	Inhalation	R – Respiratory system
ARNUITY ELLIPTA 100	fluticasone furoate	205625	8/20/14	Inhalation	R – Respiratory system
ASMANEX TWISTHALER 220	mometasone	21067	3/30/05	Inhalation	R – Respiratory system
ASPRUZYO SPRINKLE	ranolazine	216018	2/28/22	Oral	C – Cardiovascular system
ATROVENT HFA	ipratropium bromide	21527	11/27/04	Inhalation	R – Respiratory system
AUVI-Q	epinephrine	201739	8/10/12	Injection	C – Cardiovascular system
AVAGARD	alcohol; chlorhexidine gluconate	21074	6/7/01	Topical	D – Dermatologicals
AXIRON	testosterone	22504	11/23/10	Topical	G – Genito urinary system and sex hormones
BAQSIMI	glucagon	210134	7/24/19	Intranasal	H – Systemic hormonal preparations, excl. sex hormones and insulins
BELBUCA	buprenorphine hydrochloride	207932	10/23/15	Oral	N – Nervous system
BREO ELLIPTA 100	fluticasone-vilanterol	204275	5/10/13	Inhalation	R – Respiratory system
BREZTRI AEROSPHERE	budesonide-formoterol-glycopyrrolate	212122	7/23/20	Inhalation	R – Respiratory system
BUNAVAIL	buprenorphine hydrochloride; naloxone hydrochloride	205637	6/6/14	Oral	N – Nervous system
BUSPAR	buspirone hydrochloride	18731	9/29/86	Oral	N – Nervous system
BUTRANS	buprenorphine	21306	6/30/10	Topical	N – Nervous system

© 2025 Teng TW, et al. JAMA Health Forum.

BYDUREON	exenatide synthetic	22200	1/27/12	Injection	A – Alimentary tract and metabolism
BYDUREON BCISE	exenatide synthetic	209210	10/20/17	Injection	A – Alimentary tract and metabolism
CAMCEVI KIT	leuprolide mesylate	211488	5/25/21	Injection	L – Antineoplastic and immunomodulating agents
CANASA	mesalamine	21252	1/5/01	Rectal	A – Alimentary tract and metabolism
CARAC	fluorouracil	20985	10/27/00	Topical	L – Antineoplastic and immunomodulating agents
CARBATROL	carbamazepine	20712	9/30/97	Oral	N – Nervous system
CARDIZEM LA	diltiazem hydrochloride	21392	2/6/03	Oral	C – Cardiovascular system
CARDURA XL	doxazosin mesylate	21269	2/22/05	Oral	C – Cardiovascular system
CAVERJECT IMPULSE	alprostadil	21212	6/11/02	Injection	G – Genito urinary system and sex hormones
CERVIDIL	dinoprostone	20411	3/30/95	Intravaginal	G – Genito urinary system and sex hormones
CHLORAPREP ONE-STEP FREPP	chlorhexidine gluconate; isopropyl alcohol	20832	7/14/00	Topical	D – Dermatologicals
CHLORAPREP ONE-STEP SEPP	chlorhexidine gluconate; isopropyl alcohol	21555	10/7/02	Topical	D – Dermatologicals
CHLORASCRUB MAXI SWABSTICK	chlorhexidine gluconate; isopropyl alcohol	21524	6/3/05	Topical	D – Dermatologicals
CHLORHEXIDINE GLUCONATE	chlorhexidine gluconate	21669	4/25/05	Topical	D – Dermatologicals
CLIMARA	estradiol	20375	12/22/94	Topical	G – Genito urinary system and sex hormones
CLIMARA PRO	estradiol; levonorgestrel	21258	11/21/03	Topical	G – Genito urinary system and sex hormones
CLINDESSE	clindamycin phosphate	50793	11/30/04	Intravaginal	G – Genito urinary system and sex hormones
COMBIPATCH	estradiol	20870	8/7/98	Topical	G – Genito urinary system and sex hormones
COMBIVENT RESPIMAT	albuterol-ipratopium	21747	10/7/11	Inhalation	R – Respiratory system
CONCERTA	methylphenidate hydrochloride	21121	8/1/00	Oral	N – Nervous system
COREG CR	carvedilol phosphate	22012	10/20/06	Oral	C – Cardiovascular system
COVERA-HS	verapamil hydrochloride	20552	2/26/96	Oral	C – Cardiovascular system
CRINONE	progesterone	20701	7/31/97	Intravaginal	G – Genito urinary system and sex hormones
DAYTRANA	methylphenidate	21514	4/6/06	Topical	N – Nervous system
DEPOCYT	cytarabine	21041	4/1/99	Injection	L – Antineoplastic and immunomodulating agents
DEPODUR	morphine sulfate	21671	5/18/04	Injection	N – Nervous system
DEXILANT / KAPIDEX	dexlansoprazole	22287	1/30/09	Oral	A – Alimentary tract and metabolism
DEXTENZA	dexamethasone	208742	11/30/18	Ophthalmic	S – Sensory organs
DEXYCU KIT	dexamethasone	208912	2/9/18	Ophthalmic	S – Sensory organs
DILACOR XR	diltiazem hydrochloride	20092	5/29/92	Oral	C – Cardiovascular system
DITROPAN XL	oxybutynin chloride	20897	12/16/98	Oral	G – Genito urinary system and sex hormones
DSUVIA	sufentanil citrate	209128	11/2/18	Oral	N – Nervous system
DUAKLIR PRESSAIR	aclidinium bromide-formoterol fumerate	210595	3/29/19	Inhalation	R – Respiratory system
DUODOTE	atropine; pralidoxime chloride	21983	9/28/06	Injection	V – Various
DURAGESIC	fentanyl	19813	8/7/90	Topical	N – Nervous system
DURYSTA	bimatoprost	211911	3/4/20	Ophthalmic	S – Sensory organs
DYNACIRC CR	isradipine	20336	6/1/94	Oral	C – Cardiovascular system
EFIDAC 24 CHLORPHENIRAMINE MALEAT	chlorpheniramine maleate	19746	11/18/94	Oral	R – Respiratory system
EFIDAC 24 PSEUDOEPHEDRINE HCL	pseudoephedrine hydrochloride	20021	12/15/92	Oral	R – Respiratory system
EFIDAC 24 PSEUDOEPHEDRINE HCL/BRO	brompheniramine maleate	19672	3/29/96	Oral	R – Respiratory system
ELIGARD	leuprolide acetate	21731	12/14/04	Injection	L – Antineoplastic and immunomodulating agents
ELIGARD KIT	leuprolide acetate	21343	1/23/02	Injection	L – Antineoplastic and immunomodulating agents

© 2025 Teng TW, et al. JAMA Health Forum.

ELIGARD KIT	leuprolide acetate	21379	7/24/02	Injection	L – Antineoplastic and immunomodulating agents
ELIGARD KIT	leuprolide acetate	21488	2/13/03	Injection	L – Antineoplastic and immunomodulating agents
EMEND	aprepitant	21549	3/26/03	Oral	A – Alimentary tract and metabolism
EMSAM	selegiline	21336	2/27/06	Topical	N – Nervous system
ENSTILAR	betamethasone dipropionate; calcipotriene	207589	10/16/15	Topical	D – Dermatologicals
EPIPEN/EPIPEN JR.	epinephrine	19430	12/22/87	Injection	C – Cardiovascular system
EQUETRO	carbamazepine	21710	12/10/04	Oral	N – Nervous system
ESCLIM	estradiol	20847	8/4/98	Topical	G – Genito urinary system and sex hormones
ESKATA	hydrogen peroxide	209305	12/14/17	Topical	D – Dermatologicals
ESTRADERM	estradiol	19081	9/10/86	Topical	G – Genito urinary system and sex hormones
ESTRADIOL	estradiol	20538	7/31/96	Topical	G – Genito urinary system and sex hormones
ESTRASORB	estradiol hemihydrate	21371	10/9/03	Topical	G – Genito urinary system and sex hormones
ETHYOL	amifostine	20221	12/8/95	Injection	V – Various
EVAMIST	estradiol	22014	7/27/07	Topical	G – Genito urinary system and sex hormones
EVZIO	naloxone hydrochloride	205787	4/3/14	Injection	V – Various
EVZIO	naloxone hydrochloride	209862	10/19/16	Injection	V – Various
EXPAREL	bupivacaine	22496	10/28/11	Injection	N – Nervous system
EXSERVAN	riluzole	212640	11/22/19	Oral	N – Nervous system
EXUBERA	insulin recombinant human	21868	1/27/07	Inhalation	A – Alimentary tract and metabolism
EYSUVIS	loteprednol etabonate	210933	10/26/20	Ophthalmic	S – Sensory organs
FARXIGA	dapagliflozin propanediol	202293	1/8/14	Oral	A – Alimentary tract and metabolism
FAZACLO ODT	clozapine	21590	2/10/04	Oral	N – Nervous system
FEMPATCH	estradiol	20417	12/3/96	Topical	G – Genito urinary system and sex hormones
FEMRING	estradiol acetate	21367	3/20/03	Intravaginal	G – Genito urinary system and sex hormones
FENSOLVI KIT	leuprolide acetate	213150	5/1/20	Injection	L – Antineoplastic and immunomodulating agents
FIASP FLEXTOUCH	insulin aspart	208751	9/29/17	Injection	A – Alimentary tract and metabolism
FINACEA	azelaic acid	207071	7/29/15	Topical	D – Dermatologicals
FINTEPLA	fenfluramine hydrochloride	212102	6/25/20	Oral	N – Nervous system
FLO-PRED	prednisolone acetate	22067	1/17/08	Oral	H – Systemic hormonal preparations, excl. sex hormones and insulins
FLOVENT DISKUS 50	fluticasone propionate	20833	9/29/00	Inhalation	R – Respiratory system
FLOVENT HFA 220	fluticasone propionate	21433	5/14/04	Inhalation	R – Respiratory system
FLUTICASONE PROPIONATE	xhance	209022	9/18/17	Intranasal	R – Respiratory system
FLUXID	famotidine	21712	9/24/04	Oral	A – Alimentary tract and metabolism
FORADIL AEROLIZER	formoterol fumarate	20831	2/16/01	Inhalation	R – Respiratory system
FORADIL CENTRIHALER	formoterol fumarate	21592	12/15/06	Inhalation	R – Respiratory system
FORTEO	teriparatide recombinant human	21318	11/26/02	Injection	H – Systemic hormonal preparations, excl. sex hormones and insulins
GELNIQUE	oxybutynin chloride	22204	1/27/09	Topical	G – Genito urinary system and sex hormones
GENOSYL	nitric oxide	202860	12/20/19	Inhalation	R – Respiratory system
GENOTROPIN PRESERVATIVE FREE	somatropin recombinant	20280	8/24/95	Injection	H – Systemic hormonal preparations, excl. sex hormones and insulins
GLIADEL	carmustine	20637	9/23/96	Intracranial	L – Antineoplastic and immunomodulating agents
GLUCOTROL XL	glipizide	20329	4/26/94	Oral	A – Alimentary tract and metabolism
GLYNASE	glyburide	20051	3/4/92	Oral	A – Alimentary tract and metabolism
GYNAZOLE-1	butoconazole nitrate	19881	2/7/97	Intravaginal	G – Genito urinary system and sex hormones
HABITROL	nicotine	20076	11/27/91	Topical	N – Nervous system
HEPZATO	melphalan hydrochloride	201848	8/14/23	Injection	L – Antineoplastic and immunomodulating agents
HUMALOG KWIKPEN	insulin lispro recombinant	20563	6/14/96	Injection	A – Alimentary tract and metabolism
HUMALOG KWIKPEN	insulin lispro recombinant	205747	5/26/15	Injection	A – Alimentary tract and metabolism
HUMALOG MIX 50/50 KWIKPEN	insulin lispro protamine recombinant; insulin lispro recombinant	21018	12/22/99	Injection	A – Alimentary tract and metabolism

© 2025 Teng TW, et al. JAMA Health Forum.

HUMALOG MIX 75/25 KWIKPEN	insulin lispro protamine recombinant; insulin lispro recombinant	21017	12/22/99	Injection	A – Alimentary tract and metabolism
HUMULIN 70/30 PEN	insulin recombinant human; insulin susp isophane recombinant human	19717	4/25/89	Injection	A – Alimentary tract and metabolism
IDOSE TR	travoprost	218010	12/13/23	Ophthalmic	S – Sensory organs
IGALMI	dexmedetomidine hydrochloride	215390	4/5/22	Oral	N – Nervous system
ILUVIEN	fluocinolone acetonide	201923	9/26/14	Ophthalmic	S – Sensory organs
IMITREX	sumatriptan	20626	8/26/97	Intranasal	N – Nervous system
IMPLANON	etonogestrel	21529	7/17/06	Other	G – Genito urinary system and sex hormones
IMVEXXY	estradiol	208564	5/29/18	Intravaginal	G – Genito urinary system and sex hormones
INBRIJA	levodopa	209184	12/21/18	Inhalation	N – Nervous system
INCRUSE ELLIPTA	umeclidinium bromide	205382	4/30/14	Inhalation	R – Respiratory system
INOMAX	nitric oxide	20845	12/23/99	Inhalation	R – Respiratory system
INTELENCE	etravirine	22187	1/18/08	Oral	J – Antiinfectives for systemic use
INTRAROSA	prasterone	208470	11/16/16	Intravaginal	G – Genito urinary system and sex hormones
INVEGA SUSTENNA	paliperidone palmitate	22264	7/31/09	Injection	N – Nervous system
INVELTYS	loteprednol etabonate	210565	8/22/18	Ophthalmic	S – Sensory organs
IONSYS	fentanyl hydrochloride	21338	5/22/06	Topical	N – Nervous system
JELMYTO	mitomycin	211728	4/15/20	Ophthalmic	L – Antineoplastic and immunomodulating agents
KEMSTRO	baclofen	21589	10/30/03	Oral	M – Musculo-skeletal system
KYLEENA	levonorgestrel	208224	9/16/16	Intrauterine	G – Genito urinary system and sex hormones
KYNMOBI	apomorphine hydrochloride	210875	5/21/20	Oral	N – Nervous system
LANTUS	insulin glargine recombinant	21081	4/20/00	Injection	A – Alimentary tract and metabolism
LAZANDA	fentanyl citrate	22569	6/30/11	Intranasal	N – Nervous system
LEVEMIR FLEXTOUCH	insulin detemir recombinant	21536	6/16/05	Injection	A – Alimentary tract and metabolism
LEVULAN	aminolevulinic acid hydrochloride	20965	12/3/99	Topical	L – Antineoplastic and immunomodulating agents
LIDODERM	lidocaine	20612	3/19/99	Topical	N – Nervous system
LIDOSITE TOPICAL SYSTEM KIT	epinephrine; lidocaine hydrochloride	21504	5/6/04	Topical	N – Nervous system
LILETTA	levonorgestrel	206229	2/26/15	Intrauterine	G – Genito urinary system and sex hormones
LONHALA MAGNAIR KIT	glycopyrrolate	208437	12/5/17	Inhalation	D – Dermatologicals
LOPRESSOR	metoprolol fumarate	19786	12/27/89	Oral	C – Cardiovascular system
LUPANETA PACK	leuprolide acetate; norethindrone acetate	203696	12/14/12	Other	L – Antineoplastic and immunomodulating agents
LUPRON DEPOT	leuprolide acetate	19732	1/26/89	Injection	L – Antineoplastic and immunomodulating agents
LUPRON DEPOT	leuprolide acetate	20517	12/22/95	Injection	L – Antineoplastic and immunomodulating agents
LUPRON DEPOT	leuprolide acetate	20011	10/22/90	Injection	L – Antineoplastic and immunomodulating agents
LUPRON DEPOT-3	leuprolide acetate	20708	3/7/97	Injection	L – Antineoplastic and immunomodulating agents
LUPRON/LUPRON DEPOT	leuprolide acetate	20263	4/16/93	Injection	L – Antineoplastic and immunomodulating agents
LUTRATE DEPOT KIT	leuprolide acetate	205054	8/28/18	Injection	L – Antineoplastic and immunomodulating agents
MACROBID	nitrofurantoin	20064	12/24/91	Oral	J – Antiinfectives for systemic use
MACUGEN	pegaptanib sodium	21756	9/17/04	Ophthalmic	S – Sensory organs
MAKENA (AUTOINJECTOR)	hydroxyprogesterone caproate	21945	2/3/11	Injection	G – Genito urinary system and sex hormones
MAXAIR AUTOHALER	pirbuterol acetate	20014	11/30/92	Inhalation	R – Respiratory system
MEGACE ES	megestrol acetate	21778	7/5/05	Oral	L – Antineoplastic and immunomodulating agents
MENOSTAR	estradiol	21674	6/8/04	Topical	G – Genito urinary system and sex hormones
MIGRANAL	dihydroergotamine mesylate	20148	12/8/97	Intranasal	N – Nervous system

© 2025 Teng TW, et al. JAMA Health Forum.

MILPROSA	progesterone	201110	4/29/20	Intravaginal	G – Genito urinary system and sex hormones
MINIPRESS XL	prazosin hydrochloride	19775	1/29/92	Oral	C – Cardiovascular system
MINIVELLE	estradiol	203752	10/29/12	Topical	G – Genito urinary system and sex hormones
MIRENA	levonorgestrel	21225	12/6/00	Intrauterine	G – Genito urinary system and sex hormones
MITOSOL	mitomycin	22572	2/7/12	Topical	L – Antineoplastic and immunomodulating agents
MORPHINE SULFATE	morphine sulfate	204223	10/30/13	Injection	N – Nervous system
MOUNJARO	tirzepatide	215866	5/13/22	Injection	A – Alimentary tract and metabolism
MUSE	alprostadil	20700	11/19/96	Urethral	G – Genito urinary system and sex hormones
NALOXONE HYDROCHLORIDE (AUTOINJECTOR)	naloxone hydrochloride	215457	2/28/22	Injection	V – Various
NARCAN	naloxone hydrochloride	208411	11/18/15	Intranasal	V – Various
NAROPIN	ropivacaine hydrochloride	20533	9/24/96	Injection	N – Nervous system
NASCOBAL	cyanocobalamin	21642	1/31/05	Intranasal	B – Blood and blood forming organs
NEUPRO	rotigotine	21829	5/9/07	Topical	N – Nervous system
NICODERM CQ	nicotine	20165	11/7/91	Topical	N – Nervous system
NICOTROL	nicotine	20714	5/2/97	Oral	N – Nervous system
NICOTROL	nicotine	20536	7/3/96	Topical	N – Nervous system
NIRAVAM	alprazolam	21726	1/19/05	Oral	N – Nervous system
NOCTIVA	desmopressin acetate	201656	3/3/17	Intranasal	H – Systemic hormonal preparations, excl. sex hormones and insulins
NORDITROPIN NORDIFLEX	somatropin recombinant	21148	6/20/00	Injection	H – Systemic hormonal preparations, excl. sex hormones and insulins
NOVOLOG MIX 70/30 FLEXPEN	insulin aspart protamine recombinant; insulin aspart recombinant	21172	11/1/01	Injection	A – Alimentary tract and metabolism
NOVOLOG PENFILL	insulin aspart recombinant	20986	6/7/00	Injection	A – Alimentary tract and metabolism
NUTROPIN DEPOT	somatropin recombinant	21075	12/22/99	Injection	H – Systemic hormonal preparations, excl. sex hormones and insulins
NUVARING	ethinyl estradiol; etonogestrel	21187	10/3/01	Intravaginal	G – Genito urinary system and sex hormones
NYMALIZE	nimodipine	203340	5/10/13	Oral	C – Cardiovascular system
OLUX E	clobetasol propionate	22013	1/12/07	Topical	D – Dermatologicals
ONIVYDE	irinotecan hydrochloride	207793	10/22/15	Injection	L – Antineoplastic and immunomodulating agents
ONPATTRO	patisiran sodium	210922	8/10/18	Injection	N – Nervous system
ONSOLIS	fentanyl citrate	22266	7/16/09	Oral	N – Nervous system
ONZETRA XSAIL	sumatriptan succinate	206099	1/27/16	Intranasal	N – Nervous system
OPANA ER	oxymorphone hydrochloride	21610	6/22/06	Oral	N – Nervous system
ORAPRED ODT	prednisolone sodium phosphate	21959	6/1/06	Oral	H – Systemic hormonal preparations, excl. sex hormones and insulins
ORTHO EVRA	ethinyl estradiol; norelgestromin	21180	11/20/01	Topical	G – Genito urinary system and sex hormones
OSMOLEX ER	amantadine hydrochloride	209410	2/16/18	Oral	N – Nervous system
OTIPRIO	ciprofloxacin	207986	12/10/15	Otic	S – Sensory organs
OTREXUP	methotrexate	204824	10/11/13	Injection	L – Antineoplastic and immunomodulating agents
OXYTROL	oxybutynin	21351	2/26/03	Topical	G – Genito urinary system and sex hormones
OXYTROL FOR WOMEN	oxybutynin	202211	1/25/13	Topical	G – Genito urinary system and sex hormones
OZEMPIC	semaglutide	209637	12/5/17	Injection	A – Alimentary tract and metabolism
OZURDEX	dexamethasone	22315	6/17/09	Ophthalmic	S – Sensory organs
PAXIL CR	paroxetine hydrochloride	20936	2/16/99	Oral	N – Nervous system
PEPCID AC	famotidine	20902	8/5/99	Oral	A – Alimentary tract and metabolism
POMALYST	pomalidomide	204026	2/8/13	Oral	L – Antineoplastic and immunomodulating agents
PRADAXA	dabigatran etexilate mesylate	22512	10/19/10	Oral	B – Blood and blood forming organs
PROAIR HFA	albuterol sulfate	21457	10/29/04	Inhalation	R – Respiratory system
PROAIR RESPICLICK	albuterol sulfate	205636	3/31/15	Inhalation	R – Respiratory system
PROCANBID	procainamide hydrochloride	20545	1/31/96	Oral	C – Cardiovascular system

© 2025 Teng TW, et al. JAMA Health Forum.

PROCARDIA XL	nifedipine	19684	9/6/89	Oral	C – Cardiovascular system
PROVENTIL HFA	albuterol	20503	8/15/96	Inhalation	R – Respiratory system
PULMICORT 160	budesonide	20441	6/24/97	Inhalation	R – Respiratory system
PULMICORT FLEXHALER 80	budesonide	21949	7/12/06	Inhalation	R – Respiratory system
PULMICORT RESPULES	budesonide	20929	8/8/00	Inhalation	R – Respiratory system
QBREXZA	glycopyrronium tosylate	210361	6/28/18	Topical	D – Dermatologicals
QNASL	beclomethasone dipropionate	202813	3/23/12	Intranasal	R – Respiratory system
QUTENZA	capsaicin	22395	11/16/09	Topical	N – Nervous system
QVAR 80	beclomethasone dipropionate	20911	9/15/00	Inhalation	R – Respiratory system
QVAR REDIHALER	beclomethasone dipropionate	207921	8/3/17	Inhalation	R – Respiratory system
RAPAMUNE	sirolimus	21110	8/25/00	Oral	L – Antineoplastic and immunomodulating agents
REGLAN ODT	metoclopramide hydrochloride	21793	6/10/05	Oral	A – Alimentary tract and metabolism
RELENZA	zanamivir	21036	7/26/99	Inhalation	J – Antiinfectives for systemic use
RENOVA	tretinoin	19963	12/29/95	Topical	D – Dermatologicals
REQUIP XL	ropinirole hydrochloride	22008	6/13/08	Oral	N – Nervous system
RESTASIS MULTIDOSE	cyclosporine	50790	12/23/02	Ophthalmic	S – Sensory organs
RETIN-A MICRO	tretinoin	20475	2/7/97	Topical	D – Dermatologicals
RETISERT	fluocinolone acetonide	21737	4/8/05	Ophthalmic	S – Sensory organs
REVLIMID	lenalidomide	21880	12/27/05	Oral	L – Antineoplastic and immunomodulating agents
RISPERDAL CONSTA	risperidone	21346	10/29/03	Injection	N – Nervous system
RIZAFILM	rizatriptan benzoate	205394	4/14/23	Oral	N – Nervous system
ROWASA	mesalamine	19618	12/24/87	Rectal	A – Alimentary tract and metabolism
RYALTRIS	mometasone furoate; olopatadine hydrochloride	211746	1/13/22	Intranasal	R – Respiratory system
RYKINDO	risperidone	212849	1/13/23	Injection	N – Nervous system
RYTHMOL SR	propafenone hydrochloride	21416	9/4/03	Oral	C – Cardiovascular system
RYZODEG 70/30	insulin aspart; insulin degludec	203313	9/25/15	Injection	A – Alimentary tract and metabolism
SANCUSO	granisetron	22198	9/12/08	Topical	A – Alimentary tract and metabolism
SANDOSTATIN LAR	octreotide acetate	21008	11/25/98	Injection	H – Systemic hormonal preparations, excl. sex hormones and insulins
SAXENDA	liraglutide recombinant	206321	12/23/14	Injection	A – Alimentary tract and metabolism
SECUADO	asenapine	212268	10/11/19	Topical	N – Nervous system
SEEBRI NEOHALER	glycopyrrolate	207923	10/29/15	Inhalation	D – Dermatologicals
SEREVENT DISKUS	salmeterol xinafoate	20692	9/19/97	Inhalation	R – Respiratory system
SIGNIFOR LAR	pasireotide pamoate	203255	12/15/14	Injection	H – Systemic hormonal preparations, excl. sex hormones and insulins
SINUVA	mometasone furoate	209310	12/8/17	Other	D – Dermatologicals
SKYLA	levonorgestrel	203159	1/9/13	Intrauterine	G – Genito urinary system and sex hormones
SOLIQUA 100/33	insulin glargine/lixisenatide	208673	11/21/16	Injection	A – Alimentary tract and metabolism
SPIRIVA HANDIHALER	tiotropium bromide monohydrate	21395	1/30/04	Inhalation	R – Respiratory system
SPIRIVA RESPIMAT 25	tiotropium bromide	21936	9/24/14	Inhalation	R – Respiratory system
SPORANOX	itraconazole	20083	9/11/92	Oral	J – Antiinfectives for systemic use
STIOLTO RESPIMAT	olodaterol hydrochloride-tiotropium bromide	206756	5/21/15	Inhalation	R – Respiratory system
STRIANT	testosterone	21543	6/19/03	Oral	G – Genito urinary system and sex hormones
STRIVERDI RESPIMAT	olodaterol	203108	7/31/14	Inhalation	R – Respiratory system
SUBLOCADE	buprenorphine	209819	11/30/17	Injection	N – Nervous system
SUBOXONE	buprenorphine hydrochloride; naloxone hydrochloride	22410	8/30/10	Oral	N – Nervous system
SULAR	nisoldipine	20356	2/2/95	Oral	C – Cardiovascular system
SUMAVEL DOSEPRO	sumatriptan succinate	22239	7/15/09	Injection	N – Nervous system
SUPPRELIN LA	histrelin acetate	22058	5/3/07	Injection	L – Antineoplastic and immunomodulating agents

© 2025 Teng TW, et al. JAMA Health Forum.

SUPRANE	desflurane	20118	9/18/92	Inhalation	N – Nervous system
SUSTOL	granisetron	22445	8/9/16	Injection	A – Alimentary tract and metabolism
SYMBICORT	budesonide-formoterol fumarate	21929	7/21/06	Inhalation	R – Respiratory system
SYMJEPI	epinephrine	207534	6/15/17	Injection	C – Cardiovascular system
SYMPAZAN	clobazam	210833	11/1/18	Oral	N – Nervous system
SYMTUZA	cobicistat; darunavir ethanolate; emtricitabine; tenofovir alafenamide fumarate	210455	7/17/18	Oral	J – Antiinfectives for systemic use
SYNERA	lidocaine; tetracaine	21623	6/23/05	Topical	N – Nervous system
TECZEM	diltiazem malate; enalapril maleate	20507	10/4/96	Oral	C – Cardiovascular system
TEGRETOL-XR	carbamazepine	20234	3/25/96	Oral	N – Nervous system
TESTODERM	testosterone	19762	10/12/93	Topical	G – Genito urinary system and sex hormones
TESTODERM TTS	testosterone	20791	12/18/97	Topical	G – Genito urinary system and sex hormones
THALOMID	thalidomide	20785	7/16/98	Oral	L – Antineoplastic and immunomodulating agents
TIAMATE	diltiazem malate	20506	10/4/96	Oral	C – Cardiovascular system
TOBI PODHALER	tobramycin	201688	3/22/13	Inhalation	J – Antiinfectives for systemic use
TOPROL-XL	metoprolol succinate	19962	1/10/92	Oral	C – Cardiovascular system
TOUJEO SOLOSTAR	insulin glargine recombinant	206538	2/25/15	Injection	A – Alimentary tract and metabolism
TRELEGY ELLIPTA	fluticasone-umeclidinium-vilanterol	209482	9/18/17	Inhalation	R – Respiratory system
TRELSTAR	triptorelin pamoate	21288	6/29/01	Injection	L – Antineoplastic and immunomodulating agents
TRELSTAR DEPOT	triptorelin pamoate	20715	6/15/00	Injection	L – Antineoplastic and immunomodulating agents
TRESIBA	insulin degludec	203314	9/25/15	Injection	A – Alimentary tract and metabolism
TRICOR	fenofibrate	21656	11/5/04	Oral	C – Cardiovascular system
TRUDHESA	dihydroergotamine mesylate	213436	9/2/21	Intranasal	N – Nervous system
TUDORZA PRESSAIR	aclidinium bromide	202450	7/23/12	Inhalation	R – Respiratory system
TWINJECT 0.3	epinephrine	20800	5/30/03	Injection	C – Cardiovascular system
TWIRLA	ethinyl estradiol; levonorgestrel	204017	2/14/20	Topical	G – Genito urinary system and sex hormones
TWYNEO	benzoyl peroxide; tretinoin	214902	7/26/21	Topical	D – Dermatologicals
TYVASO	treprostinil	22387	7/30/09	Inhalation	B – Blood and blood forming organs
TYVASO DPI	treprostinil	214324	5/23/22	Inhalation	B – Blood and blood forming organs
ULTANE	sevoflurane	20478	6/7/95	Inhalation	N – Nervous system
UTIBRON NEOHALER	indacaterol-glycopyrrolate	207930	10/29/15	Inhalation	R – Respiratory system
UZEDY	risperidone	213586	4/28/23	Injection	N – Nervous system
VAGIFEM	estradiol	20908	3/26/99	Intravaginal	G – Genito urinary system and sex hormones
VANTAS	histrelin acetate	21732	10/12/04	Injection	L – Antineoplastic and immunomodulating agents
VARITHENA	polidocanol	205098	11/25/13	Injection	C – Cardiovascular system
VENTOLIN HFA	albuterol	20983	4/19/01	Inhalation	R – Respiratory system
VENTOLIN ROTACAPS	albuterol	19489	5/4/88	Inhalation	R – Respiratory system
VERAMYST	fluticasone furoate	22051	4/27/07	Intranasal	R – Respiratory system
VERDESO	desonide	21978	9/19/06	Topical	D – Dermatologicals
VIADUR	leuprolide acetate	21088	3/3/00	Other	L – Antineoplastic and immunomodulating agents
VICTOZA	liraglutide recombinant	22341	1/25/10	Injection	A – Alimentary tract and metabolism
VITRASERT	ganciclovir	20569	3/4/96	Other	S – Sensory organs
VIVITROL	naltrexone	21897	4/13/06	Injection	N – Nervous system
VOLMAX	albuterol sulfate	19604	12/23/92	Oral	R – Respiratory system
VYXEOS	cytarabine; daunorubicin	209401	8/3/17	Inhalation	L – Antineoplastic and immunomodulating agents
XELSTRYM	dextroamphetamine	215401	3/22/22	Topical	N – Nervous system
XIPERE	triamcinolone acetonide	211950	10/22/21	Injection	S – Sensory organs
XOPENEX HFA	levalbuterol	21730	3/11/05	Inhalation	R – Respiratory system

© 2025 Teng TW, et al. JAMA Health Forum.

XTAMPZA ER	oxycodone	208090	4/26/16	Oral	N – Nervous system
XULTOPHY 100/3.6	insulin degludec/liraglutide	208583	11/21/16	Injection	A – Alimentary tract and metabolism
XYOSTED (AUTOINJECTOR)	testosterone enanthate	209863	9/28/18	Injection	G – Genito urinary system and sex hormones
YCANTH	cantharidin	212905	7/21/23	Topical	N/A
YUTIQ	fluocinolone acetonide	210331	10/12/18	Ophthalmic	S – Sensory organs
ZECUITY	sumatriptan succinate	202278	1/17/13	Other	N – Nervous system
ZEPBOUND	tirzepatide	217806	11/8/23	Injection	A – Alimentary tract and metabolism
ZETONNA	ciclesonide	202129	1/20/12	Intranasal	R – Respiratory system
ZIMHI	naloxone hydrochloride	212854	10/15/21	Injection	V – Various
ZINGO	lidocaine hydrochloride	22114	8/16/07	Topical	N – Nervous system
ZOLADEX	goserelin acetate	19726	12/29/89	Other	L – Antineoplastic and immunomodulating agents
ZOLADEX	goserelin acetate	20578	1/11/96	Other	L – Antineoplastic and immunomodulating agents
ZTALMY	ganaxolone	215904	3/18/22	Oral	N – Nervous system
ZTLIDO	lidocaine	207962	2/28/18	Topical	N – Nervous system
ZUPLENZ	ondansetron	22524	7/2/10	Oral	A – Alimentary tract and metabolism
ZURAGARD	isopropyl alcohol	210872	4/26/19	Topical	D – Dermatologicals
ZYFLO CR	zileuton	22052	5/30/07	Oral	N/A
ZYNRELEF KIT	bupivacaine; meloxicam	211988	5/12/21	Periarticular	N – Nervous system

FDA: Food and Drug Administration; NDA: New Drug Application.

© 2025 Teng TW, et al. JAMA Health Forum.

eTable 3: Patents listed on multiple drug-device combinations, 1986-2023

Patent number	Patent category	Number of products on which the patent is listed	Products on which the patent is listed
7762994	Tertiary	11	Levemir Flextouch (insulin detemir recombinant)
			Saxenda (liraglutide recombinant)
			Xultophy 100/3.6 (insulin degludec/liraglutide)
			Fiasp Flextouch (insulin aspart)
			Ryzodeg 70/30 (insulin aspart; insulin degludec)
			Novolog Penfill (insulin aspart recombinant)
			Ozempic (semaglutide)
			Novolog Mix 70/30 Flexpen (insulin aspart protamine recombinant; insulin aspart recombinant)
			Tresiba (insulin degludec)
			Victoza (liraglutide recombinant)
			Norditropin Nordiflex (somatropin recombinant)
8579869	Tertiary	11	Levemir Flextouch (insulin detemir recombinant)
			Saxenda (liraglutide recombinant)
			Xultophy 100/3.6 (insulin degludec/liraglutide)
			Fiasp Flextouch (insulin aspart)
			Ryzodeg 70/30 (insulin aspart; insulin degludec)
			Novolog Penfill (insulin aspart recombinant)
			Ozempic (semaglutide)
			Novolog Mix 70/30 Flexpen (insulin aspart protamine recombinant; insulin aspart recombinant)
			Tresiba (insulin degludec)
			Victoza (liraglutide recombinant)
			Norditropin Nordiflex (somatropin recombinant)
6899699	Tertiary	9	Levemir Flextouch (insulin detemir recombinant)
			Saxenda (liraglutide recombinant)
			Xultophy 100/3.6 (insulin degludec/liraglutide)
			Fiasp Flextouch (insulin aspart)
			Ryzodeg 70/30 (insulin aspart; insulin degludec)
			Tresiba (insulin degludec)
			Ozempic (semaglutide)
			Novolog Penfill (insulin aspart recombinant)
			Norditropin Nordiflex (somatropin recombinant)
8672898	Tertiary	9	Levemir Flextouch (insulin detemir recombinant)
			Saxenda (liraglutide recombinant)
			Xultophy 100/3.6 (insulin degludec/liraglutide)
			Fiasp Flextouch (insulin aspart)
			Ryzodeg 70/30 (insulin aspart; insulin degludec)
			Tresiba (insulin degludec)
			Ozempic (semaglutide)
			Novolog Penfill (insulin aspart recombinant)
			Norditropin Nordiflex (somatropin recombinant)
8684969	Tertiary	9	Levemir Flextouch (insulin detemir recombinant)
			Saxenda (liraglutide recombinant)
			Xultophy 100/3.6 (insulin degludec/liraglutide)
			Fiasp Flextouch (insulin aspart)

© 2025 Teng TW, et al. JAMA Health Forum.

			Ryzodeg 70/30 (insulin aspart; insulin degludec)
			Tresiba (insulin degludec)
			Ozempic (semaglutide)
			Novolog Penfill (insulin aspart recombinant)
			Norditropin Nordiflex (somatropin recombinant)
8920383	Tertiary	9	Levemir Flextouch (insulin detemir recombinant)
			Saxenda (liraglutide recombinant)
			Xultophy 100/3.6 (insulin degludec/liraglutide)
			Fiasp Flextouch (insulin aspart)
			Ryzodeg 70/30 (insulin aspart; insulin degludec)
			Tresiba (insulin degludec)
			Ozempic (semaglutide)
			Novolog Penfill (insulin aspart recombinant)
			Norditropin Nordiflex (somatropin recombinant)
9108002	Tertiary	9	Levemir Flextouch (insulin detemir recombinant)
			Saxenda (liraglutide recombinant)
			Xultophy 100/3.6 (insulin degludec/liraglutide)
			Fiasp Flextouch (insulin aspart)
			Ryzodeg 70/30 (insulin aspart; insulin degludec)
			Tresiba (insulin degludec)
			Ozempic (semaglutide)
			Novolog Penfill (insulin aspart recombinant)
			Norditropin Nordiflex (somatropin recombinant)
9132239	Tertiary	9	Levemir Flextouch (insulin detemir recombinant)
			Saxenda (liraglutide recombinant)
			Xultophy 100/3.6 (insulin degludec/liraglutide)
			Fiasp Flextouch (insulin aspart)
			Ryzodeg 70/30 (insulin aspart; insulin degludec)
			Tresiba (insulin degludec)
			Ozempic (semaglutide)
			Novolog Penfill (insulin aspart recombinant)
			Norditropin Nordiflex (somatropin recombinant)
9457154	Tertiary	9	Levemir Flextouch (insulin detemir recombinant)
			Saxenda (liraglutide recombinant)
			Xultophy 100/3.6 (insulin degludec/liraglutide)
			Fiasp Flextouch (insulin aspart)
			Ryzodeg 70/30 (insulin aspart; insulin degludec)
			Tresiba (insulin degludec)
			Ozempic (semaglutide)
			Novolog Penfill (insulin aspart recombinant)
			Norditropin Nordiflex (somatropin recombinant)
9486588	Tertiary	9	Levemir Flextouch (insulin detemir recombinant)
			Saxenda (liraglutide recombinant)
			Xultophy 100/3.6 (insulin degludec/liraglutide)
			Fiasp Flextouch (insulin aspart)
			Ryzodeg 70/30 (insulin aspart; insulin degludec)
			Tresiba (insulin degludec)
			Ozempic (semaglutide)
			Novolog Penfill (insulin aspart recombinant)
			Norditropin Nordiflex (somatropin recombinant)
9687611	Tertiary	9	Levemir Flextouch (insulin detemir recombinant)
			Saxenda (liraglutide recombinant)
			Xultophy 100/3.6 (insulin degludec/liraglutide)

© 2025 Teng TW, et al. JAMA Health Forum.

			Fiasp Flextouch (insulin aspart)
			Ryzodeg 70/30 (insulin aspart; insulin degludec)
			Tresiba (insulin degludec)
			Ozempic (semaglutide)
			Novolog Penfill (insulin aspart recombinant)
			Norditropin Nordiflex (somatropin recombinant)
9775953	Tertiary	9	Levemir Flextouch (insulin detemir recombinant)
			Saxenda (liraglutide recombinant)
			Xultophy 100/3.6 (insulin degludec/liraglutide)
			Fiasp Flextouch (insulin aspart)
			Ryzodeg 70/30 (insulin aspart; insulin degludec)
			Tresiba (insulin degludec)
			Ozempic (semaglutide)
			Novolog Penfill (insulin aspart recombinant)
			Norditropin Nordiflex (somatropin recombinant)
RE46363	Tertiary	9	Levemir Flextouch (insulin detemir recombinant)
			Saxenda (liraglutide recombinant)
			Xultophy 100/3.6 (insulin degludec/liraglutide)
			Fiasp Flextouch (insulin aspart)
			Ryzodeg 70/30 (insulin aspart; insulin degludec)
			Tresiba (insulin degludec)
			Ozempic (semaglutide)
			Novolog Penfill (insulin aspart recombinant)
			Norditropin Nordiflex (somatropin recombinant)
9616180	Tertiary	9	Levemir Flextouch (insulin detemir recombinant)
			Saxenda (liraglutide recombinant)
			Xultophy 100/3.6 (insulin degludec/liraglutide)
			Fiasp Flextouch (insulin aspart)
			Ryzodeg 70/30 (insulin aspart; insulin degludec)
			Tresiba (insulin degludec)
			Ozempic (semaglutide)
			Novolog Penfill (insulin aspart recombinant)
			Norditropin Nordiflex (somatropin recombinant)
9861757	Tertiary	9	Levemir Flextouch (insulin detemir recombinant)
			Saxenda (liraglutide recombinant)
			Xultophy 100/3.6 (insulin degludec/liraglutide)
			Fiasp Flextouch (insulin aspart)
			Ryzodeg 70/30 (insulin aspart; insulin degludec)
			Tresiba (insulin degludec)
			Ozempic (semaglutide)
			Novolog Penfill (insulin aspart recombinant)
			Norditropin Nordiflex (somatropin recombinant)
10220155	Tertiary	8	Levemir Flextouch (insulin detemir recombinant)
			Saxenda (liraglutide recombinant)
			Xultophy 100/3.6 (insulin degludec/liraglutide)
			Fiasp Flextouch (insulin aspart)
			Ryzodeg 70/30 (insulin aspart; insulin degludec)
			Tresiba (insulin degludec)
			Ozempic (semaglutide)
			Norditropin Nordiflex (somatropin recombinant)
10357616	Tertiary	8	Levemir Flextouch (insulin detemir recombinant)
			Saxenda (liraglutide recombinant)
			Xultophy 100/3.6 (insulin degludec/liraglutide)

© 2025 Teng TW, et al. JAMA Health Forum.

			Fiasp Flextouch (insulin aspart)
			Ryzodeg 70/30 (insulin aspart; insulin degludec)
			Tresiba (insulin degludec)
			Ozempic (semaglutide)
			Norditropin Nordiflex (somatropin recombinant)
10376652	Tertiary	8	Levemir Flextouch (insulin detemir recombinant)
			Saxenda (liraglutide recombinant)
			Xultophy 100/3.6 (insulin degludec/liraglutide)
			Fiasp Flextouch (insulin aspart)
			Ryzodeg 70/30 (insulin aspart; insulin degludec)
			Tresiba (insulin degludec)
			Ozempic (semaglutide)
			Norditropin Nordiflex (somatropin recombinant)
7686786	Tertiary	8	Levemir Flextouch (insulin detemir recombinant)
			Saxenda (liraglutide recombinant)
			Xultophy 100/3.6 (insulin degludec/liraglutide)
			Fiasp Flextouch (insulin aspart)
			Ryzodeg 70/30 (insulin aspart; insulin degludec)
			Tresiba (insulin degludec)
			Ozempic (semaglutide)
			Norditropin Nordiflex (somatropin recombinant)
5873360	Tertiary	7	Breo Ellipta 100 (fluticasone-vilanterol)
			Advair Diskus 125/50 (fluticasone-salmeterol)
			Incruse Ellipta (umeclidinium bromide)
			Anoro Ellipta (umeclidinium bromide-vilanterol trifenetate)
			Flovent Diskus 50 (fluticasone propionate)
			Arnuity Ellipta 100 (fluticasone furoate)
			Serevent Diskus (salmeterol xinafoate)
5605674	Secondary	7	Proair Hfa (albuterol sulfate)
			Alvesco (ciclesonide)
			Proventil Hfa (albuterol)
			Xopenex Hfa (levalbuterol)
			Zetonna (ciclesonide)
			Qvar 80 (beclomethasone dipropionate)
			Qnasl (beclomethasone dipropionate)
6221392	Tertiary	7	Fluxid (famotidine)
			Niravam (alprazolam)
			Reglan Odt (metoclopramide hydrochloride)
			Kemstro (baclofen)
			Carbatrol (carbamazepine)
			Fazaclo Odt (clozapine)
			Orapred Odt (prednisolone sodium phosphate)
10022510	Tertiary	6	Qvar Redihaler (beclomethasone dipropionate)
			Airduo Digihaler 55 (fluticasone-salmeterol)
			Proair Hfa (albuterol sulfate)
			Proair Respiclick (albuterol sulfate)
			Armonair Digihaler (fluticasone)
			Qvar 80 (beclomethasone dipropionate)
10561808	Tertiary	6	Qvar Redihaler (beclomethasone dipropionate)
			Airduo Digihaler 55 (fluticasone-salmeterol)
			Proair Hfa (albuterol sulfate)
			Proair Respiclick (albuterol sulfate)
			Armonair Digihaler (fluticasone)

© 2025 Teng TW, et al. JAMA Health Forum.

			Qvar 80 (beclomethasone dipropionate)
6024981	Secondary	6	Fluxid (famotidine)
			Niravam (alprazolam)
			Reglan Odt (metoclopramide hydrochloride)
			Kemstro (baclofen)
			Fazaclo Odt (clozapine)
			Orapred Odt (prednisolone sodium phosphate)
5695743	Secondary	6	Atrovent Hfa (ipratropium bromide)
			Proair Hfa (albuterol sulfate)
			Alvesco (ciclesonide)
			Proventil Hfa (albuterol)
			Xopenex Hfa (levalbuterol)
			Qvar 80 (beclomethasone dipropionate)
4612008	Tertiary	6	Ditropan Xl (oxybutynin chloride)
			Concerta (methylphenidate hydrochloride)
			Procardia Xl (nifedipine)
			Covera-Hs (verapamil hydrochloride)
			Glucotrol Xl (glipizide)
			Minipress Xl (prazosin hydrochloride)
4783337	Tertiary	6	Ditropan Xl (oxybutynin chloride)
			Concerta (methylphenidate hydrochloride)
			Procardia Xl (nifedipine)
			Dynacirc Cr (isradipine)
			Covera-Hs (verapamil hydrochloride)
			Minipress Xl (prazosin hydrochloride)
7291132	Tertiary	5	Humulin 70/30 Pen (insulin recombinant human; insulin susp isophane recombinant human)
			Humalog Kwikpen (insulin lispro recombinant)
			Humalog Kwikpen (insulin lispro recombinant)
			Humalog Mix 75/25 Kwikpen (insulin lispro protamine recombinant; insulin lispro recombinant)
			Humalog Mix 50/50 Kwikpen (insulin lispro protamine recombinant; insulin lispro recombinant)
11771841	Tertiary	5	Eligard (leuprolide acetate)
			Eligard Kit (leuprolide acetate)
			Eligard Kit (leuprolide acetate)
			Fensolvi Kit (leuprolide acetate)
			Eligard Kit (leuprolide acetate)
8113199	Tertiary	5	Trelegy Ellipta (fluticasone-umeclidinium-vilanterol)
			Breo Ellipta 100 (fluticasone-vilanterol)
			Incruse Ellipta (umeclidinium bromide)
			Anoro Ellipta (umeclidinium bromide-vilanterol trifenetate)
			Arnuity Ellipta 100 (fluticasone furoate)
8161968	Tertiary	5	Trelegy Ellipta (fluticasone-umeclidinium-vilanterol)
			Breo Ellipta 100 (fluticasone-vilanterol)
			Incruse Ellipta (umeclidinium bromide)
			Anoro Ellipta (umeclidinium bromide-vilanterol trifenetate)
			Arnuity Ellipta 100 (fluticasone furoate)
434281	Tertiary	5	Trelegy Ellipta (fluticasone-umeclidinium-vilanterol)
			Breo Ellipta 100 (fluticasone-vilanterol)
			Incruse Ellipta (umeclidinium bromide)
			Anoro Ellipta (umeclidinium bromide-vilanterol trifenetate)
			Arnuity Ellipta 100 (fluticasone furoate)

© 2025 Teng TW, et al. JAMA Health Forum.

8746242	Tertiary	5	Trelegy Ellipta (fluticasone-umeclidinium-vilanterol)
			Breo Ellipta 100 (fluticasone-vilanterol)
			Incruse Ellipta (umeclidinium bromide)
			Anoro Ellipta (umeclidinium bromide-vilanterol trifenetate)
			Arnuity Ellipta 100 (fluticasone furoate)
9333310	Tertiary	5	Trelegy Ellipta (fluticasone-umeclidinium-vilanterol)
			Breo Ellipta 100 (fluticasone-vilanterol)
			Incruse Ellipta (umeclidinium bromide)
			Anoro Ellipta (umeclidinium bromide-vilanterol trifenetate)
			Arnuity Ellipta 100 (fluticasone furoate)
6667061	Secondary	5	Bydureon (exenatide synthetic)
			Risperdal Consta (risperidone)
			Vivitrol (naltrexone)
			Bydureon Bcise (exenatide synthetic)
			Farxiga (dapagliflozin propanediol)
7918833	Tertiary	5	Humulin 70/30 Pen (insulin recombinant human; insulin susp isophane recombinant human)
			Humalog Kwikpen (insulin lispro recombinant)
			Humalog Kwikpen (insulin lispro recombinant)
			Humalog Mix 75/25 Kwikpen (insulin lispro protamine recombinant; insulin lispro recombinant)
			Humalog Mix 50/50 Kwikpen (insulin lispro protamine recombinant; insulin lispro recombinant)
8512297	Tertiary	5	Toujeo Solostar (insulin glargine recombinant)
			Admelog Solostar (insulin lispro)
			Soliqua 100/33 (insulin glargine/lixisenatide)
			Lantus (insulin glargine recombinant)
			Apidra Solostar (insulin glulisine recombinant)
8556864	Tertiary	5	Toujeo Solostar (insulin glargine recombinant)
			Admelog Solostar (insulin lispro)
			Soliqua 100/33 (insulin glargine/lixisenatide)
			Lantus (insulin glargine recombinant)
			Apidra Solostar (insulin glulisine recombinant)
8603044	Tertiary	5	Toujeo Solostar (insulin glargine recombinant)
			Admelog Solostar (insulin lispro)
			Soliqua 100/33 (insulin glargine/lixisenatide)
			Lantus (insulin glargine recombinant)
			Apidra Solostar (insulin glulisine recombinant)
8679069	Tertiary	5	Toujeo Solostar (insulin glargine recombinant)
			Admelog Solostar (insulin lispro)
			Soliqua 100/33 (insulin glargine/lixisenatide)
			Lantus (insulin glargine recombinant)
			Apidra Solostar (insulin glulisine recombinant)
8992486	Tertiary	5	Toujeo Solostar (insulin glargine recombinant)
			Admelog Solostar (insulin lispro)
			Soliqua 100/33 (insulin glargine/lixisenatide)
			Lantus (insulin glargine recombinant)
			Apidra Solostar (insulin glulisine recombinant)
9011391	Secondary	5	Toujeo Solostar (insulin glargine recombinant)
			Admelog Solostar (insulin lispro)
			Soliqua 100/33 (insulin glargine/lixisenatide)
			Lantus (insulin glargine recombinant)
			Apidra Solostar (insulin glulisine recombinant)
9233211	Tertiary	5	Toujeo Solostar (insulin glargine recombinant)

© 2025 Teng TW, et al. JAMA Health Forum.

			Admelog Solostar (insulin lispro)
			Soliqua 100/33 (insulin glargine/lixisenatide)
			Lantus (insulin glargine recombinant)
			Apidra Solostar (insulin glulisine recombinant)
9408979	Tertiary	5	Toujeo Solostar (insulin glargine recombinant)
			Admelog Solostar (insulin lispro)
			Soliqua 100/33 (insulin glargine/lixisenatide)
			Lantus (insulin glargine recombinant)
			Apidra Solostar (insulin glulisine recombinant)
9526844	Tertiary	5	Toujeo Solostar (insulin glargine recombinant)
			Admelog Solostar (insulin lispro)
			Soliqua 100/33 (insulin glargine/lixisenatide)
			Lantus (insulin glargine recombinant)
			Apidra Solostar (insulin glulisine recombinant)
9533105	Tertiary	5	Toujeo Solostar (insulin glargine recombinant)
			Admelog Solostar (insulin lispro)
			Soliqua 100/33 (insulin glargine/lixisenatide)
			Lantus (insulin glargine recombinant)
			Apidra Solostar (insulin glulisine recombinant)
9561331	Tertiary	5	Toujeo Solostar (insulin glargine recombinant)
			Admelog Solostar (insulin lispro)
			Soliqua 100/33 (insulin glargine/lixisenatide)
			Lantus (insulin glargine recombinant)
			Apidra Solostar (insulin glulisine recombinant)
9604008	Tertiary	5	Toujeo Solostar (insulin glargine recombinant)
			Admelog Solostar (insulin lispro)
			Soliqua 100/33 (insulin glargine/lixisenatide)
			Lantus (insulin glargine recombinant)
			Apidra Solostar (insulin glulisine recombinant)
9604009	Tertiary	5	Toujeo Solostar (insulin glargine recombinant)
			Admelog Solostar (insulin lispro)
			Soliqua 100/33 (insulin glargine/lixisenatide)
			Lantus (insulin glargine recombinant)
			Apidra Solostar (insulin glulisine recombinant)
9610409	Tertiary	5	Toujeo Solostar (insulin glargine recombinant)
			Admelog Solostar (insulin lispro)
			Soliqua 100/33 (insulin glargine/lixisenatide)
			Lantus (insulin glargine recombinant)
			Apidra Solostar (insulin glulisine recombinant)
9623189	Tertiary	5	Toujeo Solostar (insulin glargine recombinant)
			Admelog Solostar (insulin lispro)
			Soliqua 100/33 (insulin glargine/lixisenatide)
			Lantus (insulin glargine recombinant)
			Apidra Solostar (insulin glulisine recombinant)
9775954	Tertiary	5	Toujeo Solostar (insulin glargine recombinant)
			Admelog Solostar (insulin lispro)
			Soliqua 100/33 (insulin glargine/lixisenatide)
			Lantus (insulin glargine recombinant)
			Apidra Solostar (insulin glulisine recombinant)
9827379	Tertiary	5	Toujeo Solostar (insulin glargine recombinant)
			Admelog Solostar (insulin lispro)
			Soliqua 100/33 (insulin glargine/lixisenatide)
			Lantus (insulin glargine recombinant)

© 2025 Teng TW, et al. JAMA Health Forum.

			Apidra Solostar (insulin glulisine recombinant)
6446627	Tertiary	5	Qvar Redihaler (beclomethasone dipropionate)
			Airduo Digihaler 55 (fluticasone-salmeterol)
			Proair Hfa (albuterol sulfate)
			Proair Respiclick (albuterol sulfate)
			Armonair Digihaler (fluticasone)
6036976	Tertiary	5	Lupron Depot-3 (leuprolide acetate)
			Lupron Depot (leuprolide acetate)
			Lupron/Lupron Depot (leuprolide acetate)
			Lupron Depot (leuprolide acetate)
			Lupaneta Pack (leuprolide acetate; norethindrone acetate)
5683677	Secondary	5	Atrovent Hfa (ipratropium bromide)
			Alvesco (ciclesonide)
			Zetonna (ciclesonide)
			Qvar 80 (beclomethasone dipropionate)
			Qnasl (beclomethasone dipropionate)
6004297	Tertiary	5	Levemir Flextouch (insulin detemir recombinant)
			Novolog Penfill (insulin aspart recombinant)
			Novolog Mix 70/30 Flexpen (insulin aspart protamine recombinant; insulin aspart recombinant)
			Victoza (liraglutide recombinant)
			Norditropin Nordiflex (somatropin recombinant)
RE41956	Tertiary	5	Levemir Flextouch (insulin detemir recombinant)
			Novolog Penfill (insulin aspart recombinant)
			Novolog Mix 70/30 Flexpen (insulin aspart protamine recombinant; insulin aspart recombinant)
			Victoza (liraglutide recombinant)
			Norditropin Nordiflex (somatropin recombinant)
RE43834	Tertiary	5	Levemir Flextouch (insulin detemir recombinant)
			Novolog Penfill (insulin aspart recombinant)
			Novolog Mix 70/30 Flexpen (insulin aspart protamine recombinant; insulin aspart recombinant)
			Victoza (liraglutide recombinant)
			Norditropin Nordiflex (somatropin recombinant)
5422123	Tertiary	5	Zyflo Cr (zileuton)
			Paxil Cr (paroxetine hydrochloride)
			Sular (nisoldipine)
			Requip Xl (ropinirole hydrochloride)
			Dilacor Xr (diltiazem hydrochloride)
5082668	Tertiary	5	Concerta (methylphenidate hydrochloride)
			Ditropan Xl (oxybutynin chloride)
			Covera-Hs (verapamil hydrochloride)
			Glucotrol Xl (glipizide)
			Minipress Xl (prazosin hydrochloride)
4652441	Tertiary	5	Lupron Depot-3 (leuprolide acetate)
			Lupron Depot (leuprolide acetate)
			Lupron/Lupron Depot (leuprolide acetate)
			Lupron Depot (leuprolide acetate)
			Lupron Depot (leuprolide acetate)
4677191	Tertiary	5	Lupron Depot-3 (leuprolide acetate)
			Lupron Depot (leuprolide acetate)
			Lupron/Lupron Depot (leuprolide acetate)
			Lupron Depot (leuprolide acetate)
			Lupron Depot (leuprolide acetate)

© 2025 Teng TW, et al. JAMA Health Forum.

4728721	Tertiary	5	Lupron Depot-3 (leuprolide acetate)
			Lupron Depot (leuprolide acetate)
			Lupron/Lupron Depot (leuprolide acetate)
			Lupron Depot (leuprolide acetate)
			Lupron Depot (leuprolide acetate)
4849228	Tertiary	5	Lupron Depot-3 (leuprolide acetate)
			Lupron Depot (leuprolide acetate)
			Lupron/Lupron Depot (leuprolide acetate)
			Lupron Depot (leuprolide acetate)
			Lupron Depot (leuprolide acetate)
4917893	Tertiary	5	Lupron Depot-3 (leuprolide acetate)
			Lupron Depot (leuprolide acetate)
			Lupron/Lupron Depot (leuprolide acetate)
			Lupron Depot (leuprolide acetate)
			Lupron Depot (leuprolide acetate)
4954298	Secondary	5	Lupron Depot-3 (leuprolide acetate)
			Lupron Depot (leuprolide acetate)
			Lupron/Lupron Depot (leuprolide acetate)
			Lupron Depot (leuprolide acetate)
			Lupron Depot (leuprolide acetate)
5330767	Tertiary	5	Lupron Depot-3 (leuprolide acetate)
			Lupron Depot (leuprolide acetate)
			Lupron/Lupron Depot (leuprolide acetate)
			Lupron Depot (leuprolide acetate)
			Lupron Depot (leuprolide acetate)
5476663	Tertiary	5	Lupron Depot-3 (leuprolide acetate)
			Lupron Depot (leuprolide acetate)
			Lupron/Lupron Depot (leuprolide acetate)
			Lupron Depot (leuprolide acetate)
			Lupron Depot (leuprolide acetate)
5480656	Tertiary	5	Lupron Depot-3 (leuprolide acetate)
			Lupron Depot (leuprolide acetate)
			Lupron/Lupron Depot (leuprolide acetate)
			Lupron Depot (leuprolide acetate)
			Lupron Depot (leuprolide acetate)
5575987	Secondary	5	Lupron Depot-3 (leuprolide acetate)
			Lupron Depot (leuprolide acetate)
			Lupron/Lupron Depot (leuprolide acetate)
			Lupron Depot (leuprolide acetate)
			Lupron Depot (leuprolide acetate)
5631020	Tertiary	5	Lupron Depot-3 (leuprolide acetate)
			Lupron Depot (leuprolide acetate)
			Lupron/Lupron Depot (leuprolide acetate)
			Lupron Depot (leuprolide acetate)
			Lupron Depot (leuprolide acetate)
5631021	Tertiary	5	Lupron Depot-3 (leuprolide acetate)
			Lupron Depot (leuprolide acetate)
			Lupron/Lupron Depot (leuprolide acetate)
			Lupron Depot (leuprolide acetate)
			Lupron Depot (leuprolide acetate)
5643607	Tertiary	5	Lupron Depot-3 (leuprolide acetate)
			Lupron Depot (leuprolide acetate)
			Lupron/Lupron Depot (leuprolide acetate)

© 2025 Teng TW, et al. JAMA Health Forum.

			Lupron Depot (leuprolide acetate)
			Lupron Depot (leuprolide acetate)
5716640	Tertiary	5	Lupron Depot-3 (leuprolide acetate)
			Lupron Depot (leuprolide acetate)
			Lupron/Lupron Depot (leuprolide acetate)
			Lupron Depot (leuprolide acetate)
			Lupron Depot (leuprolide acetate)
10314977	Tertiary	4	Naloxone Hydrochloride (Autoinjector) (naloxone hydrochloride)
			Auvi-Q (epinephrine)
			Evzio (naloxone hydrochloride)
			Evzio (naloxone hydrochloride)
10335549	Tertiary	4	Naloxone Hydrochloride (Autoinjector) (naloxone hydrochloride)
			Auvi-Q (epinephrine)
			Evzio (naloxone hydrochloride)
			Evzio (naloxone hydrochloride)
10737028	Tertiary	4	Naloxone Hydrochloride (Autoinjector) (naloxone hydrochloride)
			Auvi-Q (epinephrine)
			Evzio (naloxone hydrochloride)
			Evzio (naloxone hydrochloride)
7731690	Tertiary	4	Naloxone Hydrochloride (Autoinjector) (naloxone hydrochloride)
			Auvi-Q (epinephrine)
			Evzio (naloxone hydrochloride)
			Evzio (naloxone hydrochloride)
7918823	Tertiary	4	Naloxone Hydrochloride (Autoinjector) (naloxone hydrochloride)
			Auvi-Q (epinephrine)
			Evzio (naloxone hydrochloride)
			Evzio (naloxone hydrochloride)
7947017	Tertiary	4	Naloxone Hydrochloride (Autoinjector) (naloxone hydrochloride)
			Auvi-Q (epinephrine)
			Evzio (naloxone hydrochloride)
			Evzio (naloxone hydrochloride)
8016788	Tertiary	4	Naloxone Hydrochloride (Autoinjector) (naloxone hydrochloride)
			Auvi-Q (epinephrine)
			Evzio (naloxone hydrochloride)
			Evzio (naloxone hydrochloride)
8313466	Tertiary	4	Naloxone Hydrochloride (Autoinjector) (naloxone hydrochloride)
			Auvi-Q (epinephrine)
			Evzio (naloxone hydrochloride)
			Evzio (naloxone hydrochloride)
8361029	Tertiary	4	Naloxone Hydrochloride (Autoinjector) (naloxone hydrochloride)
			Auvi-Q (epinephrine)
			Evzio (naloxone hydrochloride)
			Evzio (naloxone hydrochloride)
8425462	Tertiary	4	Naloxone Hydrochloride (Autoinjector) (naloxone hydrochloride)
			Auvi-Q (epinephrine)
			Evzio (naloxone hydrochloride)
			Evzio (naloxone hydrochloride)
8608698	Tertiary	4	Naloxone Hydrochloride (Autoinjector) (naloxone hydrochloride)
			Auvi-Q (epinephrine)
			Evzio (naloxone hydrochloride)
			Evzio (naloxone hydrochloride)
9056170	Tertiary	4	Naloxone Hydrochloride (Autoinjector) (naloxone hydrochloride)
			Auvi-Q (epinephrine)

© 2025 Teng TW, et al. JAMA Health Forum.

			Evzio (naloxone hydrochloride)
			Evzio (naloxone hydrochloride)
9737669	Tertiary	4	Naloxone Hydrochloride (Autoinjector) (naloxone hydrochloride)
			Auvi-Q (epinephrine)
			Evzio (naloxone hydrochloride)
			Evzio (naloxone hydrochloride)
11590286	Tertiary	4	Naloxone Hydrochloride (Autoinjector) (naloxone hydrochloride)
			Auvi-Q (epinephrine)
			Evzio (naloxone hydrochloride)
			Evzio (naloxone hydrochloride)
8470359	Secondary	4	Eligard Kit (leuprolide acetate)
			Eligard (leuprolide acetate)
			Eligard Kit (leuprolide acetate)
			Fensolvi Kit (leuprolide acetate)
9539333	Secondary	4	Eligard Kit (leuprolide acetate)
			Eligard (leuprolide acetate)
			Eligard Kit (leuprolide acetate)
			Fensolvi Kit (leuprolide acetate)
8603514	Tertiary	4	Sympazan (clobazam)
			Exservan (riluzole)
			Suboxone (buprenorphine hydrochloride; naloxone hydrochloride)
			Kynmobi (apomorphine hydrochloride)
7101866	Primary	4	Arnuity Ellipta 100 (fluticasone furoate)
			Veramyst (fluticasone furoate)
			Breo Ellipta 100 (fluticasone-vilanterol)
			Trelegy Ellipta (fluticasone-umeclidinium-vilanterol)
6495164	Secondary	4	Bydureon (exenatide synthetic)
			Vivitrol (naltrexone)
			Bydureon Bcise (exenatide synthetic)
			Farxiga (dapagliflozin propanediol)
9717852	Tertiary	4	Lantus (insulin glargine recombinant)
			Admelog Solostar (insulin lispro)
			Apidra Solostar (insulin glulisine recombinant)
			Soliqua 100/33 (insulin glargine/lixisenatide)
5964416	Tertiary	4	Striverdi Respimat (olodaterol)
			Striverdi Respimat (olodaterol) Combivent Respimat (albuterol-ipratopium)
			Combivent Respimat (albuterol-ipratopium)
			Stiolto Respimat (olodaterol hydrochloride-tiotropium bromide)
6149054	Tertiary	4	Striverdi Respimat (olodaterol)
			Striverdi Respimat (olodaterol) Combivent Respimat (albuterol-ipratopium)
			Combivent Respimat (albuterol-ipratopium)
			Stiolto Respimat (olodaterol hydrochloride-tiotropium bromide)
6176442	Tertiary	4	Striverdi Respimat (olodaterol)
			Striverdi Respimat (olodaterol) Combivent Respimat (albuterol-ipratopium)
			Combivent Respimat (albuterol-ipratopium)
			Stiolto Respimat (olodaterol hydrochloride-tiotropium bromide)
6453795	Tertiary	4	Striverdi Respimat (olodaterol)
			Striverdi Respimat (olodaterol) Combivent Respimat (albuterol-ipratopium)
			Combivent Respimat (albuterol-ipratopium)
			Stiolto Respimat (olodaterol hydrochloride-tiotropium bromide)

© 2025 Teng TW, et al. JAMA Health Forum.

6726124	Tertiary	4	Striverdi Respimat (olodaterol)
			Striverdi Respimat (olodaterol) Combivent Respimat (albuterol-ipratopium)
			Combivent Respimat (albuterol-ipratopium)
			Stiolto Respimat (olodaterol hydrochloride-tiotropium bromide)
6846413	Tertiary	4	Striverdi Respimat (olodaterol)
			Striverdi Respimat (olodaterol) Combivent Respimat (albuterol-ipratopium)
			Combivent Respimat (albuterol-ipratopium)
			Stiolto Respimat (olodaterol hydrochloride-tiotropium bromide)
6977042	Tertiary	4	Striverdi Respimat (olodaterol)
			Striverdi Respimat (olodaterol) Combivent Respimat (albuterol-ipratopium)
			Combivent Respimat (albuterol-ipratopium)
			Stiolto Respimat (olodaterol hydrochloride-tiotropium bromide)
6988496	Tertiary	4	Striverdi Respimat (olodaterol)
			Striverdi Respimat (olodaterol) Combivent Respimat (albuterol-ipratopium)
			Combivent Respimat (albuterol-ipratopium)
			Stiolto Respimat (olodaterol hydrochloride-tiotropium bromide)
7104470	Tertiary	4	Striverdi Respimat (olodaterol)
			Striverdi Respimat (olodaterol) Combivent Respimat (albuterol-ipratopium)
			Combivent Respimat (albuterol-ipratopium)
			Stiolto Respimat (olodaterol hydrochloride-tiotropium bromide)
7246615	Tertiary	4	Striverdi Respimat (olodaterol)
			Striverdi Respimat (olodaterol) Combivent Respimat (albuterol-ipratopium)
			Combivent Respimat (albuterol-ipratopium)
			Stiolto Respimat (olodaterol hydrochloride-tiotropium bromide)
7284474	Tertiary	4	Striverdi Respimat (olodaterol)
			Striverdi Respimat (olodaterol) Combivent Respimat (albuterol-ipratopium)
			Combivent Respimat (albuterol-ipratopium)
			Stiolto Respimat (olodaterol hydrochloride-tiotropium bromide)
7396341	Tertiary	4	Striverdi Respimat (olodaterol)
			Striverdi Respimat (olodaterol) Combivent Respimat (albuterol-ipratopium)
			Combivent Respimat (albuterol-ipratopium)
			Stiolto Respimat (olodaterol hydrochloride-tiotropium bromide)
7802568	Tertiary	4	Striverdi Respimat (olodaterol)
			Striverdi Respimat (olodaterol) Combivent Respimat (albuterol-ipratopium)
			Combivent Respimat (albuterol-ipratopium)
			Stiolto Respimat (olodaterol hydrochloride-tiotropium bromide)
7837235	Tertiary	4	Striverdi Respimat (olodaterol)
			Striverdi Respimat (olodaterol) Combivent Respimat (albuterol-ipratopium)
			Combivent Respimat (albuterol-ipratopium)
			Stiolto Respimat (olodaterol hydrochloride-tiotropium bromide)
7896264	Tertiary	4	Striverdi Respimat (olodaterol)
			Striverdi Respimat (olodaterol) Combivent Respimat (albuterol-ipratopium)
			Combivent Respimat (albuterol-ipratopium)

© 2025 Teng TW, et al. JAMA Health Forum.

			Stiolto Respimat (olodaterol hydrochloride-tiotropium bromide)
7988001	Tertiary	4	Striverdi Respimat (olodaterol)
			Striverdi Respimat (olodaterol) Combivent Respimat (albuterol-ipratopium)
			Combivent Respimat (albuterol-ipratopium)
			Stiolto Respimat (olodaterol hydrochloride-tiotropium bromide)
8733341	Tertiary	4	Striverdi Respimat (olodaterol)
			Striverdi Respimat (olodaterol) Combivent Respimat (albuterol-ipratopium)
			Combivent Respimat (albuterol-ipratopium)
			Stiolto Respimat (olodaterol hydrochloride-tiotropium bromide)
9027967	Tertiary	4	Striverdi Respimat (olodaterol)
			Striverdi Respimat (olodaterol) Combivent Respimat (albuterol-ipratopium)
			Combivent Respimat (albuterol-ipratopium)
			Stiolto Respimat (olodaterol hydrochloride-tiotropium bromide)
5866538	Secondary	4	Levemir Flextouch (insulin detemir recombinant)
			Ryzodeg 70/30 (insulin aspart; insulin degludec)
			Novolog Penfill (insulin aspart recombinant)
			Novolog Mix 70/30 Flexpen (insulin aspart protamine recombinant; insulin aspart recombinant)
9265893	Tertiary	4	Levemir Flextouch (insulin detemir recombinant)
			Victoza (liraglutide recombinant)
			Novolog Mix 70/30 Flexpen (insulin aspart protamine recombinant; insulin aspart recombinant)
			Novolog Penfill (insulin aspart recombinant)
5145684	Tertiary	4	Megace Es (megestrol acetate)
			Rapamune (sirolimus)
			Tricor (fenofibrate)
			Emend (aprepitant)
4938763	Secondary	4	Eligard Kit (leuprolide acetate)
			Eligard (leuprolide acetate)
			Eligard Kit (leuprolide acetate)
			Eligard Kit (leuprolide acetate)
5278201	Secondary	4	Eligard Kit (leuprolide acetate)
			Eligard (leuprolide acetate)
			Eligard Kit (leuprolide acetate)
			Eligard Kit (leuprolide acetate)
5324519	Secondary	4	Eligard Kit (leuprolide acetate)
			Eligard (leuprolide acetate)
			Eligard Kit (leuprolide acetate)
			Eligard Kit (leuprolide acetate)
5599552	Secondary	4	Eligard Kit (leuprolide acetate)
			Eligard (leuprolide acetate)
			Eligard Kit (leuprolide acetate)
			Eligard Kit (leuprolide acetate)
5739176	Secondary	4	Eligard Kit (leuprolide acetate)
			Eligard (leuprolide acetate)
			Eligard Kit (leuprolide acetate)
			Eligard Kit (leuprolide acetate)
6395293	Tertiary	4	Eligard Kit (leuprolide acetate)
			Eligard (leuprolide acetate)
			Eligard Kit (leuprolide acetate)
			Eligard Kit (leuprolide acetate)

© 2025 Teng TW, et al. JAMA Health Forum.

6565874	Tertiary	4	Eligard Kit (leuprolide acetate)
			Eligard (leuprolide acetate)
			Eligard Kit (leuprolide acetate)
			Eligard Kit (leuprolide acetate)
6626870	Tertiary	4	Eligard Kit (leuprolide acetate)
			Eligard (leuprolide acetate)
			Eligard Kit (leuprolide acetate)
			Eligard Kit (leuprolide acetate)
6773714	Tertiary	4	Eligard Kit (leuprolide acetate)
			Eligard (leuprolide acetate)
			Eligard Kit (leuprolide acetate)
			Eligard Kit (leuprolide acetate)
RE37950	Tertiary	4	Eligard Kit (leuprolide acetate)
			Eligard (leuprolide acetate)
			Eligard Kit (leuprolide acetate)
			Eligard Kit (leuprolide acetate)
6352684	Secondary	4	Qvar 80 (beclomethasone dipropionate)
			Proventil Hfa (albuterol)
			Proair Hfa (albuterol sulfate)
			Xopenex Hfa (levalbuterol)
5766573	Secondary	4	Qvar 80 (beclomethasone dipropionate)
			Atrovent Hfa (ipratropium bromide)
			Proventil Hfa (albuterol)
			Proair Hfa (albuterol sulfate)
4327725	Tertiary	4	Covera-Hs (verapamil hydrochloride)
			Glucotrol Xl (glipizide)
			Procardia Xl (nifedipine)
			Minipress Xl (prazosin hydrochloride)
10322239	Tertiary	3	Naloxone Hydrochloride (Autoinjector) (naloxone hydrochloride)
			Evzio (naloxone hydrochloride)
			Evzio (naloxone hydrochloride)
8939943	Tertiary	3	Naloxone Hydrochloride (Autoinjector) (naloxone hydrochloride)
			Evzio (naloxone hydrochloride)
			Evzio (naloxone hydrochloride)
9022022	Tertiary	3	Naloxone Hydrochloride (Autoinjector) (naloxone hydrochloride)
			Evzio (naloxone hydrochloride)
			Evzio (naloxone hydrochloride)
9474869	Tertiary	3	Naloxone Hydrochloride (Autoinjector) (naloxone hydrochloride)
			Evzio (naloxone hydrochloride)
			Evzio (naloxone hydrochloride)
8765167	Tertiary	3	Sympazan (clobazam)
			Exservan (riluzole)
			Kynmobi (apomorphine hydrochloride)
6217895	Tertiary	3	Iluvien (fluocinolone acetonide)
			Yutiq (fluocinolone acetonide)
			Retisert (fluocinolone acetonide)
6548078	Tertiary	3	Iluvien (fluocinolone acetonide)
			Yutiq (fluocinolone acetonide)
			Retisert (fluocinolone acetonide)
8021335	Tertiary	3	Otrexup (methotrexate)
			Makena (Autoinjector) (hydroxyprogesterone caproate)
			Xyosted (Autoinjector) (testosterone enanthate)
8562564	Tertiary	3	Otrexup (methotrexate)

© 2025 Teng TW, et al. JAMA Health Forum.

			Makena (Autoinjector) (hydroxyprogesterone caproate)
			Xyosted (Autoinjector) (testosterone enanthate)
9533102	Tertiary	3	Otrexup (methotrexate)
			Makena (Autoinjector) (hydroxyprogesterone caproate)
			Xyosted (Autoinjector) (testosterone enanthate)
9629959	Tertiary	3	Otrexup (methotrexate)
			Makena (Autoinjector) (hydroxyprogesterone caproate)
			Xyosted (Autoinjector) (testosterone enanthate)
RE44846	Tertiary	3	Anoro Ellipta (umeclidinium bromide-vilanterol trifenetate)
			Trelegy Ellipta (fluticasone-umeclidinium-vilanterol)
			Breo Ellipta 100 (fluticasone-vilanterol)
11446441	Tertiary	3	Otrexup (methotrexate)
			Makena (Autoinjector) (hydroxyprogesterone caproate)
			Xyosted (Autoinjector) (testosterone enanthate)
7731686	Tertiary	3	Auvi-Q (epinephrine)
			Evzio (naloxone hydrochloride)
			Evzio (naloxone hydrochloride)
7749194	Tertiary	3	Auvi-Q (epinephrine)
			Evzio (naloxone hydrochloride)
			Evzio (naloxone hydrochloride)
8021344	Tertiary	3	Auvi-Q (epinephrine)
			Evzio (naloxone hydrochloride)
			Evzio (naloxone hydrochloride)
8206360	Tertiary	3	Auvi-Q (epinephrine)
			Evzio (naloxone hydrochloride)
			Evzio (naloxone hydrochloride)
8226610	Tertiary	3	Auvi-Q (epinephrine)
			Evzio (naloxone hydrochloride)
			Evzio (naloxone hydrochloride)
8231573	Tertiary	3	Auvi-Q (epinephrine)
			Evzio (naloxone hydrochloride)
			Evzio (naloxone hydrochloride)
8926594	Tertiary	3	Auvi-Q (epinephrine)
			Evzio (naloxone hydrochloride)
			Evzio (naloxone hydrochloride)
9238108	Tertiary	3	Auvi-Q (epinephrine)
			Evzio (naloxone hydrochloride)
			Evzio (naloxone hydrochloride)
9278182	Tertiary	3	Auvi-Q (epinephrine)
			Evzio (naloxone hydrochloride)
			Evzio (naloxone hydrochloride)
9724471	Tertiary	3	Auvi-Q (epinephrine)
			Evzio (naloxone hydrochloride)
			Evzio (naloxone hydrochloride)
10960155	Tertiary	3	Auvi-Q (epinephrine)
			Evzio (naloxone hydrochloride)
			Evzio (naloxone hydrochloride)
8114833	Secondary	3	Victoza (liraglutide recombinant)
			Ozempic (semaglutide)
			Saxenda (liraglutide recombinant)
7439393	Secondary	3	Anoro Ellipta (umeclidinium bromide-vilanterol trifenetate)
			Trelegy Ellipta (fluticasone-umeclidinium-vilanterol)
			Breo Ellipta 100 (fluticasone-vilanterol)

© 2025 Teng TW, et al. JAMA Health Forum.

7488827	Primary	3	Anoro Ellipta (umeclidinium bromide-vilanterol trifenetate)
			Trelegy Ellipta (fluticasone-umeclidinium-vilanterol)
			Incruse Ellipta (umeclidinium bromide)
7498440	Primary	3	Anoro Ellipta (umeclidinium bromide-vilanterol trifenetate)
			Trelegy Ellipta (fluticasone-umeclidinium-vilanterol)
			Incruse Ellipta (umeclidinium bromide)
7629335	Secondary	3	Arnuity Ellipta 100 (fluticasone furoate)
			Trelegy Ellipta (fluticasone-umeclidinium-vilanterol) Breo
			Ellipta 100 (fluticasone-vilanterol)
7776895	Secondary	3	Anoro Ellipta (umeclidinium bromide-vilanterol trifenetate)
			Trelegy Ellipta (fluticasone-umeclidinium-vilanterol)
			Breo Ellipta 100 (fluticasone-vilanterol)
8183257	Secondary	3	Anoro Ellipta (umeclidinium bromide-vilanterol trifenetate)
			Trelegy Ellipta (fluticasone-umeclidinium-vilanterol)
			Incruse Ellipta (umeclidinium bromide)
8309572	Secondary	3	Anoro Ellipta (umeclidinium bromide-vilanterol trifenetate)
			Trelegy Ellipta (fluticasone-umeclidinium-vilanterol)
			Incruse Ellipta (umeclidinium bromide)
8511304	Tertiary	3	Anoro Ellipta (umeclidinium bromide-vilanterol trifenetate)
			Trelegy Ellipta (fluticasone-umeclidinium-vilanterol)
			Breo Ellipta 100 (fluticasone-vilanterol)
RE44874	Primary	3	Anoro Ellipta (umeclidinium bromide-vilanterol trifenetate)
			Trelegy Ellipta (fluticasone-umeclidinium-vilanterol)
			Breo Ellipta 100 (fluticasone-vilanterol)
6479065	Secondary	3	Bydureon (exenatide synthetic)
			Bydureon Bcise (exenatide synthetic)
			Farxiga (dapagliflozin propanediol)
6824822	Tertiary	3	Bydureon (exenatide synthetic)
			Bydureon Bcise (exenatide synthetic)
			Farxiga (dapagliflozin propanediol)
7223440	Tertiary	3	Bydureon (exenatide synthetic)
			Bydureon Bcise (exenatide synthetic)
			Farxiga (dapagliflozin propanediol)
6872700	Secondary	3	Bydureon (exenatide synthetic)
			Bydureon Bcise (exenatide synthetic)
			Farxiga (dapagliflozin propanediol)
6956026	Secondary	3	Bydureon (exenatide synthetic)
			Bydureon Bcise (exenatide synthetic)
			Farxiga (dapagliflozin propanediol)
7456254	Secondary	3	Bydureon (exenatide synthetic)
			Bydureon Bcise (exenatide synthetic)
			Farxiga (dapagliflozin propanediol)
7563871	Secondary	3	Bydureon (exenatide synthetic)
			Bydureon Bcise (exenatide synthetic)
			Farxiga (dapagliflozin propanediol)
7612176	Secondary	3	Bydureon (exenatide synthetic)
			Bydureon Bcise (exenatide synthetic)
			Farxiga (dapagliflozin propanediol)
7741269	Secondary	3	Bydureon (exenatide synthetic)
			Bydureon Bcise (exenatide synthetic)
			Farxiga (dapagliflozin propanediol)
8329648	Secondary	3	Bydureon (exenatide synthetic)
			Bydureon Bcise (exenatide synthetic)

© 2025 Teng TW, et al. JAMA Health Forum.

			Farxiga (dapagliflozin propanediol)
8431685	Secondary	3	Bydureon (exenatide synthetic)
			Bydureon Bcise (exenatide synthetic)
			Farxiga (dapagliflozin propanediol)
8461105	Secondary	3	Bydureon (exenatide synthetic)
			Bydureon Bcise (exenatide synthetic)
			Farxiga (dapagliflozin propanediol)
8216180	Tertiary	3	Bydureon (exenatide synthetic)
			Bydureon Bcise (exenatide synthetic)
			Farxiga (dapagliflozin propanediol)
8439864	Tertiary	3	Bydureon (exenatide synthetic)
			Bydureon Bcise (exenatide synthetic)
			Farxiga (dapagliflozin propanediol)
8906851	Secondary	3	Bydureon (exenatide synthetic)
			Bydureon Bcise (exenatide synthetic)
			Farxiga (dapagliflozin propanediol)
9238076	Secondary	3	Bydureon (exenatide synthetic)
			Bydureon Bcise (exenatide synthetic)
			Farxiga (dapagliflozin propanediol)
9320853	Tertiary	3	Bydureon (exenatide synthetic)
			Bydureon Bcise (exenatide synthetic)
			Farxiga (dapagliflozin propanediol)
6414126	Primary	3	Bydureon (exenatide synthetic)
			Bydureon Bcise (exenatide synthetic)
			Farxiga (dapagliflozin propanediol)
6515117	Primary	3	Bydureon (exenatide synthetic)
			Bydureon Bcise (exenatide synthetic)
			Farxiga (dapagliflozin propanediol)
6936590	Secondary	3	Bydureon (exenatide synthetic)
			Bydureon Bcise (exenatide synthetic)
			Farxiga (dapagliflozin propanediol)
7851502	Secondary	3	Bydureon (exenatide synthetic)
			Bydureon Bcise (exenatide synthetic)
			Farxiga (dapagliflozin propanediol)
7919598	Secondary	3	Bydureon (exenatide synthetic)
			Bydureon Bcise (exenatide synthetic)
			Farxiga (dapagliflozin propanediol)
8221786	Secondary	3	Bydureon (exenatide synthetic)
			Bydureon Bcise (exenatide synthetic)
			Farxiga (dapagliflozin propanediol)
8361972	Secondary	3	Bydureon (exenatide synthetic)
			Bydureon Bcise (exenatide synthetic)
			Farxiga (dapagliflozin propanediol)
8501698	Secondary	3	Bydureon (exenatide synthetic)
			Bydureon Bcise (exenatide synthetic)
			Farxiga (dapagliflozin propanediol)
8685934	Secondary	3	Bydureon (exenatide synthetic)
			Bydureon Bcise (exenatide synthetic)
			Farxiga (dapagliflozin propanediol)
8716251	Secondary	3	Bydureon (exenatide synthetic)
			Bydureon Bcise (exenatide synthetic)
			Farxiga (dapagliflozin propanediol)
8721615	Tertiary	3	Bydureon (exenatide synthetic)

© 2025 Teng TW, et al. JAMA Health Forum.

			Bydureon Bcise (exenatide synthetic)
			Farxiga (dapagliflozin propanediol)
8827963	Tertiary	3	Bydureon (exenatide synthetic)
			Bydureon Bcise (exenatide synthetic)
			Farxiga (dapagliflozin propanediol)
8758292	Tertiary	3	Bydureon (exenatide synthetic)
			Bydureon Bcise (exenatide synthetic)
			Farxiga (dapagliflozin propanediol)
8998876	Tertiary	3	Bydureon (exenatide synthetic)
			Bydureon Bcise (exenatide synthetic)
			Farxiga (dapagliflozin propanediol)
8690837	Tertiary	3	Bydureon (exenatide synthetic)
			Bydureon Bcise (exenatide synthetic)
			Farxiga (dapagliflozin propanediol)
9198925	Secondary	3	Bydureon (exenatide synthetic)
			Bydureon Bcise (exenatide synthetic)
			Farxiga (dapagliflozin propanediol)
6701917	Tertiary	3	Airduo Digihaler 55 (fluticasone-salmeterol)
			Armonair Digihaler (fluticasone)
			Proair Respiclick (albuterol sulfate)
6718972	Tertiary	3	Airduo Digihaler 55 (fluticasone-salmeterol)
			Armonair Digihaler (fluticasone)
			Proair Respiclick (albuterol sulfate)
6748947	Tertiary	3	Airduo Digihaler 55 (fluticasone-salmeterol)
			Armonair Digihaler (fluticasone)
			Proair Respiclick (albuterol sulfate)
6871646	Secondary	3	Airduo Digihaler 55 (fluticasone-salmeterol)
			Armonair Digihaler (fluticasone)
			Proair Respiclick (albuterol sulfate)
7540282	Tertiary	3	Airduo Digihaler 55 (fluticasone-salmeterol)
			Armonair Digihaler (fluticasone)
			Proair Respiclick (albuterol sulfate)
8006690	Tertiary	3	Airduo Digihaler 55 (fluticasone-salmeterol)
			Armonair Digihaler (fluticasone)
			Proair Respiclick (albuterol sulfate)
8651103	Tertiary	3	Airduo Digihaler 55 (fluticasone-salmeterol)
			Armonair Digihaler (fluticasone)
			Proair Respiclick (albuterol sulfate)
8978966	Tertiary	3	Airduo Digihaler 55 (fluticasone-salmeterol)
			Armonair Digihaler (fluticasone)
			Proair Respiclick (albuterol sulfate)
9216260	Tertiary	3	Airduo Digihaler 55 (fluticasone-salmeterol)
			Armonair Digihaler (fluticasone)
			Proair Respiclick (albuterol sulfate)
9463288	Tertiary	3	Airduo Digihaler 55 (fluticasone-salmeterol)
			Armonair Digihaler (fluticasone)
			Proair Respiclick (albuterol sulfate)
9731087	Tertiary	3	Airduo Digihaler 55 (fluticasone-salmeterol)
			Armonair Digihaler (fluticasone)
			Proair Respiclick (albuterol sulfate)
9782550	Tertiary	3	Airduo Digihaler 55 (fluticasone-salmeterol)
			Armonair Digihaler (fluticasone)
			Proair Respiclick (albuterol sulfate)

© 2025 Teng TW, et al. JAMA Health Forum.

9782551	Tertiary	3	Airduo Digihaler 55 (fluticasone-salmeterol)
			Armonair Digihaler (fluticasone)
			Proair Respiclick (albuterol sulfate)
10124131	Tertiary	3	Airduo Digihaler 55 (fluticasone-salmeterol)
			Armonair Digihaler (fluticasone)
			Proair Respiclick (albuterol sulfate)
10569034	Tertiary	3	Airduo Digihaler 55 (fluticasone-salmeterol)
			Armonair Digihaler (fluticasone)
			Proair Respiclick (albuterol sulfate)
10765820	Tertiary	3	Airduo Digihaler 55 (fluticasone-salmeterol)
			Armonair Digihaler (fluticasone)
			Proair Respiclick (albuterol sulfate)
10918816	Tertiary	3	Airduo Digihaler 55 (fluticasone-salmeterol)
			Armonair Digihaler (fluticasone)
			Proair Respiclick (albuterol sulfate)
11000653	Tertiary	3	Airduo Digihaler 55 (fluticasone-salmeterol)
			Armonair Digihaler (fluticasone)
			Proair Respiclick (albuterol sulfate)
11173259	Tertiary	3	Airduo Digihaler 55 (fluticasone-salmeterol)
			Armonair Digihaler (fluticasone)
			Proair Respiclick (albuterol sulfate)
11266796	Tertiary	3	Airduo Digihaler 55 (fluticasone-salmeterol)
			Armonair Digihaler (fluticasone)
			Proair Respiclick (albuterol sulfate)
11344685	Tertiary	3	Airduo Digihaler 55 (fluticasone-salmeterol)
			Armonair Digihaler (fluticasone)
			Proair Respiclick (albuterol sulfate)
11351317	Tertiary	3	Airduo Digihaler 55 (fluticasone-salmeterol)
			Armonair Digihaler (fluticasone)
			Proair Respiclick (albuterol sulfate)
11357935	Tertiary	3	Airduo Digihaler 55 (fluticasone-salmeterol)
			Armonair Digihaler (fluticasone)
			Proair Respiclick (albuterol sulfate)
11439777	Tertiary	3	Airduo Digihaler 55 (fluticasone-salmeterol)
			Armonair Digihaler (fluticasone)
			Proair Respiclick (albuterol sulfate)
11464923	Tertiary	3	Airduo Digihaler 55 (fluticasone-salmeterol)
			Armonair Digihaler (fluticasone)
			Proair Respiclick (albuterol sulfate)
6268343	Primary	3	Xultophy 100/3.6 (insulin degludec/liraglutide)
			Victoza (liraglutide recombinant)
			Saxenda (liraglutide recombinant)
6458924	Primary	3	Xultophy 100/3.6 (insulin degludec/liraglutide)
			Victoza (liraglutide recombinant)
			Saxenda (liraglutide recombinant)
7235627	Primary	3	Xultophy 100/3.6 (insulin degludec/liraglutide)
			Victoza (liraglutide recombinant)
			Saxenda (liraglutide recombinant)
7615532	Primary	3	Xultophy 100/3.6 (insulin degludec/liraglutide)
			Ryzodeg 70/30 (insulin aspart; insulin degludec)
			Tresiba (insulin degludec)
8846618	Secondary	3	Xultophy 100/3.6 (insulin degludec/liraglutide)
			Victoza (liraglutide recombinant)

© 2025 Teng TW, et al. JAMA Health Forum.

			Saxenda (liraglutide recombinant)
9615965	Tertiary	3	Kyleena (levonorgestrel)
			Mirena (levonorgestrel)
			Skyla (levonorgestrel)
9668912	Tertiary	3	Kyleena (levonorgestrel)
			Mirena (levonorgestrel)
			Skyla (levonorgestrel)
10561524	Secondary	3	Kyleena (levonorgestrel)
			Mirena (levonorgestrel)
			Skyla (levonorgestrel)
10987244	Tertiary	3	Kyleena (levonorgestrel)
			Mirena (levonorgestrel)
			Skyla (levonorgestrel)
6159498	Tertiary	3	Onsolis (fentanyl citrate)
			Belbuca (buprenorphine hydrochloride)
			Bunavail (buprenorphine hydrochloride; naloxone hydrochloride)
7579019	Secondary	3	Onsolis (fentanyl citrate)
			Belbuca (buprenorphine hydrochloride)
			Bunavail (buprenorphine hydrochloride; naloxone hydrochloride)
8479730	Tertiary	3	Arcapta Neohaler (indacaterol)
			Seebri Neohaler (glycopyrrolate)
			Utibron Neohaler (indacaterol-glycopyrrolate)
10022509	Tertiary	3	Qvar Redihaler (beclomethasone dipropionate)
			Qvar 80 (beclomethasone dipropionate)
			Proair Hfa (albuterol sulfate)
10086156	Tertiary	3	Qvar Redihaler (beclomethasone dipropionate)
			Qvar 80 (beclomethasone dipropionate)
			Proair Hfa (albuterol sulfate)
10695512	Tertiary	3	Qvar Redihaler (beclomethasone dipropionate)
			Qvar 80 (beclomethasone dipropionate)
			Proair Hfa (albuterol sulfate)
11395889	Tertiary	3	Qvar Redihaler (beclomethasone dipropionate)
			Qvar 80 (beclomethasone dipropionate)
			Proair Hfa (albuterol sulfate)
6730288	Tertiary	3	Verdeso (desonide)
			Olux E (clobetasol propionate)
			Finacea (azelaic acid)
RE39820	Primary	3	Spiriva Respimat 25 (tiotropium bromide)
			Spiriva Handihaler (tiotropium bromide monohydrate)
			Stiolto Respimat (olodaterol hydrochloride-tiotropium bromide)
6045501	Secondary	3	Thalomid (thalidomide)
			Pomalyst (pomalidomide)
			Revlimid (lenalidomide)
6315720	Secondary	3	Thalomid (thalidomide)
			Pomalyst (pomalidomide)
			Revlimid (lenalidomide)
6561976	Secondary	3	Thalomid (thalidomide)
			Pomalyst (pomalidomide)
			Revlimid (lenalidomide)
6561977	Secondary	3	Thalomid (thalidomide)
			Pomalyst (pomalidomide)
			Revlimid (lenalidomide)
6755784	Secondary	3	Thalomid (thalidomide)

© 2025 Teng TW, et al. JAMA Health Forum.

			Pomalyst (pomalidomide)
			Revlimid (lenalidomide)
6908432	Secondary	3	Thalomid (thalidomide)
			Pomalyst (pomalidomide)
			Revlimid (lenalidomide)
8204763	Secondary	3	Thalomid (thalidomide)
			Pomalyst (pomalidomide)
			Revlimid (lenalidomide)
8315886	Secondary	3	Thalomid (thalidomide)
			Pomalyst (pomalidomide)
			Revlimid (lenalidomide)
8589188	Secondary	3	Thalomid (thalidomide)
			Pomalyst (pomalidomide)
			Revlimid (lenalidomide)
8626531	Tertiary	3	Thalomid (thalidomide)
			Pomalyst (pomalidomide)
			Revlimid (lenalidomide)
5656286	Tertiary	3	Minivelle (estradiol)
			Estradiol (estradiol)
			Combipatch (estradiol)
6024976	Tertiary	3	Minivelle (estradiol)
			Estradiol (estradiol)
			Combipatch (estradiol)
7179483	Secondary	3	Gelnique (oxybutynin chloride)
			Oxytrol (oxybutynin)
			Oxytrol For Women (oxybutynin)
5775321	Tertiary	3	Zetonna (ciclesonide)
			Proventil Hfa (albuterol)
			Alvesco (ciclesonide)
6006745	Tertiary	3	Zetonna (ciclesonide)
			Proventil Hfa (albuterol)
			Alvesco (ciclesonide)
9283282	Secondary	3	Eligard Kit (leuprolide acetate)
			Eligard (leuprolide acetate)
			Eligard Kit (leuprolide acetate)
8486455	Tertiary	3	Eligard Kit (leuprolide acetate)
			Eligard (leuprolide acetate)
			Eligard Kit (leuprolide acetate)
8840916	Tertiary	3	Eligard Kit (leuprolide acetate)
			Eligard (leuprolide acetate)
			Eligard Kit (leuprolide acetate)
5958446	Tertiary	3	Estradiol (estradiol)
			Daytrana (methylphenidate)
			Combipatch (estradiol)
5733950	Secondary	3	Eligard Kit (leuprolide acetate)
			Eligard Kit (leuprolide acetate)
			Eligard Kit (leuprolide acetate)
6161724	Tertiary	3	Flovent Hfa 220 (fluticasone propionate)
			Ventolin Hfa (albuterol)
			Advair Hfa 45 (fluticasone-salmeterol)
6170717	Tertiary	3	Flovent Hfa 220 (fluticasone propionate)
			Ventolin Hfa (albuterol)
			Advair Hfa 45 (fluticasone-salmeterol)

© 2025 Teng TW, et al. JAMA Health Forum.

6251368	Secondary	3	Flovent Hfa 220 (fluticasone propionate)
			Ventolin Hfa (albuterol)
			Advair Hfa 45 (fluticasone-salmeterol)
6315173	Tertiary	3	Flovent Hfa 220 (fluticasone propionate)
			Ventolin Hfa (albuterol)
			Advair Hfa 45 (fluticasone-salmeterol)
6431168	Tertiary	3	Flovent Hfa 220 (fluticasone propionate)
			Ventolin Hfa (albuterol)
			Advair Hfa 45 (fluticasone-salmeterol)
6435372	Tertiary	3	Flovent Hfa 220 (fluticasone propionate)
			Ventolin Hfa (albuterol)
			Advair Hfa 45 (fluticasone-salmeterol)
6510969	Tertiary	3	Flovent Hfa 220 (fluticasone propionate)
			Ventolin Hfa (albuterol)
			Advair Hfa 45 (fluticasone-salmeterol)
6596260	Tertiary	3	Flovent Hfa 220 (fluticasone propionate)
			Ventolin Hfa (albuterol)
			Advair Hfa 45 (fluticasone-salmeterol)
6743413	Secondary	3	Flovent Hfa 220 (fluticasone propionate)
			Ventolin Hfa (albuterol)
			Advair Hfa 45 (fluticasone-salmeterol)
6938796	Tertiary	3	Flovent Hfa 220 (fluticasone propionate)
			Ventolin Hfa (albuterol)
			Advair Hfa 45 (fluticasone-salmeterol)
6966467	Tertiary	3	Flovent Hfa 220 (fluticasone propionate)
			Ventolin Hfa (albuterol)
			Advair Hfa 45 (fluticasone-salmeterol)
6997349	Tertiary	3	Flovent Hfa 220 (fluticasone propionate)
			Ventolin Hfa (albuterol)
			Advair Hfa 45 (fluticasone-salmeterol)
7107986	Tertiary	3	Flovent Hfa 220 (fluticasone propionate)
			Ventolin Hfa (albuterol)
			Advair Hfa 45 (fluticasone-salmeterol)
7143908	Tertiary	3	Flovent Hfa 220 (fluticasone propionate)
			Ventolin Hfa (albuterol)
			Advair Hfa 45 (fluticasone-salmeterol)
7350676	Tertiary	3	Flovent Hfa 220 (fluticasone propionate)
			Ventolin Hfa (albuterol)
			Advair Hfa 45 (fluticasone-salmeterol)
7500444	Tertiary	3	Flovent Hfa 220 (fluticasone propionate)
			Ventolin Hfa (albuterol)
			Advair Hfa 45 (fluticasone-salmeterol)
7832351	Tertiary	3	Flovent Hfa 220 (fluticasone propionate)
			Ventolin Hfa (albuterol)
			Advair Hfa 45 (fluticasone-salmeterol)
9861771	Tertiary	3	Flovent Hfa 220 (fluticasone propionate)
			Ventolin Hfa (albuterol)
			Advair Hfa 45 (fluticasone-salmeterol)
5164190	Tertiary	3	Androderm (testosterone)
			Oxytrol (oxybutynin)
			Alora (estradiol)
4992474	Primary	3	Advair Diskus 125/50 (fluticasone-salmeterol)
			Advair Hfa 45 (fluticasone-salmeterol)

© 2025 Teng TW, et al. JAMA Health Forum.

			Serevent Diskus (salmeterol xinafoate)
5126375	Primary	3	Advair Diskus 125/50 (fluticasone-salmeterol)
			Advair Hfa 45 (fluticasone-salmeterol)
			Serevent Diskus (salmeterol xinafoate)
5225445	Secondary	3	Advair Diskus 125/50 (fluticasone-salmeterol)
			Advair Hfa 45 (fluticasone-salmeterol)
			Serevent Diskus (salmeterol xinafoate)
5590645	Tertiary	3	Flovent Diskus 50 (fluticasone propionate)
			Advair Diskus 125/50 (fluticasone-salmeterol)
			Serevent Diskus (salmeterol xinafoate)
5860419	Tertiary	3	Flovent Diskus 50 (fluticasone propionate)
			Advair Diskus 125/50 (fluticasone-salmeterol)
			Serevent Diskus (salmeterol xinafoate)
6032666	Tertiary	3	Flovent Diskus 50 (fluticasone propionate)
			Advair Diskus 125/50 (fluticasone-salmeterol)
			Serevent Diskus (salmeterol xinafoate)
6378519	Tertiary	3	Flovent Diskus 50 (fluticasone propionate)
			Advair Diskus 125/50 (fluticasone-salmeterol)
			Serevent Diskus (salmeterol xinafoate)
6536427	Tertiary	3	Flovent Diskus 50 (fluticasone propionate)
			Advair Diskus 125/50 (fluticasone-salmeterol)
			Serevent Diskus (salmeterol xinafoate)
6792945	Tertiary	3	Flovent Diskus 50 (fluticasone propionate)
			Advair Diskus 125/50 (fluticasone-salmeterol)
			Serevent Diskus (salmeterol xinafoate)
7225808	Tertiary	3	Flovent Diskus 50 (fluticasone propionate)
			Advair Diskus 125/50 (fluticasone-salmeterol)
			Serevent Diskus (salmeterol xinafoate)
7389775	Tertiary	3	Flovent Diskus 50 (fluticasone propionate)
			Advair Diskus 125/50 (fluticasone-salmeterol)
			Serevent Diskus (salmeterol xinafoate)
5474978	Primary	3	Humalog Mix 75/25 Kwikpen (insulin lispro protamine recombinant; insulin lispro recombinant)
			Humalog Mix 50/50 Kwikpen (insulin lispro protamine recombinant; insulin lispro recombinant)
			Humalog Kwikpen (insulin lispro recombinant)
5514646	Primary	3	Humalog Mix 75/25 Kwikpen (insulin lispro protamine recombinant; insulin lispro recombinant)
			Humalog Mix 50/50 Kwikpen (insulin lispro protamine recombinant; insulin lispro recombinant)
			Humalog Kwikpen (insulin lispro recombinant)
4005063	Primary	3	Lupron Depot (leuprolide acetate)
			Lupron Depot (leuprolide acetate)
			Lupron/Lupron Depot (leuprolide acetate)
11357820	Secondary	2	Mounjaro (tirzepatide)
			Zepbound (tirzepatide)
8734394	Tertiary	2	Mounjaro (tirzepatide)
			Zepbound (tirzepatide)
9402957	Tertiary	2	Mounjaro (tirzepatide)
			Zepbound (tirzepatide)
9474780	Primary	2	Mounjaro (tirzepatide)
			Zepbound (tirzepatide)
8632802	Tertiary	2	Xelstrym (dextroamphetamine)
			Daytrana (methylphenidate)

© 2025 Teng TW, et al. JAMA Health Forum.

9034370	Tertiary	2	Xelstrym (dextroamphetamine)
			Daytrana (methylphenidate)
10716793	Secondary	2	Tyvaso (treprostinil)
			Tyvaso Dpi (treprostinil)
11723887	Secondary	2	Tyvaso (treprostinil)
			Tyvaso Dpi (treprostinil)
11826327	Secondary	2	Tyvaso (treprostinil)
			Tyvaso Dpi (treprostinil)
9593066	Secondary	2	Tyvaso (treprostinil)
			Tyvaso Dpi (treprostinil)
9604901	Secondary	2	Tyvaso (treprostinil)
			Tyvaso Dpi (treprostinil)
9914802	Secondary	2	Eligard (leuprolide acetate)
			Fensolvi Kit (leuprolide acetate)
7033605	Secondary	2	Ozurdex (dexamethasone)
			Durysta (bimatoprost)
10058511	Secondary	2	Eysuvis (loteprednol etabonate)
			Inveltys (loteprednol etabonate)
10688045	Secondary	2	Eysuvis (loteprednol etabonate)
			Inveltys (loteprednol etabonate)
11642317	Secondary	2	Eysuvis (loteprednol etabonate)
			Inveltys (loteprednol etabonate)
9056057	Tertiary	2	Tudorza Pressair (aclidinium bromide)
			Duaklir Pressair (aclidinium bromide-formoterol fumerate)
9393213	Tertiary	2	Eysuvis (loteprednol etabonate)
			Inveltys (loteprednol etabonate)
9532955	Secondary	2	Eysuvis (loteprednol etabonate)
			Inveltys (loteprednol etabonate)
9737491	Secondary	2	Eysuvis (loteprednol etabonate)
			Inveltys (loteprednol etabonate)
9827191	Secondary	2	Eysuvis (loteprednol etabonate)
			Inveltys (loteprednol etabonate)
9855221	Tertiary	2	Suboxone (buprenorphine hydrochloride; naloxone hydrochloride)
			Kynmobi (apomorphine hydrochloride)
9931305	Tertiary	2	Seebri Neohaler (glycopyrrolate)
			Utibron Neohaler (indacaterol-glycopyrrolate)
6681768	Tertiary	2	Tudorza Pressair (aclidinium bromide)
			Duaklir Pressair (aclidinium bromide-formoterol fumerate)
7078412	Primary	2	Tudorza Pressair (aclidinium bromide)
			Duaklir Pressair (aclidinium bromide-formoterol fumerate)
8051851	Tertiary	2	Tudorza Pressair (aclidinium bromide)
			Duaklir Pressair (aclidinium bromide-formoterol fumerate)
9056100	Secondary	2	Tudorza Pressair (aclidinium bromide)
			Duaklir Pressair (aclidinium bromide-formoterol fumerate)
9333195	Secondary	2	Tudorza Pressair (aclidinium bromide)
			Duaklir Pressair (aclidinium bromide-formoterol fumerate)
10034867	Secondary	2	Tudorza Pressair (aclidinium bromide)
			Duaklir Pressair (aclidinium bromide-formoterol fumerate)
10085974	Secondary	2	Tudorza Pressair (aclidinium bromide)
			Duaklir Pressair (aclidinium bromide-formoterol fumerate)
10588895	Secondary	2	Tudorza Pressair (aclidinium bromide)
			Duaklir Pressair (aclidinium bromide-formoterol fumerate)
RE46417	Primary	2	Tudorza Pressair (aclidinium bromide)

© 2025 Teng TW, et al. JAMA Health Forum.

			Duaklir Pressair (aclidinium bromide-formoterol fumerate)
11000517	Secondary	2	Tudorza Pressair (aclidinium bromide)
			Duaklir Pressair (aclidinium bromide-formoterol fumerate)
6375972	Tertiary	2	Iluvien (fluocinolone acetonide)
			Yutiq (fluocinolone acetonide)
8252307	Tertiary	2	Iluvien (fluocinolone acetonide)
			Yutiq (fluocinolone acetonide)
8871241	Tertiary	2	Iluvien (fluocinolone acetonide)
			Yutiq (fluocinolone acetonide)
9180259	Tertiary	2	Makena (Autoinjector) (hydroxyprogesterone caproate)
			Xyosted (Autoinjector) (testosterone enanthate)
RE44847	Secondary	2	Otrexup (methotrexate)
			Xyosted (Autoinjector) (testosterone enanthate)
11446440	Tertiary	2	Makena (Autoinjector) (hydroxyprogesterone caproate)
			Xyosted (Autoinjector) (testosterone enanthate)
11497753	Tertiary	2	Otrexup (methotrexate)
			Xyosted (Autoinjector) (testosterone enanthate)
8627816	Tertiary	2	Evzio (naloxone hydrochloride)
			Evzio (naloxone hydrochloride)
9517307	Tertiary	2	Evzio (naloxone hydrochloride)
			Evzio (naloxone hydrochloride)
10143972	Secondary	2	Evzio (naloxone hydrochloride)
			Evzio (naloxone hydrochloride)
10220158	Tertiary	2	Evzio (naloxone hydrochloride)
			Evzio (naloxone hydrochloride)
11097063	Tertiary	2	Ozempic (semaglutide)
			Saxenda (liraglutide recombinant)
11311679	Tertiary	2	Ozempic (semaglutide)
			Saxenda (liraglutide recombinant)
11446443	Tertiary	2	Ozempic (semaglutide)
			Saxenda (liraglutide recombinant)
6537983	Secondary	2	Trelegy Ellipta (fluticasone-umeclidinium-vilanterol)
			Breo Ellipta 100 (fluticasone-vilanterol)
6759398	Secondary	2	Trelegy Ellipta (fluticasone-umeclidinium-vilanterol)
			Breo Ellipta 100 (fluticasone-vilanterol)
6878698	Secondary	2	Trelegy Ellipta (fluticasone-umeclidinium-vilanterol)
			Breo Ellipta 100 (fluticasone-vilanterol)
9750726	Secondary	2	Anoro Ellipta (umeclidinium bromide-vilanterol trifenetate)
			Trelegy Ellipta (fluticasone-umeclidinium-vilanterol)
11090294	Secondary	2	Anoro Ellipta (umeclidinium bromide-vilanterol trifenetate)
			Trelegy Ellipta (fluticasone-umeclidinium-vilanterol)
5424286	Primary	2	Bydureon (exenatide synthetic)
			Bydureon Bcise (exenatide synthetic)
6858576	Secondary	2	Bydureon (exenatide synthetic)
			Bydureon Bcise (exenatide synthetic)
9884092	Secondary	2	Bydureon (exenatide synthetic)
			Bydureon Bcise (exenatide synthetic)
6715485	Tertiary	2	Onzetra Xsail (sumatriptan succinate)
			Fluticasone Propionate (xhance)
7975690	Secondary	2	Onzetra Xsail (sumatriptan succinate)
			Fluticasone Propionate (xhance)
8327844	Secondary	2	Onzetra Xsail (sumatriptan succinate)
			Fluticasone Propionate (xhance)

© 2025 Teng TW, et al. JAMA Health Forum.

8550073	Tertiary	2	Onzetra Xsail (sumatriptan succinate)
			Fluticasone Propionate (xhance)
8978647	Tertiary	2	Onzetra Xsail (sumatriptan succinate)
			Fluticasone Propionate (xhance)
10076614	Tertiary	2	Onzetra Xsail (sumatriptan succinate)
			Fluticasone Propionate (xhance)
10076615	Secondary	2	Onzetra Xsail (sumatriptan succinate)
			Fluticasone Propionate (xhance)
10124132	Secondary	2	Onzetra Xsail (sumatriptan succinate)
			Fluticasone Propionate (xhance)
10478574	Secondary	2	Onzetra Xsail (sumatriptan succinate)
			Fluticasone Propionate (xhance)
8714149	Tertiary	2	Airduo Digihaler 55 (fluticasone-salmeterol)
			Armonair Digihaler (fluticasone)
9616024	Tertiary	2	Airduo Digihaler 55 (fluticasone-salmeterol)
			Armonair Digihaler (fluticasone)
10195375	Tertiary	2	Airduo Digihaler 55 (fluticasone-salmeterol)
			Armonair Digihaler (fluticasone)
RE45313	Primary	2	Adlyxin (lixisenatide)
			Soliqua 100/33 (insulin glargine/lixisenatide)
9707176	Secondary	2	Adlyxin (lixisenatide)
			Soliqua 100/33 (insulin glargine/lixisenatide)
9821032	Secondary	2	Adlyxin (lixisenatide)
			Soliqua 100/33 (insulin glargine/lixisenatide)
7252839	Tertiary	2	Kyleena (levonorgestrel)
			Skyla (levonorgestrel)
11628088	Tertiary	2	Kyleena (levonorgestrel)
			Skyla (levonorgestrel)
8147866	Tertiary	2	Belbuca (buprenorphine hydrochloride)
			Bunavail (buprenorphine hydrochloride; naloxone hydrochloride)
9655843	Tertiary	2	Belbuca (buprenorphine hydrochloride)
			Bunavail (buprenorphine hydrochloride; naloxone hydrochloride)
6521260	Secondary	2	Seebri Neohaler (glycopyrrolate)
			Utibron Neohaler (indacaterol-glycopyrrolate)
6528678	Secondary	2	Seebri Neohaler (glycopyrrolate)
			Utibron Neohaler (indacaterol-glycopyrrolate)
6878721	Primary	2	Arcapta Neohaler (indacaterol)
			Utibron Neohaler (indacaterol-glycopyrrolate)
7229607	Secondary	2	Seebri Neohaler (glycopyrrolate)
			Utibron Neohaler (indacaterol-glycopyrrolate)
7736670	Tertiary	2	Seebri Neohaler (glycopyrrolate)
			Utibron Neohaler (indacaterol-glycopyrrolate)
8029768	Secondary	2	Seebri Neohaler (glycopyrrolate)
			Utibron Neohaler (indacaterol-glycopyrrolate)
8048451	Secondary	2	Seebri Neohaler (glycopyrrolate)
			Utibron Neohaler (indacaterol-glycopyrrolate)
8067437	Secondary	2	Seebri Neohaler (glycopyrrolate)
			Utibron Neohaler (indacaterol-glycopyrrolate)
8182838	Tertiary	2	Seebri Neohaler (glycopyrrolate)
			Utibron Neohaler (indacaterol-glycopyrrolate)
8303991	Tertiary	2	Seebri Neohaler (glycopyrrolate)
			Utibron Neohaler (indacaterol-glycopyrrolate)
8435567	Tertiary	2	Seebri Neohaler (glycopyrrolate)

© 2025 Teng TW, et al. JAMA Health Forum.

			Utibron Neohaler (indacaterol-glycopyrrolate)
8580306	Secondary	2	Seebri Neohaler (glycopyrrolate)
			Utibron Neohaler (indacaterol-glycopyrrolate)
8658673	Secondary	2	Arcapta Neohaler (indacaterol)
			Utibron Neohaler (indacaterol-glycopyrrolate)
8796307	Secondary	2	Arcapta Neohaler (indacaterol)
			Utibron Neohaler (indacaterol-glycopyrrolate)
8956661	Tertiary	2	Seebri Neohaler (glycopyrrolate)
			Utibron Neohaler (indacaterol-glycopyrrolate)
9931304	Secondary	2	Seebri Neohaler (glycopyrrolate)
			Utibron Neohaler (indacaterol-glycopyrrolate)
9962338	Tertiary	2	Seebri Neohaler (glycopyrrolate)
			Utibron Neohaler (indacaterol-glycopyrrolate)
8132712	Tertiary	2	Qvar Redihaler (beclomethasone dipropionate)
			Proair Hfa (albuterol sulfate)
9089567	Secondary	2	Abilify Mycite Kit (aripiprazole)
			Abilify Maintena Kit (aripiprazole)
7056916	Primary	2	Striverdi Respimat (olodaterol)
			Stiolto Respimat (olodaterol hydrochloride-tiotropium bromide)
7220742	Primary	2	Striverdi Respimat (olodaterol)
			Stiolto Respimat (olodaterol hydrochloride-tiotropium bromide)
7491719	Secondary	2	Striverdi Respimat (olodaterol)
			Stiolto Respimat (olodaterol hydrochloride-tiotropium bromide)
7727984	Secondary	2	Striverdi Respimat (olodaterol)
			Stiolto Respimat (olodaterol hydrochloride-tiotropium bromide)
7786111	Secondary	2	Striverdi Respimat (olodaterol)
			Stiolto Respimat (olodaterol hydrochloride-tiotropium bromide)
8034809	Secondary	2	Striverdi Respimat (olodaterol)
			Stiolto Respimat (olodaterol hydrochloride-tiotropium bromide)
8044046	Secondary	2	Striverdi Respimat (olodaterol)
			Stiolto Respimat (olodaterol hydrochloride-tiotropium bromide)
9968659	Secondary	2	Victoza (liraglutide recombinant)
			Saxenda (liraglutide recombinant)
8201556	Tertiary	2	Arnuity Ellipta 100 (fluticasone furoate)
			Incruse Ellipta (umeclidinium bromide)
7361787	Primary	2	Anoro Ellipta (umeclidinium bromide-vilanterol trifenetate)
			Breo Ellipta 100 (fluticasone-vilanterol)
5635517	Primary	2	Revlimid (lenalidomide)
			Pomalyst (pomalidomide)
5785053	Tertiary	2	Mirena (levonorgestrel)
			Skyla (levonorgestrel)
5776432	Secondary	2	Qvar 80 (beclomethasone dipropionate)
			Qnasl (beclomethasone dipropionate)
5601839	Tertiary	2	Oxytrol (oxybutynin)
			Oxytrol For Women (oxybutynin)
5834010	Tertiary	2	Oxytrol (oxybutynin)
			Oxytrol For Women (oxybutynin)
6743441	Secondary	2	Oxytrol (oxybutynin)
			Oxytrol For Women (oxybutynin)
7081249	Secondary	2	Oxytrol (oxybutynin)
			Oxytrol For Women (oxybutynin)
7081250	Secondary	2	Oxytrol (oxybutynin)
			Oxytrol For Women (oxybutynin)

© 2025 Teng TW, et al. JAMA Health Forum.

7081251	Secondary	2	Oxytrol (oxybutynin)
			Oxytrol For Women (oxybutynin)
7081252	Secondary	2	Oxytrol (oxybutynin)
			Oxytrol For Women (oxybutynin)
5482934	Primary	2	Zetonna (ciclesonide)
			Alvesco (ciclesonide)
6036942	Tertiary	2	Zetonna (ciclesonide)
			Alvesco (ciclesonide)
6120752	Tertiary	2	Zetonna (ciclesonide)
			Alvesco (ciclesonide)
6264923	Secondary	2	Zetonna (ciclesonide)
			Alvesco (ciclesonide)
8371292	Secondary	2	Zetonna (ciclesonide)
			Alvesco (ciclesonide)
5266329	Tertiary	2	Gynazole-1 (butoconazole nitrate)
			Clindesse (clindamycin phosphate)
5993856	Secondary	2	Gynazole-1 (butoconazole nitrate)
			Clindesse (clindamycin phosphate)
6299900	Secondary	2	Axiron (testosterone)
			Evamist (estradiol)
6818226	Tertiary	2	Axiron (testosterone)
			Evamist (estradiol)
6923983	Tertiary	2	Axiron (testosterone)
			Evamist (estradiol)
5266325	Tertiary	2	Vantas (histrelin acetate)
			Supprelin La (histrelin acetate)
5292515	Tertiary	2	Vantas (histrelin acetate)
			Supprelin La (histrelin acetate)
7029659	Tertiary	2	Verdeso (desonide)
			Olux E (clobetasol propionate)
8460641	Secondary	2	Verdeso (desonide)
			Olux E (clobetasol propionate)
8962000	Secondary	2	Verdeso (desonide)
			Olux E (clobetasol propionate)
5178878	Secondary	2	Fazaclo Odt (clozapine)
			Orapred Odt (prednisolone sodium phosphate)
4907583	Tertiary	2	Pulmicort 160 (budesonide)
			Pulmicort Flexhaler 80 (budesonide)
5792477	Tertiary	2	Vivitrol (naltrexone)
			Risperdal Consta (risperidone)
5916598	Tertiary	2	Vivitrol (naltrexone)
			Risperdal Consta (risperidone)
6110503	Tertiary	2	Vivitrol (naltrexone)
			Risperdal Consta (risperidone)
6194006	Tertiary	2	Vivitrol (naltrexone)
			Risperdal Consta (risperidone)
6264987	Tertiary	2	Vivitrol (naltrexone)
			Risperdal Consta (risperidone)
6379703	Tertiary	2	Vivitrol (naltrexone)
			Risperdal Consta (risperidone)
6379704	Tertiary	2	Vivitrol (naltrexone)
			Risperdal Consta (risperidone)
6403114	Tertiary	2	Vivitrol (naltrexone)

© 2025 Teng TW, et al. JAMA Health Forum.

			Risperdal Consta (risperidone)
6534092	Tertiary	2	Vivitrol (naltrexone)
			Risperdal Consta (risperidone)
6596316	Tertiary	2	Vivitrol (naltrexone)
			Risperdal Consta (risperidone)
5225183	Secondary	2	Proventil Hfa (albuterol)
			Xopenex Hfa (levalbuterol)
5439670	Secondary	2	Proventil Hfa (albuterol)
			Xopenex Hfa (levalbuterol)
5326570	Tertiary	2	Equetro (carbamazepine)
			Carbatrol (carbamazepine)
5912013	Secondary	2	Equetro (carbamazepine)
			Carbatrol (carbamazepine)
5223261	Tertiary	2	Menostar (estradiol)
			Climara (estradiol)
5723147	Tertiary	2	Depodur (morphine sulfate)
			Depocyt (cytarabine)
5962016	Tertiary	2	Depodur (morphine sulfate)
			Depocyt (cytarabine)
6071534	Tertiary	2	Depodur (morphine sulfate)
			Depocyt (cytarabine)
5690958	Tertiary	2	Chloraprep One-Step Frepp (chlorhexidine gluconate; isopropyl alcohol)
			Chloraprep One-Step Sepp (chlorhexidine gluconate; isopropyl alcohol)
4615697	Tertiary	2	Striant (testosterone)
			Crinone (progesterone)
9463289	Tertiary	2	Qvar 80 (beclomethasone dipropionate)
			Proair Hfa (albuterol sulfate)
9808587	Tertiary	2	Qvar 80 (beclomethasone dipropionate)
			Proair Hfa (albuterol sulfate)
5658549	Secondary	2	Flovent Hfa 220 (fluticasone propionate)
			Advair Hfa 45 (fluticasone-salmeterol)
5674472	Tertiary	2	Flovent Hfa 220 (fluticasone propionate)
			Advair Hfa 45 (fluticasone-salmeterol)
6253762	Tertiary	2	Flovent Hfa 220 (fluticasone propionate)
			Advair Hfa 45 (fluticasone-salmeterol)
6546928	Tertiary	2	Flovent Hfa 220 (fluticasone propionate)
			Advair Hfa 45 (fluticasone-salmeterol)
5192741	Secondary	2	Trelstar (triptorelin pamoate)
			Trelstar Depot (triptorelin pamoate)
5225205	Tertiary	2	Trelstar (triptorelin pamoate)
			Trelstar Depot (triptorelin pamoate)
5776885	Secondary	2	Trelstar (triptorelin pamoate)
			Trelstar Depot (triptorelin pamoate)
5270305	Secondary	2	Advair Diskus 125/50 (fluticasone-salmeterol)
			Advair Hfa 45 (fluticasone-salmeterol)
RE40045	Secondary	2	Advair Diskus 125/50 (fluticasone-salmeterol)
			Advair Hfa 45 (fluticasone-salmeterol)
4968299	Tertiary	2	Genotropin Preservative Free (somatropin recombinant)
			Caverject Impulse (alprostadil)
5501673	Tertiary	2	Genotropin Preservative Free (somatropin recombinant)
			Caverject Impulse (alprostadil)
5716338	Tertiary	2	Genotropin Preservative Free (somatropin recombinant)
			Caverject Impulse (alprostadil)

© 2025 Teng TW, et al. JAMA Health Forum.

5618913	Primary	2	Novolog Penfill (insulin aspart recombinant)
			Novolog Mix 70/30 Flexpen (insulin aspart protamine recombinant; insulin aspart recombinant)
5633352	Secondary	2	Genotropin Preservative Free (somatropin recombinant)
			Norditropin Nordiflex (somatropin recombinant)
4519801	Tertiary	2	Ditropan Xl (oxybutynin chloride)
			Concerta (methylphenidate hydrochloride)
6395292	Tertiary	2	Viadur (leuprolide acetate)
			Sandostatin Lar (octreotide acetate)
4335121	Primary	2	Flovent Diskus 50 (fluticasone propionate)
			Advair Diskus 125/50 (fluticasone-salmeterol)
5290815	Secondary	2	Advair Diskus 125/50 (fluticasone-salmeterol)
			Serevent Diskus (salmeterol xinafoate)
5461031	Secondary	2	Humalog Mix 75/25 Kwikpen (insulin lispro protamine recombinant; insulin lispro recombinant)
			Humalog Mix 50/50 Kwikpen (insulin lispro protamine recombinant; insulin lispro recombinant)
5747642	Secondary	2	Humalog Mix 75/25 Kwikpen (insulin lispro protamine recombinant; insulin lispro recombinant)
			Humalog Mix 50/50 Kwikpen (insulin lispro protamine recombinant; insulin lispro recombinant)
4690825	Tertiary	2	Carac (fluorouracil)
			Retin-A Micro (tretinoin)
4839177	Tertiary	2	Paxil Cr (paroxetine hydrochloride)
			Dilacor Xr (diltiazem hydrochloride)
5474783	Tertiary	2	Estradiol (estradiol)
			Combipatch (estradiol)
4379454	Tertiary	2	Estraderm (estradiol)
			Testoderm Tts (testosterone)
5501236	Tertiary	2	Nicotrol (nicotine)
			Nicotrol (nicotine)
6098632	Tertiary	2	Nicotrol (nicotine)
			Nicotrol (nicotine)
5814342	Tertiary	2	Lupron Depot-3 (leuprolide acetate)
			Lupron Depot (leuprolide acetate)
4100274	Primary	2	Zoladex (goserelin acetate)
			Zoladex (goserelin acetate)
4767628	Secondary	2	Zoladex (goserelin acetate)
			Zoladex (goserelin acetate)
5366734	Secondary	2	Zoladex (goserelin acetate)
			Zoladex (goserelin acetate)
7118552	Tertiary	2	Zoladex (goserelin acetate)
			Zoladex (goserelin acetate)
7220247	Tertiary	2	Zoladex (goserelin acetate)
			Zoladex (goserelin acetate)
7500964	Tertiary	2	Zoladex (goserelin acetate)
			Zoladex (goserelin acetate)
4946687	Secondary	2	Covera-Hs (verapamil hydrochloride)
			Dynacirc Cr (isradipine)
5030456	Secondary	2	Covera-Hs (verapamil hydrochloride)
			Dynacirc Cr (isradipine)
8921326	Secondary	2	Lupron Depot (leuprolide acetate)
			Lupron/Lupron Depot (leuprolide acetate)
4880631	Tertiary	2	Tiamate (diltiazem malate)

© 2025 Teng TW, et al. JAMA Health Forum.

			Teczem (diltiazem malate; enalapril maleate)
4968507	Tertiary	2	Tiamate (diltiazem malate)
			Teczem (diltiazem malate; enalapril maleate)
4576604	Tertiary	2	Efidac 24 Chlorpheniramine Maleat (chlorpheniramine maleate)
			Efidac 24 Pseudoephedrine Hcl (pseudoephedrine hydrochloride)
4801461	Secondary	2	Efidac 24 Pseudoephedrine Hcl/Bro (brompheniramine maleate)
			Efidac 24 Pseudoephedrine Hcl (pseudoephedrine hydrochloride)
4144317	Tertiary	2	Estraderm (estradiol)
			Duragesic (fentanyl)
3845770	Tertiary	2	Lopressor (metoprolol fumarate)
			Procardia Xl (nifedipine)
3916899	Tertiary	2	Lopressor (metoprolol fumarate)
			Procardia Xl (nifedipine)
4765989	Tertiary	2	Procardia Xl (nifedipine)
			Minipress Xl (prazosin hydrochloride)
4673405	Tertiary	2	Efidac 24 Pseudoephedrine Hcl/Bro (brompheniramine maleate)
			Efidac 24 Chlorpheniramine Maleat (chlorpheniramine maleate)

© 2025 Teng TW, et al. JAMA Health Forum.

eTable 4: **Patent portfolios of drug-device combinations approved by the FDA, 1986-2023**

Brand name	Generic name	NDA	Total patents	Primary patents	Secondary patents	Tertiary patents
ABILIFY MAINTENA KIT	aripiprazole	202971	16	1	14	1
ABILIFY MYCITE KIT	aripiprazole	207202	38	0	10	28
ABRAXANE	paclitaxel	21660	20	0	19	1
ACUVUE THERAVISION WITH KETOTIFEN	ketotifen fumarate	22388	2	0	1	1
ADASUVE	loxapine	22549	18	0	8	10
ADHANSIA XR	methylphenidate hydrochloride	212038	12	0	10	2
ADLARITY	donepezil hydrochloride	212304	9	0	3	6
ADLYXIN	lixisenatide	208471	16	1	5	10
ADMELOG SOLOSTAR	insulin lispro	209196	19	0	1	18
ADVAIR DISKUS 125/50	fluticasone-salmeterol	21077	16	3	4	9
ADVAIR HFA 45	fluticasone-salmeterol	21254	29	2	6	21
AFREZZA	insulin recombinant human	22472	48	0	19	29
AIRDUO DIGIHALER 55	fluticasone-salmeterol	208799	34	0	2	32
ALLEGRA D 24 HOUR	fexofenadine hydrochloride; pseudoephedrine hydrochloride	21704	9	1	5	3
ALORA	estradiol	20655	4	0	2	2
ALSUMA	sumatriptan succinate	22377	1	0	0	1
ALVESCO	ciclesonide	21658	10	1	5	4
AMELUZ	aminolevulinic acid hydrochloride	208081	3	0	2	1
ANDRODERM	testosterone	20489	6	0	2	4
ANNOVERA	ethinyl estradiol; segesterone acetate	209627	7	0	3	4
ANORO ELLIPTA	umeclidinium bromide-vilanterol trifenetate	203975	17	4	6	7
APIDRA SOLOSTAR	insulin glulisine recombinant	21629	23	1	4	18
ARCAPTA NEOHALER	indacaterol	22383	5	1	3	1
ARESTIN	minocycline hydrochloride	50781	2	0	0	2
ARIKAYCE KIT	amikacin sulfate	207356	12	0	11	1
ARMONAIR DIGIHALER	fluticasone	208798	31	0	1	30
ARNUITY ELLIPTA 100	fluticasone furoate	205625	9	1	1	7
ASMANEX TWISTHALER 220	mometasone	21067	11	0	6	5
ASPRUZYO SPRINKLE	ranolazine	216018	2	0	0	2
ATROVENT HFA	ipratropium bromide	21527	7	0	4	3
AUVI-Q	epinephrine	201739	32	0	2	30
AVAGARD	alcohol; chlorhexidine gluconate	21074	6	0	5	1
AXIRON	testosterone	22504	11	0	8	3
BAQSIMI	glucagon	210134	4	0	1	3
BELBUCA	buprenorphine hydrochloride	207932	5	0	2	3
BREO ELLIPTA 100	fluticasone-vilanterol	204275	17	3	7	7
BREZTRI AEROSPHERE	budesonide-formoterol-glycopyrrolate	212122	9	0	7	2
BUNAVAIL	buprenorphine hydrochloride; naloxone hydrochloride	205637	6	0	1	5
BUSPAR	buspirone hydrochloride	18731	6	0	3	3
BUTRANS	buprenorphine	21306	7	0	6	1
BYDUREON	exenatide synthetic	22200	38	3	25	10
BYDUREON BCISE	exenatide synthetic	209210	39	3	25	11
CAMCEVI KIT	leuprolide mesylate	211488	4	0	3	1
CANASA	mesalamine	21252	2	0	1	1

© 2025 Teng TW, et al. JAMA Health Forum.

CARAC	fluorouracil	20985	2	0	1	1
CARBATROL	carbamazepine	20712	3	0	1	2
CARDIZEM LA	diltiazem hydrochloride	21392	4	0	3	1
CARDURA XL	doxazosin mesylate	21269	1	0	0	1
CAVERJECT IMPULSE	alprostadil	21212	3	0	0	3
CERVIDIL	dinoprostone	20411	2	0	1	1
CHLORAPREP ONE-STEP FREPP	chlorhexidine gluconate; isopropyl alcohol	20832	13	0	1	12
CHLORAPREP ONE-STEP SEPP	chlorhexidine gluconate; isopropyl alcohol	21555	1	0	0	1
CHLORASCRUB MAXI SWABSTICK	chlorhexidine gluconate; isopropyl alcohol	21524	1	0	0	1
CHLORHEXIDINE GLUCONATE	chlorhexidine gluconate	21669	5	0	4	1
CLIMARA	estradiol	20375	1	0	0	1
CLIMARA PRO	estradiol; levonorgestrel	21258	4	0	1	3
CLINDESSE	clindamycin phosphate	50793	4	0	2	2
COMBIPATCH	estradiol	20870	4	0	0	4
COMBIVENT RESPIMAT	albuterol-ipratopium	21747	25	0	0	25
CONCERTA	methylphenidate hydrochloride	21121	11	0	7	4
COREG CR	carvedilol phosphate	22012	7	1	5	1
COVERA-HS	verapamil hydrochloride	20552	16	0	10	6
CRINONE	progesterone	20701	2	0	1	1
DAYTRANA	methylphenidate	21514	6	0	3	3
DEPOCYT	cytarabine	21041	5	0	2	3
DEPODUR	morphine sulfate	21671	10	0	7	3
DEXILANT / KAPIDEX	dexlansoprazole	22287	15	0	14	1
DEXTENZA	dexamethasone	208742	4	0	3	1
DEXYCU KIT	dexamethasone	208912	7	0	4	3
DILACOR XR	diltiazem hydrochloride	20092	2	0	0	2
DITROPAN XL	oxybutynin chloride	20897	10	0	5	5
DSUVIA	sufentanil citrate	209128	20	0	13	7
DUAKLIR PRESSAIR	aclidinium bromide-formoterol fumerate	210595	12	2	8	2
DUODOTE	atropine; pralidoxime chloride	21983	1	0	0	1
DURAGESIC	fentanyl	19813	3	0	0	3
DURYSTA	bimatoprost	211911	10	0	6	4
DYNACIRC CR	isradipine	20336	6	1	4	1
EFIDAC 24 CHLORPHENIRAMINE MALEAT	chlorpheniramine maleate	19746	3	0	1	2
EFIDAC 24 PSEUDOEPHEDRINE HCL	pseudoephedrine hydrochloride	20021	2	0	1	1
EFIDAC 24 PSEUDOEPHEDRINE HCL/BRO	brompheniramine maleate	19672	4	0	3	1
ELIGARD	leuprolide acetate	21731	17	0	9	8
ELIGARD KIT	leuprolide acetate	21343	13	0	6	7
ELIGARD KIT	leuprolide acetate	21379	17	0	9	8
ELIGARD KIT	leuprolide acetate	21488	17	0	9	8
EMEND	aprepitant	21549	8	1	6	1
EMSAM	selegiline	21336	4	0	3	1
ENSTILAR	betamethasone dipropionate; calcipotriene	207589	9	0	8	1

© 2025 Teng TW, et al. JAMA Health Forum.

EPIPEN/EPIPEN JR.	epinephrine	19430	5	0	0	5
EQUETRO	carbamazepine	21710	3	0	2	1
ESCLIM	estradiol	20847	1	0	0	1
ESKATA	hydrogen peroxide	209305	6	0	5	1
ESTRADERM	estradiol	19081	3	0	0	3
ESTRADIOL	estradiol	20538	8	0	4	4
ESTRASORB	estradiol hemihydrate	21371	1	0	0	1
ETHYOL	amifostine	20221	4	0	3	1
EVAMIST	estradiol	22014	4	0	1	3
EVZIO	naloxone hydrochloride	205787	33	0	1	32
EVZIO	naloxone hydrochloride	209862	33	0	1	32
EXPAREL	bupivacaine	22496	15	0	12	3
EXSERVAN	riluzole	212640	2	0	0	2
EXUBERA	insulin recombinant human	21868	13	0	8	5
EYSUVIS	loteprednol etabonate	210933	15	0	13	2
FARXIGA	dapagliflozin propanediol	202293	37	2	25	10
FAZACLO ODT	clozapine	21590	4	0	3	1
FEMPATCH	estradiol	20417	2	0	1	1
FEMRING	estradiol acetate	21367	1	0	0	1
FENSOLVI KIT	leuprolide acetate	213150	4	0	3	1
FIASP FLEXTOUCH	insulin aspart	208751	20	0	1	19
FINACEA	azelaic acid	207071	9	0	8	1
FINTEPLA	fenfluramine hydrochloride	212102	14	0	13	1
FLO-PRED	prednisolone acetate	22067	6	0	4	2
FLOVENT DISKUS 50	fluticasone propionate	20833	10	1	0	9
FLOVENT HFA 220	fluticasone propionate	21433	22	0	3	19
FLUTICASONE PROPIONATE	xhance	209022	19	0	6	13
FLUXID	famotidine	21712	2	0	1	1
FORADIL AEROLIZER	formoterol fumarate	20831	2	0	1	1
FORADIL CENTRIHALER	formoterol fumarate	21592	2	0	1	1
FORTEO	teriparatide recombinant human	21318	7	0	6	1
GELNIQUE	oxybutynin chloride	22204	7	0	5	2
GENOSYL	nitric oxide	202860	25	0	8	17
GENOTROPIN PRESERVATIVE FREE	somatropin recombinant	20280	6	0	1	5
GLIADEL	carmustine	20637	3	1	0	2
GLUCOTROL XL	glipizide	20329	8	0	5	3
GLYNASE	glyburide	20051	2	0	1	1
GYNAZOLE-1	butoconazole nitrate	19881	3	0	1	2
HABITROL	nicotine	20076	3	0	2	1
HEPZATO	melphalan hydrochloride	201848	10	0	3	7
HUMALOG KWIKPEN	insulin lispro recombinant	20563	3	2	0	1
HUMALOG KWIKPEN	insulin lispro recombinant	205747	3	0	2	1
HUMALOG MIX 50/50 KWIKPEN	insulin lispro protamine recombinant; insulin lispro recombinant	21018	5	2	2	1
HUMALOG MIX 75/25 KWIKPEN	insulin lispro protamine recombinant; insulin lispro recombinant	21017	5	2	2	1
HUMULIN 70/30 PEN	insulin recombinant human; insulin susp isophane recombinant human	19717	1	0	0	1
IDOSE TR	travoprost	218010	2	0	0	2
IGALMI	dexmedetomidine hydrochloride	215390	7	0	4	3
ILUVIEN	fluocinolone acetonide	201923	5	0	0	5
IMITREX	sumatriptan	20626	5	3	1	1

© 2025 Teng TW, et al. JAMA Health Forum.

IMPLANON	etonogestrel	21529	6	0	1	5
IMVEXXY	estradiol	208564	20	0	15	5
INBRIJA	levodopa	209184	18	0	14	4
INCRUSE ELLIPTA	umeclidinium bromide	205382	11	2	2	7
INOMAX	nitric oxide	20845	21	0	9	12
INTELENCE	etravirine	22187	4	2	1	1
INTRAROSA	prasterone	208470	3	0	2	1
INVEGA SUSTENNA	paliperidone palmitate	22264	5	1	3	1
INVELTYS	loteprednol etabonate	210565	11	0	9	2
IONSYS	fentanyl hydrochloride	21338	24	0	7	17
JELMYTO	mitomycin	211728	2	0	0	2
KEMSTRO	baclofen	21589	2	0	1	1
KYLEENA	levonorgestrel	208224	6	0	1	5
KYNMOBI	apomorphine hydrochloride	210875	19	0	11	8
LANTUS	insulin glargine recombinant	21081	23	2	3	18
LAZANDA	fentanyl citrate	22569	6	0	4	2
LEVEMIR FLEXTOUCH	insulin detemir recombinant	21536	27	3	1	23
LEVULAN	aminolevulinic acid hydrochloride	20965	14	0	12	2
LIDODERM	lidocaine	20612	6	0	5	1
LIDOSITE TOPICAL SYSTEM KIT	epinephrine; lidocaine hydrochloride	21504	7	0	0	7
LILETTA	levonorgestrel	206229	3	0	1	2
LONHALA MAGNAIR KIT	glycopyrrolate	208437	14	0	1	13
LOPRESSOR	metoprolol fumarate	19786	5	1	1	3
LUPANETA PACK	leuprolide acetate; norethindrone acetate	203696	1	0	0	1
LUPRON DEPOT	leuprolide acetate	19732	16	1	2	13
LUPRON DEPOT	leuprolide acetate	20517	19	0	5	14
LUPRON DEPOT	leuprolide acetate	20011	15	1	2	12
LUPRON DEPOT-3	leuprolide acetate	20708	16	0	2	14
LUPRON/LUPRON DEPOT	leuprolide acetate	20263	18	1	4	13
LUTRATE DEPOT KIT	leuprolide acetate	205054	1	0	0	1
MACROBID	nitrofurantoin	20064	2	0	1	1
MACUGEN	pegaptanib sodium	21756	7	2	4	1
MAKENA (AUTOINJECTOR)	hydroxyprogesterone caproate	21945	13	0	4	9
MAXAIR AUTOHALER	pirbuterol acetate	20014	1	0	0	1
MEGACE ES	megestrol acetate	21778	7	0	6	1
MENOSTAR	estradiol	21674	3	0	2	1
MIGRANAL	dihydroergotamine mesylate	20148	3	0	2	1
MILPROSA	progesterone	201110	3	0	2	1
MINIPRESS XL	prazosin hydrochloride	19775	6	0	1	5
MINIVELLE	estradiol	203752	7	0	2	5
MIRENA	levonorgestrel	21225	5	0	1	4
MITOSOL	mitomycin	22572	5	0	1	4
MORPHINE SULFATE	morphine sulfate	204223	3	0	2	1
MOUNJARO	tirzepatide	215866	4	1	1	2
MUSE	alprostadil	20700	5	0	3	2
NALOXONE HYDROCHLORIDE (AUTOINJECTOR)	naloxone hydrochloride	215457	20	0	1	19
NARCAN	naloxone hydrochloride	208411	8	0	7	1
NAROPIN	ropivacaine hydrochloride	20533	8	0	4	4

© 2025 Teng TW, et al. JAMA Health Forum.

NASCOBAL	cyanocobalamin	21642	6	0	5	1
NEUPRO	rotigotine	21829	9	0	3	6
NICODERM CQ	nicotine	20165	12	0	2	10
NICOTROL	nicotine	20714	7	0	0	7
NICOTROL	nicotine	20536	3	0	1	2
NIRAVAM	alprazolam	21726	2	0	1	1
NOCTIVA	desmopressin acetate	201656	5	0	4	1
NORDITROPIN NORDIFLEX	somatropin recombinant	21148	28	0	4	24
NOVOLOG MIX 70/30 FLEXPEN	insulin aspart protamine recombinant; insulin aspart recombinant	21172	12	1	5	6
NOVOLOG PENFILL	insulin aspart recombinant	20986	24	1	1	22
NUTROPIN DEPOT	somatropin recombinant	21075	4	0	3	1
NUVARING	ethinyl estradiol; etonogestrel	21187	1	0	0	1
NYMALIZE	nimodipine	203340	8	0	5	3
OLUX E	clobetasol propionate	22013	4	0	2	2
ONIVYDE	irinotecan hydrochloride	207793	17	0	16	1
ONPATTRO	patisiran sodium	210922	24	8	8	8
ONSOLIS	fentanyl citrate	22266	3	0	1	2
ONZETRA XSAIL	sumatriptan succinate	206099	20	0	7	13
OPANA ER	oxymorphone hydrochloride	21610	8	0	6	2
ORAPRED ODT	prednisolone sodium phosphate	21959	4	0	3	1
ORTHO EVRA	ethinyl estradiol; norelgestromin	21180	2	0	0	2
OSMOLEX ER	amantadine hydrochloride	209410	18	0	15	3
OTIPRIO	ciprofloxacin	207986	8	0	7	1
OTREXUP	methotrexate	204824	19	0	2	17
OXYTROL	oxybutynin	21351	9	0	6	3
OXYTROL FOR WOMEN	oxybutynin	202211	8	0	6	2
OZEMPIC	semaglutide	209637	25	2	2	21
OZURDEX	dexamethasone	22315	18	0	13	5
PAXIL CR	paroxetine hydrochloride	20936	13	1	10	2
PEPCID AC	famotidine	20902	4	1	2	1
POMALYST	pomalidomide	204026	20	1	18	1
PRADAXA	dabigatran etexilate mesylate	22512	5	1	3	1
PROAIR HFA	albuterol sulfate	21457	17	0	6	11
PROAIR RESPICLICK	albuterol sulfate	205636	28	0	1	27
PROCANBID	procainamide hydrochloride	20545	1	0	0	1
PROCARDIA XL	nifedipine	19684	7	0	1	6
PROVENTIL HFA	albuterol	20503	8	0	6	2
PULMICORT 160	budesonide	20441	3	0	0	3
PULMICORT FLEXHALER 80	budesonide	21949	5	0	2	3
PULMICORT RESPULES	budesonide	20929	4	0	3	1
QBREXZA	glycopyrronium tosylate	210361	10	0	9	1
QNASL	beclomethasone dipropionate	202813	5	0	3	2
QUTENZA	capsaicin	22395	8	0	5	3
QVAR 80	beclomethasone dipropionate	20911	15	0	6	9
QVAR REDIHALER	beclomethasone dipropionate	207921	15	0	2	13
RAPAMUNE	sirolimus	21110	6	0	5	1
REGLAN ODT	metoclopramide hydrochloride	21793	2	0	1	1
RELENZA	zanamivir	21036	8	2	1	5
RENOVA	tretinoin	19963	4	0	3	1
REQUIP XL	ropinirole hydrochloride	22008	3	0	2	1
RESTASIS MULTIDOSE	cyclosporine	50790	12	0	8	4

© 2025 Teng TW, et al. JAMA Health Forum.

RETIN-A MICRO	tretinoin	20475	2	0	1	1
RETISERT	fluocinolone acetonide	21737	2	0	0	2
REVLIMID	lenalidomide	21880	30	2	27	1
RISPERDAL CONSTA	risperidone	21346	19	1	6	12
RIZAFILM	rizatriptan benzoate	205394	1	0	0	1
ROWASA	mesalamine	19618	3	0	2	1
RYALTRIS	mometasone furoate; olopatadine hydrochloride	211746	14	0	12	2
RYKINDO	risperidone	212849	5	0	3	2
RYTHMOL SR	propafenone hydrochloride	21416	1	0	0	1
RYZODEG 70/30	insulin aspart; insulin degludec	203313	22	1	2	19
SANCUSO	granisetron	22198	1	0	0	1
SANDOSTATIN LAR	octreotide acetate	21008	7	3	1	3
SAXENDA	liraglutide recombinant	206321	28	3	3	22
SECUADO	asenapine	212268	6	0	1	5
SEEBRI NEOHALER	glycopyrrolate	207923	14	0	7	7
SEREVENT DISKUS	salmeterol xinafoate	20692	15	2	3	10
SIGNIFOR LAR	pasireotide pamoate	203255	5	2	2	1
SINUVA	mometasone furoate	209310	13	0	10	3
SKYLA	levonorgestrel	203159	7	0	1	6
SOLIQUA 100/33	insulin glargine/lixisenatide	208673	26	1	7	18
SPIRIVA HANDIHALER	tiotropium bromide monohydrate	21395	12	2	6	4
SPIRIVA RESPIMAT 25	tiotropium bromide	21936	19	1	0	18
SPORANOX	itraconazole	20083	3	2	0	1
STIOLTO RESPIMAT	olodaterol hydrochloride-tiotropium bromide	206756	26	3	5	18
STRIANT	testosterone	21543	2	0	1	1
STRIVERDI RESPIMAT	olodaterol	203108	25	2	5	18
SUBLOCADE	buprenorphine	209819	12	0	11	1
SUBOXONE	buprenorphine hydrochloride; naloxone hydrochloride	22410	8	0	2	6
SULAR	nisoldipine	20356	5	1	3	1
SUMAVEL DOSEPRO	sumatriptan succinate	22239	16	0	3	13
SUPPRELIN LA	histrelin acetate	22058	3	0	1	2
SUPRANE	desflurane	20118	2	0	1	1
SUSTOL	granisetron	22445	7	0	6	1
SYMBICORT	budesonide-formoterol fumarate	21929	19	0	11	8
SYMJEPI	epinephrine	207534	1	0	0	1
SYMPAZAN	clobazam	210833	3	0	0	3
SYMTUZA	cobicistat; darunavir ethanolate; emtricitabine; tenofovir alafenamide fumarate	210455	15	4	10	1
SYNERA	lidocaine; tetracaine	21623	7	0	1	6
TECZEM	diltiazem malate; enalapril maleate	20507	5	1	2	2
TEGRETOL-XR	carbamazepine	20234	2	0	0	2
TESTODERM	testosterone	19762	4	0	1	3
TESTODERM TTS	testosterone	20791	2	0	1	1
THALOMID	thalidomide	20785	20	0	19	1
TIAMATE	diltiazem malate	20506	2	0	0	2
TOBI PODHALER	tobramycin	201688	14	0	6	8
TOPROL-XL	metoprolol succinate	19962	5	1	3	1
TOUJEO SOLOSTAR	insulin glargine recombinant	206538	20	0	2	18
TRELEGY ELLIPTA	fluticasone-umeclidinium-vilanterol	209482	20	4	10	6
TRELSTAR	triptorelin pamoate	21288	3	0	2	1

© 2025 Teng TW, et al. JAMA Health Forum.

TRELSTAR DEPOT	triptorelin pamoate	20715	4	0	3	1
TRESIBA	insulin degludec	203314	20	1	0	19
TRICOR	fenofibrate	21656	8	0	7	1
TRUDHESA	dihydroergotamine mesylate	213436	6	0	1	5
TUDORZA PRESSAIR	aclidinium bromide	202450	13	3	6	4
TWINJECT 0.3	epinephrine	20800	6	0	0	6
TWIRLA	ethinyl estradiol; levonorgestrel	204017	8	0	2	6
TWYNEO	benzoyl peroxide; tretinoin	214902	5	0	3	2
TYVASO	treprostinil	22387	13	2	10	1
TYVASO DPI	treprostinil	214324	8	0	7	1
ULTANE	sevoflurane	20478	4	0	3	1
UTIBRON NEOHALER	indacaterol-glycopyrrolate	207930	20	1	12	7
UZEDY	risperidone	213586	9	0	4	5
VAGIFEM	estradiol	20908	2	0	1	1
VANTAS	histrelin acetate	21732	2	0	0	2
VARITHENA	polidocanol	205098	16	0	8	8
VENTOLIN HFA	albuterol	20983	21	0	3	18
VENTOLIN ROTACAPS	albuterol	19489	4	1	1	2
VERAMYST	fluticasone furoate	22051	8	1	2	5
VERDESO	desonide	21978	5	0	3	2
VIADUR	leuprolide acetate	21088	10	0	3	7
VICTOZA	liraglutide recombinant	22341	12	3	3	6
VITRASERT	ganciclovir	20569	1	0	0	1
VIVITROL	naltrexone	21897	20	0	5	15
VOLMAX	albuterol sulfate	19604	3	0	2	1
VYXEOS	cytarabine; daunorubicin	209401	9	0	8	1
XELSTRYM	dextroamphetamine	215401	6	0	3	3
XIPERE	triamcinolone acetonide	211950	3	0	2	1
XOPENEX HFA	levalbuterol	21730	13	1	11	1
XTAMPZA ER	oxycodone	208090	19	0	18	1
XULTOPHY 100/3.6	insulin degludec/liraglutide	208583	25	4	2	19
XYOSTED (AUTOINJECTOR)	testosterone enanthate	209863	25	1	6	18
YCANTH	cantharidin	212905	2	0	1	1
YUTIQ	fluocinolone acetonide	210331	10	0	2	8
ZECUITY	sumatriptan succinate	202278	10	0	5	5
ZEPBOUND	tirzepatide	217806	4	1	1	2
ZETONNA	ciclesonide	202129	9	1	4	4
ZIMHI	naloxone hydrochloride	212854	2	0	1	1
ZINGO	lidocaine hydrochloride	22114	7	0	2	5
ZOLADEX	goserelin acetate	19726	6	1	2	3
ZOLADEX	goserelin acetate	20578	6	1	2	3
ZTALMY	ganaxolone	215904	8	0	6	2
ZTLIDO	lidocaine	207962	8	0	4	4
ZUPLENZ	ondansetron	22524	2	0	1	1
ZURAGARD	isopropyl alcohol	210872	7	0	5	2
ZYFLO CR	zileuton	22052	3	1	1	1
ZYNRELEF KIT	bupivacaine; meloxicam	211988	16	0	12	4

FDA: Food and Drug Administration; NDA: New Drug Application.

© 2025 Teng TW, et al. JAMA Health Forum.

eTable 5: Products with portfolios consisting solely of tertiary patents

Brand name	Generic name	NDA	Tertiary patents
ALSUMA	sumatriptan succinate	22377	1
ARESTIN	minocycline hydrochloride	50781	2
ASPRUZYO SPRINKLE	ranolazine	216018	2
CARDURA XL	doxazosin mesylate	21269	1
CAVERJECT IMPULSE	alprostadil	21212	3
CHLORAPREP ONE-STEP SEPP	chlorhexidine gluconate; isopropyl alcohol	21555	1
CHLORASCRUB MAXI SWABSTICK	chlorhexidine gluconate; isopropyl alcohol	21524	1
CLIMARA	estradiol	20375	1
COMBIPATCH	estradiol	20870	4
COMBIVENT RESPIMAT	albuterol-ipratopium	21747	25
DILACOR XR	diltiazem hydrochloride	20092	2
DUODOTE	atropine; pralidoxime chloride	21983	1
DURAGESIC	fentanyl	19813	3
EPIPEN/EPIPEN JR.	epinephrine	19430	5
ESCLIM	estradiol	20847	1
ESTRADERM	estradiol	19081	3
ESTRASORB	estradiol hemihydrate	21371	1
EXSERVAN	riluzole	212640	2
FEMRING	estradiol acetate	21367	1
HUMULIN 70/30 PEN	insulin recombinant human; insulin susp isophane recombinant human	19717	1
IDOSE TR	travoprost	218010	2
ILUVIEN	fluocinolone acetonide	201923	5
JELMYTO	mitomycin	211728	2
LIDOSITE TOPICAL SYSTEM KIT	epinephrine; lidocaine hydrochloride	21504	7
LUPANETA PACK	leuprolide acetate; norethindrone acetate	203696	1
LUTRATE DEPOT KIT	leuprolide acetate	205054	1
MAXAIR AUTOHALER	pirbuterol acetate	20014	1
NICOTROL	nicotine	20714	7
NUVARING	ethinyl estradiol; etonogestrel	21187	1
ORTHO EVRA	ethinyl estradiol; norelgestromin	21180	2
PROCANBID	procainamide hydrochloride	20545	1
PULMICORT 160	budesonide	20441	3
RETISERT	fluocinolone acetonide	21737	2
RIZAFILM	rizatriptan benzoate	205394	1
RYTHMOL SR	propafenone hydrochloride	21416	1
SANCUSO	granisetron	22198	1
SYMJEPI	epinephrine	207534	1
SYMPAZAN	clobazam	210833	3
TEGRETOL-XR	carbamazepine	20234	2
TIAMATE	diltiazem malate	20506	2
TWINJECT 0.3	epinephrine	20800	6
VANTAS	histrelin acetate	21732	2
VITRASERT	ganciclovir	20569	1

© 2025 Teng TW, et al. JAMA Health Forum.

eTable 6: Patents filed after FDA approval

NDA	Total patents filed after NDA approval	Primary patents	Secondary patents	Tertiary patents
202971	6	0	5	1
207202	2	0	0	2
21660	13	0	13	0
22549	6	0	0	6
212038	4	0	4	0
208471	2	0	2	0
21077	5	0	1	4
21254	2	0	0	2
22472	6	0	2	4
208799	2	0	0	2
21704	1	0	1	0
208081	1	0	0	1
209627	7	0	3	4
203975	2	0	2	0
21629	21	0	3	18
22383	2	0	2	0
50781	2	0	0	2
207356	2	0	2	0
208798	11	0	0	11
21067	1	0	1	0
21527	1	0	0	1
201739	16	0	2	14
21074	3	0	3	0
22504	5	0	4	1
210134	1	0	0	1
207932	1	0	0	1
212122	1	0	0	1
205637	1	0	0	1
18731	3	0	2	1
21306	1	0	1	0
22200	8	0	6	2
21252	2	0	1	1
20985	1	0	1	0
20712	1	0	0	1
20832	7	0	1	6
21669	4	0	3	1
50793	1	0	1	0
21121	6	0	6	0
20552	1	0	1	0
21514	3	0	1	2
21041	1	0	1	0
22287	6	0	5	1
208912	3	0	1	2
20092	1	0	0	1
20897	2	0	2	0
209128	1	0	0	1
21731	7	0	4	3
21343	3	0	0	3

© 2025 Teng TW, et al. JAMA Health Forum.

21379	7	0	3	4
21488	7	0	3	4
207589	5	0	5	0
19430	5	0	0	5
209305	2	0	1	1
19081	1	0	0	1
20538	1	0	0	1
20221	1	0	1	0
205787	13	0	1	12
209862	8	0	1	7
22496	12	0	11	1
210933	2	0	2	0
202293	3	0	3	0
213150	1	0	0	1
208751	1	0	0	1
207071	1	0	1	0
212102	3	0	3	0
20833	4	0	0	4
21433	6	0	0	6
209022	2	0	0	2
20831	1	0	1	0
21318	4	0	3	1
22204	4	0	2	2
202860	2	0	1	1
20280	1	0	0	1
20329	2	0	2	0
19881	1	0	1	0
20076	1	0	1	0
20563	1	0	0	1
21018	1	0	0	1
21017	1	0	0	1
19717	1	0	0	1
215390	2	0	2	0
21529	4	0	0	4
208564	11	0	8	3
20845	15	0	7	8
210565	3	0	3	0
21338	7	0	2	5
208224	2	0	1	1
21081	21	0	3	18
22569	4	0	2	2
21536	18	0	0	18
20965	10	0	9	1
206229	2	0	1	1
208437	2	0	1	1
19732	9	0	1	8
20517	7	0	3	4
20011	8	0	1	7
20708	1	0	0	1
20263	10	0	3	7
21945	12	0	4	8
21778	3	0	3	0
203752	2	0	0	2

© 2025 Teng TW, et al. JAMA Health Forum.

21225	4	0	1	3
22572	3	0	1	2
204223	3	0	2	1
20700	1	0	1	0
208411	6	0	6	0
20533	5	0	1	4
21642	4	0	4	0
21829	5	0	3	2
20165	9	0	2	7
20536	1	0	0	1
21148	23	0	1	22
21172	5	0	0	5
20986	19	0	0	19
203340	6	0	5	1
22013	1	0	1	0
207793	8	0	8	0
210922	1	0	0	1
22266	1	0	0	1
206099	3	0	2	1
21610	3	0	3	0
209410	4	0	4	0
207986	3	0	2	1
204824	9	0	0	9
21351	5	0	5	0
209637	3	0	0	3
22315	9	0	6	3
20936	3	0	3	0
204026	4	0	4	0
22512	1	0	1	0
21457	10	0	0	10
205636	17	0	0	17
19684	1	0	1	0
20503	2	0	2	0
20929	2	0	2	0
210361	1	0	1	0
202813	1	0	0	1
22395	5	0	4	1
20911	8	0	0	8
207921	7	0	2	5
19963	1	0	1	0
22008	1	0	1	0
50790	10	0	6	4
21880	16	1	14	1
21346	1	0	1	0
19618	2	0	1	1
203313	3	0	0	3
21008	1	0	0	1
206321	8	0	1	7
212268	1	0	0	1
207923	1	0	0	1
20692	7	0	0	7
203159	4	0	1	3
21395	5	1	1	3

© 2025 Teng TW, et al. JAMA Health Forum.

20083	1	0	0	1
209819	4	0	4	0
22410	5	0	1	4
22239	5	0	2	3
20118	1	0	0	1
22445	2	0	2	0
21929	10	0	5	5
207534	1	0	0	1
210833	1	0	0	1
210455	1	0	1	0
19762	1	0	0	1
20791	1	0	1	0
20785	18	0	17	1
201688	3	0	1	2
19962	1	0	0	1
206538	5	0	0	5
203314	3	0	0	3
202450	7	1	6	0
20800	5	0	0	5
22387	9	1	7	1
20478	4	0	3	1
207930	1	0	0	1
20908	1	0	1	0
20983	11	0	0	11
22051	2	0	0	2
21978	2	0	2	0
21088	1	0	0	1
22341	3	0	2	1
21897	1	0	1	0
209401	2	0	2	0
21730	1	0	1	0
208090	8	0	7	1
208583	2	0	0	2
209863	7	0	2	5
202278	2	0	1	1
22114	3	0	1	2
19726	4	0	1	3
20578	3	0	0	3
207962	3	0	2	1
22524	1	0	1	0

FDA: Food and Drug Administration; NDA: New Drug Application.

© 2025 Teng TW, et al. JAMA Health Forum.

eTable 7: Patent protection on drug-device combinations by route of administration

Route of administration	Number of products	Median expected patent protection (IQR)	Median realized patent protection (IQR)
Other	19	19.7 (16.4-25.9)	18.8 (16.3-23.5)
Injection	82	19.7 (15.6-25.2)	19.2 (15.4-23.6)
Intranasal	14	18.6 (16.6-19.6)	17.9 (16.2-19.4)
Inhalation	56	18.0 (15.4-21.7)	17.2 (13.9-20.1)
Oral	75	17.5 (13.5-20.0)	13.5 (10.5-17.1)
Intravaginal	11	16.5 (15.0-20.8)	16.1 (15.0-17.4)
Topical	60	16.4 (14.0-19.9)	15.1 (11.5-19.7)
Ophthalmic	14	13.4 (11.2-14.7)	13.4 (11.2-14.7)

IQR: interquartile range

© 2025 Teng TW, et al. JAMA Health Forum.

eTable 8: Patent protection on drug-device combinations by therapeutic class

Therapeutic class	Number of products	Median expected patent protection (IQR)	Median realized patent protection (IQR)
L – Antineoplastic and immunomodulating agents	37	19.8 (15.9-27.9)	19.1 (15.1-26.3)
A – Alimentary tract and metabolism	41	19.1 (15.6-26.2)	17.1 (14.4-22.6)
Other	31	18.4 (14.5-20.8)	16.6 (13.4-20.1)
C – Cardiovascular system	23	18.3 (13.0-20.6)	14.4 (11.0-18.9)
N – Nervous system	70	17.4 (14.6-20.5)	16.4 (12.4-19.8)
R – Respiratory system	52	17.3 (15.0-20.4)	16.8 (13.2-20.1)
D – Dermatologicals	18	17.0 (14.2-19.5)	17.0 (14.2-19.5)
G – Genitourinary system and sex hormones	43	16.2 (13.7-20.2)	15.7 (12.0-18.8)
S – Sensory organs	16	14.2 (12.5-16.6)	14.2 (12.5-16.6)

© 2025 Teng TW, et al. JAMA Health Forum.

References

1. US Food and Drug Administration. Orange Book Questions and Answers. Available online from: https://www.fda.gov/media/160167/download#:~. Accessed October 14, 2025
2. 21 U.S.C. § 355. Available online from: https://www.govinfo.gov/app/details/USCODE-2023-title21/USCODE-2023-title21-chap9-subchapV-partA-sec355. Accessed October 14, 2025.
3. Federal Trade Commission. FTC Issues Policy Statement on Brand Pharmaceutical Manufacturers' Improper Listing of Patents in the Food and Drug Administration's 'Orange Book'. Available online from: https://www.ftc.gov/news-events/news/press-releases/2023/09/ftc-issues-policy-statement-brand-pharmaceutical-manufacturers-improper-listing-patents-food-drug. Accessed October 14, 2025.
4. Centers for Medicare and Medicaid Services. Medicaid Drug Rebate Program (MDRP). Available online from: https://www.medicaid.gov/medicaid/prescription-drugs/medicaid-drug-rebate-program. Accessed October 14, 2025.

© 2025 Teng TW, et al. JAMA Health Forum.
