# Supplementary material for: Tertiary Patents on Drugs Approved by the FDA
Source: JAMA Health Forum. 2026 Jan 2;7(1):e255909. doi: 10.1001/jamahealthforum.2025.5909 (PMC12761334; doi:10.1001/jamahealthforum.2025.5909)
Supplement: Supplement 2. — Data Sharing Statement [file jamahealthforum-e255909-s002.pdf]

Data Sharing Statement

Data
Data available: Yes
Data types: Data (not involving human participants)
How to access data: wfeldman@mednet.ucla.edu
When available: With publication

Supporting Documents
Document types: None

Additional Information
Who can access the data: Researchers whose proposed use if the data has been approved.
Types of analyses: For research purposes.
Mechanisms of data availability: After approval of a proposal.
